# SIRT7-mediated desuccinylation of FOXO4 suppresses ferroptosis to alleviate LPS-induced acute lung injury

**DOI:** 10.1016/j.redox.2026.104315

**Published:** 2026-07-18

**Authors:** Kaikai Shen, Yuqing Wei, Hao Xu, Zhangmin Ke, He Zhang, Xinyu Zhou, Peilin Chen, Ping Zhan, Fang Zhang, Suhua Zhu, Jiajia Jin, Tangfeng Lv, Yong Song

**Affiliations:** a Department of Respiratory and Critical Care Medicine, Jinling Hospital, Affiliated Hospital of Medical School, Nanjing University, Nanjing, China; b Department of Respiratory and Critical Care Medicine, The First Affiliated Hospital of Wannan Medical University (Yijishan Hospital of Wannan Medical University), Wuhu, China; c Department of Respiratory and Critical Care Medicine, The People's Hospital of Danyang, Affiliated Danyang Hospital of Nantong University, Danyang, China; d Department of Respiratory and Critical Care Medicine, Affiliated Jiangning Hospital of Nanjing Medicine University, Nanjing, China

**Keywords:** SIRT7, ALI, FOXO4, Succinylation, Ferroptosis

## Abstract

Protein succinylation, an emerging post-translational modification (PTM), assumes a crucial role in the initiation and advancement of inflammatory diseases. Ferroptosis, propelled by lethal lipid peroxidation, is intricately associated with the pathogenesis of inflammation. Targeting ferroptosis has recently emerged as a promising therapeutic approach for acute lung injury (ALI). Nevertheless, the crosstalk between protein succinylation and ferroptosis in the regulation of ALI remains ambiguous. FOXO4, a key regulator of oxidative stress responses, is dynamically regulated by various PTMs. We identify SIRT7 as the desuccinylase essential for FOXO4 activation and nuclear localization. Mechanistically, SIRT7 desuccinylates FOXO4 at lysine 139 (K139), thereby inhibiting MDM2-mediated K48-linked polyubiquitination, which stabilizes FOXO4 and maintains FOXO4 nuclear retention. This consequently upregulates glutathione peroxidase 4 (GPX4) and suppresses lipopolysaccharide (LPS)-induced ferroptosis in alveolar epithelial cells (AECs). In vivo experiments demonstrated that SIRT7-knockout (SIRT7-KO) exacerbates LPS-induced ALI. Furthermore, the delivery of a FOXO4-K139R (mimicking desuccinylation) via adeno-associated virus 6 (AAV6) significantly alleviated pulmonary ferroptosis and histopathological damage in SIRT7-KO mice. Notably, pharmacological activation of SIRT7 with trilobatin (TLB) significantly attenuates ALI in LPS-challenged mice, establishing a potential therapeutic pathway for this pathology. Collectively, these findings delineate a previously unrecognized mechanism through which SIRT7 governs the nuclear retention and activation of FOXO4 via desuccinylation, establishing the SIRT7-FOXO4 axis as a potential therapeutic target and theoretical basis for ALI intervention.

## 1 Introduction

Acute lung injury (ALI) and its severe form, acute respiratory distress syndrome (ARDS), are severe, diffuse inflammatory lung disorders arising from a wide range of direct or indirect insults, including systemic inflammatory conditions (e.g., sepsis, major trauma), infectious agents (e.g., viral or bacterial pathogens), and inhalational exposures (e.g., smoke, toxic gases) [1,2]. Although considerable progress has been made over the past decade in elucidating infection-associated immune dysregulation, the clinical management of ALI remains challenging. The rapidly evolving nature and pathophysiological complexity of emerging infectious diseases, together with the growing threat of antimicrobial resistance, have impeded the development and widespread adoption of evidence-based, disease-modifying therapies [3]. As a result, ALI continues to be associated with high mortality, estimated at approximately 40% in current clinical practice [4].

Alveolar epithelial cells (AECs) serve as critical structural components of the pulmonary blood-gas barrier and play essential roles in preserving alveolar architectural integrity, regulating alveolar fluid balance, and synthesizing and secreting pulmonary surfactant. Under pathological conditions, injury and dysfunction of these cells constitute key events in the initiation and progression of ALI [5]. Ferroptosis, a recently recognized form of regulated cell death driven by iron-dependent lipid peroxidation, differs fundamentally from classical cell death pathways such as apoptosis and necrosis in morphological, biochemical, and genetic aspects [6]. Growing evidence indicates that ferroptosis in AECs represents an important pathophysiological mechanism in ALI. In experimental models, including lipopolysaccharide (LPS)-induced endotoxemia and renal ischemia/reperfusion (I/R)-induced lung injury, AECs exhibit characteristic features of ferroptosis, such as downregulation of glutathione peroxidase 4 (GPX4) and accumulation of lipid peroxides [7,8]. Targeting ferroptosis in these cells demonstrates efficacy in alleviating both pulmonary inflammatory responses and oxidative stress, thereby mitigating ALI pathology [8,9].

Post-translational modifications (PTMs) represent a crucial category of regulatory mechanisms that dynamically fine-tune protein function, activity, localization, and stability after ribosomal synthesis. Lysine succinylation, a reversible and evolutionarily conserved PTM widespread in mammals, entails the covalent addition of a succinyl moiety to the ε-amino group of lysine residues and occurs through enzymatic pathways or non-enzymatic mechanisms. Enzymatic succinylation involves transfer of a succinyl group from succinyl-CoA to this site, catalyzed by specific succinyltransferases (e.g., p300/CBP, CPT1A, KAT2A, HAT1, and OXCT1). This modification relies on enzyme catalytic activity, exhibits substrate and site specificity, and is typically regulated by cellular signaling or metabolic cues [[10], [11], [12]]. In contrast, non-enzymatic succinylation occurs spontaneously via direct chemical reaction between succinyl-CoA and lysine ε-amino groups under physiological conditions. The reaction proceeds through nucleophilic attack of the lysine side chain on the succinyl carbonyl carbon, forming an amide bond. The rate of non-enzymatic succinylation is modulated by local pH, temperature, succinyl-CoA concentration, and the microenvironment of individual lysine residues [11]. Modified substrates consist primarily of histones, which critically regulate gene transcription, and nonhistone proteins, particularly metabolic enzymes. These modifications alter protein conformation and functional activity, thereby influencing key physiological processes, including energy metabolism and signal transduction. Compared with enzymatic succinylation, non-enzymatic succinylation generally lacks strict specificity and may broadly target lysine residues exposed to metabolite-rich environments; however, it can also predominate at evolutionarily conserved hyperreactive sites. Mounting evidence underscores the involvement of succinylation in the pathogenesis and regulation of inflammatory diseases. For instance, Zhang et al. observed a marked increase in pan-succinylation within hepatic tissues of mice with sepsis-induced acute liver injury, implying its active role in disease pathogenesis [13]. Fu et al. revealed that γ-aminobutyric acid alleviates macrophage-driven inflammation by curbing mitochondrial protein succinylation, consequently boosting oxidative phosphorylation-an insight that positions succinylation as a viable therapeutic target [14]. Moreover, investigations in *Streptomyces coelicolor* demonstrated that the NAD ^+^ -dependent desuccinylase ScCobB2 modulates ribosomal biogenesis and central carbon metabolism, highlighting the conserved regulatory role of succinylation across phylogeny [15]. Taken together, these observations affirm lysine succinylation as a pivotal, context-sensitive modulator of inflammatory responses. Nevertheless, the exact molecular mediators, downstream signaling pathways, and tissue-specific functional ramifications remain elusive and merit in-depth exploration.

Succinylation represents a reversible PTM that dynamically regulates protein structure, activity, and interactions. Cellular succinylation homeostasis is maintained by the coordinated actions of succinyltransferases and desuccinylases, primarily SIRT5 and SIRT7 [12,16]. Our investigation revealed significantly elevated pan-succinylation levels in lung tissues from ALI models compared to healthy controls. Notably, SIRT7 mRNA expression demonstrated a specific inverse correlation with this pan-succinylation increase, suggesting its potential role as a key regulatory desuccinylase in ALI pathogenesis. As the sole nucleolus-enriched, NAD^+^-dependent deacetylase, SIRT7 has recently emerged as an important regulator of non-histone modifications [17,18]. Previous studies have shown that SIRT7-mediated deacetylation of NRF2 attenuates chemical-induced liver injury [18], and that SIRT7-dependent desuccinylation of MG53 at K130 ameliorates cardiomyocyte injury following hypoxia/reoxygenation [19]. Cheng and colleagues elucidated a neuroprotective mechanism wherein SIRT7 attenuates ischemic brain injury by catalyzing nicotinamide phosphoribosyltransferase (NAMPT) desuccinylation [20]. However, the precise mechanisms by which SIRT7 functions in ALI through the regulation of protein succinylation remain to be elucidated. To investigate the downstream targets of SIRT7 in the context of ALI, an immunoprecipitation-mass spectrometry (IP-MS) analysis was conducted. FOXO4 was identified as a high-confidence binding partner of SIRT7 through this screening. FOXO4, a core transcription factor, regulates diverse cellular processes including metabolic adaptation, oxidative stress response, and programmed cell death through multiple PTMs such as phosphorylation, ubiquitination, and acetylation [[21], [22], [23], [24], [25], [26], [27], [28]]. Nevertheless, the role of SIRT7-mediated FOXO4 desuccinylation in AECs injury during ALI remains unexplored.

Here, we indicate that SIRT7 directly binds FOXO4 and desuccinylates it at lysine 139 (K139), thereby inhibiting MDM2-mediated K48-linked polyubiquitination, leading to FOXO4 stabilization, enhanced nuclear retention, and transcriptional upregulation of GPX4. Consequently, the SIRT7-FOXO4 axis inhibits LPS-induced ferroptosis in AECs and therapy mitigates ALI. Moreover, adeno-associated virus 6 (AAV6)-mediated delivery of the desuccinylation-mimetic FOXO4-K139R markedly attenuated histopathological injury in the SIRT7-knockout (KO) mice. Our findings further demonstrate that trilobatin (TLB), a potential SIRT7 agonist, significantly attenuates LPS-induced ALI in murine models. Overall, our study identifies the SIRT7-FOXO4 axis as a pivotal regulator of redox homeostasis in ALI, underscoring its therapeutic potential for ALI.

## 2 Materials and methods

### 2.1 Patients

This study enrolled adult patients (≥18 years) meeting the 2012 Berlin definition of ARDS [29]. Exclusion criteria consisted of chronic liver disease, autoimmune disorders, chronic kidney disease, or active malignancy. Bronchoalveolar lavage fluid (BALF) was collected prospectively within 24 h of hospitalization from eligible ARDS patients and matched healthy controls. Baseline demographic and clinical characteristics are presented in Table S1. ARDS severity was classified per Berlin criteria as mild (200 < PaO_2_/FiO_2_ ≤ 300 mmHg), moderate (100 < PaO_2_/FiO_2_ ≤ 200 mmHg), or severe (PaO_2_/FiO_2_ ≤ 100 mmHg). Disease severity was further assessed using the APACHE II score and oxygenation index (PaO_2_/FiO_2_), with all evaluations completed within 24 h following ARDS diagnosis. The study protocol received approval from the Ethics Committee of Jinling Hospital, Medical School of Nanjing University (Approval No. 2025DZGJJ-052) and conformed to the Declaration of Helsinki. Written informed consent was obtained from all participants or their legal representatives prior to enrollment.

### 2.2 Animals

Male SIRT7-KO mice and wild-type (WT) littermate controls (6-8 weeks old, 18-22 g) on a C57BL/6 background were supplied by Jiangsu GemPharmatech Biotechnology Co., Ltd. The SIRT7-KO model was generated via CRISPR/Cas9-mediated deletion of exons 4-9. Briefly, heterozygous founders were intercrossed to produce homozygotes. Genotypes were verified by PCR and Sanger sequencing (Table S2). Mice were anesthetized with tribromoethanol (30 μL/g, Meiunbio, #MA0478), and recombinant AAV6 vectors (Tsingke Biotechnology) encoding green fluorescent protein (GFP, control), FOXO4-WT, FOXO4-K139R (mimicking desuccinylation), or FOXO4-K139E (mimicking succinylation) were delivered by intratracheal instillation (1 × 10^11^ vg in 50 μL saline). After three weeks, following a 12-h fast, ALI was induced by intratracheal LPS (10 mg/kg, Sigma Aldrich, #L2880). Notably, a lethal dose of LPS (25 mg/kg) was employed in the survival study. Furthermore, TLB was administered intraperitoneally at a dose of 20 mg/kg (MedChemExpress, #HY-N4100) 2 h before LPS challenge [30]. Mice were euthanized 24 h post-LPS challenge for tissue collection. All mice were maintained under specific pathogen-free conditions with a 12-h light/dark cycle and ad libitum access to food and water. Animal procedures followed the NIH Guide for the Care and Use of Laboratory Animals and were approved by the Institutional Animal Care and Use Committee (IACUC) of Jinling Hospital, Medical School of Nanjing University (Approval No. DZGDWLS202503000244).

### 2.3 Collection of bronchoalveolar lavage fluid (BALF)

BALF was collected from ARDS patients and matched controls within 24 h post-admission using sterile 15 mL tubes. After filtration through a 50 μm strainer, samples were centrifuged (4 °C, 300×g, 10 min). The supernatant was aspirated and the pellet retained for analysis. For murine BALF, terminal anesthesia was administered followed by exsanguination. Ice-cold phosphate-buffered saline (PBS, 0.8 mL) was slowly instilled into the lungs via tracheal cannula and gently aspirated. This lavage cycle was repeated three times, yielding approximately 2 mL BALF per mouse. After centrifugation, supernatant was aliquoted and stored at −80 °C, and the pellet resuspended in 1 mL ice-cold PBS for processing.

### 2.4 Cell culture

This study utilized four cell lines: mouse alveolar epithelial (MLE-12), alveolar macrophage (MHS), pulmonary microvascular endothelial (PMVEC), and human embryonic kidney (HEK293T). MLE-12 and MHS were obtained from SUNNCELL Biotechnology Co., Ltd., while PMVEC and HEK293T were sourced from Shanghai Zhongqiao Xinzhou Biotechnology Co., Ltd. All cells were cultured at 37 °C in a 5% CO_2_ atmosphere. Culture conditions were as follows: MLE-12 in DMEM/F12 (Gibco, #11320033) with 10% fetal bovine serum (FBS, Gibco, #A5256701) and 1% penicillin-streptomycin (Beyotime, #C0222); MHS in RPMI 1640 (Gibco, #11875093) with identical FBS and antibiotic concentrations; HEK293T in high-glucose DMEM (Gibco, #11965092) with the same supplements; and PMVEC in a commercial medium specific for primary mouse pulmonary microvascular endothelial cells (Shanghai Zhongqiao Xinzhou Biotechnology Co., Ltd, #PCM-M-01). Cell lines were authenticated by short tandem repeat (STR) profiling prior to use, and mycoplasma testing was conducted routinely to exclude contamination.

### 2.5 Construction of SIRT7-KO cell lines

A stable SIRT7-KO MLE-12 cell line was established using CRISPR-Cas9-mediated genome editing for functional studies. The methodology involved four key steps: vector construction, selection optimization, lentiviral production, and cell line validation. Three sgRNAs targeting the murine SIRT7 gene were designed and synthesized, followed by construction of a CRISPR/Cas9 KO plasmid. Successful editing was verified through Sanger sequencing using the following primer pair: forward 5′-GAAATCCCGAAGGAGCAAGGG-3′ and reverse 5′-CCGAGTTTGCATCCCAGAAG-3′. The optimal puromycin selection concentration was set at 2 μg/mL by dose-response titration after verifying HEK293T clonogenicity. Lentiviral vectors and helper plasmids were co-transfected into HEK293T cells for recombinant lentivirus packaging. Three independent viral preparations were validated by titering and qPCR. Lentiviruses 2 and 3, harboring SIRT7-targeting sgRNAs, were sequentially co-infected into MLE-12 cells. Puromycin selection (2 μg/mL, 72-96 h) enriched stable transductants, establishing a polyclonal pool. Single-cell cloning via limiting dilution yielded individual clones. PCR and sequencing identified one clone with a 218-bp deletion causing frameshift and premature stop codon, confirming biallelic SIRT7-KO. sgRNA and control sequences are listed in Table S3.

### 2.6 shRNA knockdown (KD)

To establish stable KD models for FOXO4, MDM2, BTRC, UBE4B, STUB1, and UBE4A, murine gene-specific shRNA sequences were designed and synthesized by Tsingke Biotechnology (Beijing, China), cloned into a lentiviral vector, and packaged in HEK293T cells. Lentiviral particles transducing each target-specific or control shRNA were used to infect MLE-12 cells, followed by puromycin selection (2 μg/mL, 72 h) to generate stably transduced populations. KD efficiency for all targets was validated by quantitative Western blotting. shRNA sequences are detailed in Table S3.

### 2.7 Plasmid constructs and transfection

All plasmids utilized in this study were designed and constructed by Tsingke Biotechnology (Beijing, China). Clone verification and full-length Sanger sequencing were performed by the same vendor to confirm plasmid identity and structural integrity. Transient transfections were carried out using jetPRIME® DNA/siRNA Transfection Reagent (Polyplus, #101000046) in strict accordance with the manufacturer's instructions. Detailed descriptions of each plasmid, including backbone, insert information, restriction sites, and selection markers, are provided in Table S4.

### 2.8 RNA extraction, quantitative real-time PCR (qPCR) and RNA sequencing

Total RNA was isolated using TRIzol reagent (Thermo Scientific, USA), followed by DNase I digestion to remove genomic DNA. RNA purity was verified spectrophotometrically. cDNA synthesis was performed with the HiScript III Reverse Transcription Kit (Vazyme Biotech, #R302-01). qPCR was conducted using SYBR Green Premix Pro Taq HS (Accurate Biology, #AG11740) per manufacturer's protocols. Gene expression levels were calculated via the 2^−ΔΔCt^ method, normalized to β-actin. Primer sequences are listed in Table S5. RNA sequencing was carried out by Tsingke Biotechnology (Beijing, China). Differentially expressed genes (DEGs) were identified using the limma package in R (v4.3.1), with statistical significance defined as *p* < 0.05 and a minimum absolute log_2_ fold-change threshold of |log_2_(FC)| ≥ 0.5. Functional enrichment analyses were subsequently conducted using established bioinformatics tools and databases.

### 2.9 DNA gel electrophoresis

Genotyping of SIRT7-KO mice was conducted using PCR amplification followed by agarose gel electrophoresis. Briefly, genomic DNA was extracted from lung tissues of SIRT7-KO and WT mice post proteinase K digestion via phenol-chloroform method, with concentration and purity (A260/A280) quantified spectrophotometrically. PCR products electrophoresed on 1% agarose/GoldView™ gels (120 V, 30 min) were imaged digitally, and band clarity was enhanced by background inversion using ImageJ (NIH, USA). Primer sequences are listed in Table S2.

### 2.10 Flow cytometric analysis

To quantify the neutrophil-to-macrophage ratio in murine BALF, flow cytometry was performed. Mice were deeply anesthetized and euthanized, followed by tracheal intubation. The lungs were inflated with 1 mL of ice-cold complete RPMI 1640 medium via the airway, and BALF was collected after three rounds of bronchoalveolar lavage. Cells were pelleted by centrifugation (300 × g, 10 min, 4 °C), treated with 1× RBC lysis buffer, and resuspended in PBS containing 1% BSA. To block nonspecific binding, cells were incubated with anti-mouse CD16/CD32 antibody (BD Biosciences, #553141) on ice for 30 min. Subsequently, cells were stained with PE-conjugated anti-F4/80 (BioLegend, #123110) and APC-conjugated anti-Ly6G (BioLegend, #127614) for 30 min on ice in the dark. After washing, viability staining was performed using Fixable Viability Dye eFluor™ 780 (Thermo Scientific, #65-0865-14) for 30 min. Cells were washed again and resuspended in PBS with 1% BSA for immediate analysis on a BD FACSCalibur system. Data were acquired with CellQuest software and analyzed using FlowJo. Absolute neutrophil and macrophage counts in BALF were determined by multiplying the total BALF cell count by their respective differential percentages.

### 2.11 Western blotting and Co-immunoprecipitation (Co-IP)

Cell and tissue lysates were prepared using RIPA (Proteintech, #PR20036) or Co-IP lysis buffer (Proteintech, #PR20037), supplemented with protease (MedChemExpress, #HY-K0010) and deacetylase inhibitor cocktails (MedChemExpress, #HY-K0030) immediately prior to use. After centrifugation at 13,000 × g for 15 min at 4 °C, supernatants were collected and protein concentration was measured by BCA assay (Beyotime, #P0011). Equal protein amounts were denatured in 5× SDS-PAGE loading buffer at 100 °C for 5 min, separated by SDS-PAGE (Epizyme Biotech, #LK404), and transferred to PVDF membranes (Merck Millipore, #IPVH00010). Membranes were blocked with 5% non-fat milk in TBST, incubated with primary antibodies at 4 °C overnight, washed, and then probed with HRP-conjugated secondary antibodies. Signals were detected by ECL (ShareBio, #SB-WB004) and quantified using ImageJ (NIH, USA). For proteins of similar molecular weights, membranes were stripped (CWBIO, #CW0056 M) prior to sequential immunoblotting. Additionally, total protein staining with Ponceau S (Beyotime, #P0022) was performed on all lanes immunoblotted for pan-succinylation to confirm the uniformity of sample loading. For Co-IP assays, pre-cleared lysates were incubated with target antibodies overnight at 4 °C with rotation, followed by incubation with Protein A/G agarose beads (Beyotime, #P2108) for 3 h. Immune complexes were washed three times with ice-cold IP lysis buffer and analyzed by Western blotting. Antibody details are provided in Table S6. Of note, although the theoretical molecular weight of FOXO4 is ∼57 kDa, it is routinely detected as an ∼65 kDa band on SDS-PAGE due to its high proline content and PTMs.

### 2.12 Immunoprecipitation and mass spectrometry (IP-MS) analysis

MLE-12 cells were lysis in Co-IP buffer (Proteintech, #PR20037) containing protease inhibitor cocktail (MedChemExpress, #HY-K0010) and deacetylase inhibitor (MedChemExpress, #HY-K0030). The lysate was centrifuged at 13,000 × g for 15 min at 4 °C to remove insoluble debris. Protein concentration in the clarified supernatant was determined using the BCA assay (Beyotime, #P0011). Equal amounts of protein were immunoprecipitated overnight at 4 °C with Protein A/G agarose beads conjugated to anti-SIRT7 antibody. Following immunoprecipitation, complexes were washed thrice with ice-cold buffer, resolved by Western blotting, and visualized with Coomassie Blue (Beyotime, #P0003S). Target protein bands were excised, subjected to in-gel tryptic digestion, and analyzed by LC-MS/MS (Q Exactive™ Plus, Thermo Scientific) at PTM Bio (Hangzhou, China).

### 2.13 Chromatin immunoprecipitation assay (ChIP)

ChIP was performed using a commercial kit (Beyotime, #P2083S). MLE-12 cells were cross-linked with 1% formaldehyde (37 °C, 10 min), followed by nuclear extraction with the chromatin extraction kit (Abcam, #ab117152) per manufacturer's protocol. Chromatin was sonicated to 200-600 bp fragments, then purified through centrifugation to obtain soluble chromatin. Aliquot chromatin samples were incubated overnight at 4 °C with anti-FOXO4 antibody (experimental) or isotype-matched IgG (control). Immune complexes were isolated using protein A/G magnetic beads, eluted, and purified. FOXO4 binding to target genomic regions was quantified by qPCR; primer sequences are provided in Table S7.

### 2.14 Dual-luciferase reporter assay

Experiments were performed using a Dual-Luciferase Reporter Assay Kit (Beyotime, #RG029S) according to the manufacturer's protocol. The experimental design comprised ten groups: (1) empty vector control (pcDNA3.1 + pGL3-basic) to establish baseline luminescence; (2) FOXO4 single expression control (FOXO4 + pGL3-basic) to exclude nonspecific reporter activation; (3) GPX4 promoter WT baseline (pcDNA3.1 + GPX4 promoter-WT) to determine intrinsic transcriptional activity; (4) FOXO4 with GPX4 promoter WT (FOXO4 + GPX4 promoter-WT) to evaluate FOXO4 mediated transactivation; (5) GPX4 promoter MUT1 baseline (pcDNA3.1 + GPX4 promoter-MUT1); (6) FOXO4 with GPX4 promoter MUT1 (FOXO4 + GPX4 promoter-MUT1); similarly for MUT2 (groups 7 and 8) and MUT3 (groups 9 and 10) to assess the effect of each mutation on basal and FOXO4 dependent transcription. Firefly and Renilla luciferase substrates were prepared fresh. At 48 h post transfection, MLE-12 cells were washed twice with ice cold PBS after aspiration of the culture medium. Then, 500 μL of lysis buffer was added per well and incubated at room temperature for 20 min with gentle shaking. Lysates were transferred to prechilled microcentrifuge tubes and centrifuged at 12,000 × g for 5 min at 4 °C. The supernatant was collected, and 20 μL was aliquoted into a black 96 well plate. Firefly luciferase activity was measured by adding 100 μL of assay reagent, followed by immediate luminescence recording (F). Renilla luciferase activity was then measured after adding 100 μL of reagent and incubating for 10 min at room temperature (R). Reporter gene expression was quantified as the normalized F/R ratio.

### 2.15 In vitro SIRT7 desuccinylase activity assay

To assess the direct effect of TLB on the desuccinylase activity of SIRT7, recombinant human SIRT7 (Abcam, #ab104032) and a synthetic H3K122suc-AMC fluorescent peptide (sequence: RVTIMPKsuc-AMC; QYAOBIO) were employed. This substrate is well validated as a specific probe for monitoring SIRT7 desuccinylase activity, as multiple independent studies have established that SIRT7 directly desuccinylates H3K122 in vitro, a modification critical for chromatin compaction, genomic stability, and DNA damage repair [[31], [32], [33]]. Each reaction (20 μL) was conducted in a buffer containing 50 mM Tris-HCl (pH 8.0), 137 mM NaCl, 2.7 mM KCl, and 1 mM MgCl_2_, supplemented with 1 mM NAD^+^, 5 μM peptide substrate, 0.5 μg SIRT7, and TLB at concentrations ranging from 0.1 to 100 μM (0 μM DMSO vehicle as control; final DMSO concentration: 0.1% v/v). A no-enzyme control (NC) was included to correct for spontaneous hydrolysis of the substrate, and a buffer-only blank was used for instrument background subtraction. After incubation at 37 °C for 30 min, reactions were terminated by adding 10 μL of 10% trifluoroacetic acid (TFA). Fluorescence intensity was measured at excitation/emission wavelengths of 360/460 nm using a microplate reader (Synergy HT, BioTek). Net relative fluorescence units (RFU) were calculated by subtracting the NC signal from each experimental group, and data were normalized to the vehicle control (0 μM, set as 100%). Dose response curve fitting was performed using a four parameter logistic (4 PL) nonlinear regression model to derive the half maximal effective concentration (EC_50_) and Hill slope.

### 2.16 Immunofluorescence (IF)

Mouse lung paraffin sections underwent deparaffinization and rehydration through a xylene-ethanol series, followed by heat-mediated antigen retrieval in citrate buffer. After three 5-min PBS washes, samples were processed concurrently with cell preparations. MLE-12 and HEK293T cells, cultured on sterile coverslips, were fixed in 4% PFA for 15 min at room temperature and washed similarly. All samples were permeabilized with 0.2% Triton X-100 (10 min), blocked with 5% BSA (1 h), and incubated with primary antibodies at 4 °C overnight. Following PBS washes, samples were incubated with species-matched fluorescent secondary antibodies for 1 h in the dark, counterstained with DAPI, and mounted using anti-fade medium. Imaging was performed on a Zeiss LSM 800 confocal microscope (Carl Zeiss AG, Germany). Antibody details are provided in Table S6.

### 2.17 Histopathological analysis

Lung specimens were fixed in 4% paraformaldehyde for 24 h at room temperature, then subjected to graded ethanol dehydration, xylene clearing, and paraffin embedding. Serial 4-μm sections were stained with hematoxylin and eosin (H&E) for light-microscopic examination by two blinded, independent investigators trained in pulmonary pathology. ALI severity was evaluated using an established semiquantitative scoring system assessing four parameters: alveolar edema, intra-alveolar hemorrhage, inflammatory cell infiltration, and alveolar wall thickening. Each parameter was graded on a scale of 0-3 (0 = absent, 1 = mild, 2 = moderate, 3 = severe). The final score for each parameter represented the mean of two independent assessments, with total scores (range 0-12) calculated as the sum of all parameters, ensuring objective and reproducible evaluation.

### 2.18 Kaplan-Meier survival analysis

Following intratracheal administration of a lethal dose of LPS (25 mg/kg) in WT and SIRT7-KO mice, survival was monitored for 72 h. Survival status was dichotomized as survivors (alive at the 72-h endpoint) or non-survivors (death occurring during observation). Kaplan-Meier curves were generated, and between-group differences in survival distribution were assessed using the log-rank test, with statistical significance defined as *p* < 0.05.

### 2.19 Detection of inflammatory cytokines

The quantification of inflammatory cytokines including interleukin-6 (IL-6), tumor necrosis factor-α (TNF-α), and interleukin-1β (IL-1β) was performed using commercially available enzyme-linked immunosorbent assay (ELISA) kits in strict accordance with the manufacturer's protocols. The following kits were utilized: Mouse IL-6 ELISA Kit (MULTI SCIENCES, #EK206), Mouse TNF-α ELISA Kit (MULTI SCIENCES, #EK282), and Mouse IL-1β High Sensitivity ELISA Kit (MULTI SCIENCES, #EK201BHS).

### 2.20 Lung wet-to-dry (W/D) weight ratio

Upon euthanasia, murine lung tissues were promptly excised, gently blotted to remove superficial fluid, and precisely weighed to record wet mass. The specimens were subsequently dehydrated in a forced-air oven at 65 °C for 48 h until a stable mass was attained. After cooling to ambient temperature within a desiccator, the tissues were reweighed to determine dry mass. The pulmonary W/D weight ratio was computed as the quotient of wet mass to dry mass.

### 2.21 Cell viability and cytotoxicity

Cell viability was determined using the Cell Counting Kit-8 (CCK-8, MedChemExpress, #HY-K0301) according to standardized protocols. MLE-12 cells were plated in 96-well plates at a density of 1 × 10^4^ cells/well and maintained at 37 °C in a humidified 5% CO_2_ atmosphere for 24 h to achieve confluent adherence. Subsequently, cells were assigned to experimental groups and pretreated for 30 min with Fer-1 (10 μM, MedChemExpress, #HY-100579), RSL3 (2 μM, MedChemExpress, # HY-100218A), Z-VAD-FMK (20 μM, MedChemExpress, #HY-16658B), or MCC950 (10 μM, MedChemExpress, #HY-12815), followed by a 24-h LPS (10 μg/mL) challenge. Following this treatment interval, 20 μL of CCK-8 solution was administered to each well, followed by a 4 h incubation period at 37 °C. Absorbance measurements were conducted at 450 nm using a microplate reader. The percentage of cell viability was computed using the following equation: [(OD_450_ experimental - OD_450_ blank)/(OD_450_ negative control - OD_450_ blank)] × 100%. Cytotoxicity was assessed using the LDH release assay (Abcam, #ab65393). In brief, after cell pretreatment, 10 μL of supernatant and 100 μL of LDH reagent were added per well, incubated for 30 min at room temperature, and absorbance was measured at 450 nm.

### 2.22 Protein stability assay

Cells were treated with cycloheximide (CHX, MedChemExpress, #HY-12320) at 50 μg/mL in complete culture medium and collected at specified intervals (0, 0.5, 1, 1.5, and 2 h). Protein extraction was performed using ice cold RIPA buffer with protease inhibitors. Target protein expression and degradation kinetics were analyzed by Western blotting.

### 2.23 Detection of Fe^2+^, MDA, SOD, GSH, 4-HNE and ROS

Quantitative analysis of ferrous iron (Fe^2+^), malondialdehyde (MDA), superoxide dismutase (SOD), reduced glutathione (GSH), and 4-hydroxynonenal (4-HNE) was performed using standardized protocols. Freshly harvested mouse lung tissue (10 mg) or MLE-12 cells (1.5 × 10^6^ cells/mL) were homogenized in ice-cold lysis buffer on ice and centrifuged (8000 × g, 10 min, 4 °C). Supernatants were collected for analyte detection. Total protein was quantified using a BCA assay (Beyotime, #P0011), and all measurements were normalized to protein content. Fe^2+^ was measured using colorimetric kits (Elabscience, #E-BC-K881-M and #E-BC-K773-M); MDA (Beyotime, #S0131S), SOD (Beyotime, #S0101S), and GSH (Beyotime, #S0053) were analyzed per manufacturer's instructions. For 4-HNE detection, BALF and MLE-12 cell culture supernatants were centrifuged (5000 × g, 10 min, 4 °C) and assessed by ELISA (ELK Biotechnology, ELK8374). To detect total ROS, frozen lung sections (10 μm) were stained with 10 μM DHE (MedChemExpress, #PD-MY-003) for 30 min. Cells were pre-stained with 5 μM DCFH-DA (Beyotime, #S0035 M) in culture medium at 37 °C for 30 min. To detect lipid ROS, cells were pre-incubated with 5 μM C11-BODIPY 581/591 (MedChemExpress, #HY-D1301) at 37 °C for 30 min and then imaged using a Zeiss LSM 800 confocal microscope (Carl Zeiss AG, Germany). Fluorescence intensity was quantified using ImageJ (NIH, USA).

### 2.24 Transmission electron microscopy (TEM)

Mouse lung tissues were dissected into uniform 1 mm^3^ fragments and processed concurrently with MLE-12 cells. After primary fixation in 4% glutaraldehyde (4 °C, 4 h) (Servicebio, #G1102), samples were rinsed three times with 0.1 M PBS and post-fixed in 1% aqueous osmium tetroxide for 2 h. Dehydration through a graded ethanol series (30-100% v/v), propylene oxide infiltration, and embedding in Epon 812 epoxy resin were followed by polymerization at 60 °C for 48 h. Ultrathin sections (70 nm) were stained with uranyl acetate and lead citrate and examined by TEM using a Hitachi HT7800 microscope. For blind quantification of ferroptosis-positive cells based on mitochondrial morphology, TEM samples were anonymized by an independent researcher prior to analysis. Two investigators, blinded to experimental group assignment, assessed mitochondrial morphology using established ultrastructural criteria for ferroptosis [[34], [35], [36]]. A cell was classified as ferroptosis-positive only if its mitochondria exhibited shrinkage, increased electron density of the mitochondrial membranes, and disassembly or loss of cristae. Ambiguous or borderline cases were conservatively scored as negative to ensure analytical stringency. For each experimental condition, three independent biological replicates were analyzed; from each replicate, 10 randomly selected non-overlapping fields were imaged at 6000× magnification. Representative high-magnification insets (15,000×) were additionally acquired for validation.

### 2.25 Nuclear-cytoplasmic extraction

Nuclear and cytoplasmic proteins were isolated using a commercial extraction kit (Thermo Scientific, #78833) in strict accordance with the manufacturer's protocol to ensure subcellular fraction purity.

### 2.26 Molecular docking

The crystal structures of murine SIRT7 and FOXO4 were sourced from the AlphaFold Protein Structure Database (https://alphafold.com/). The three-dimensional conformation of TLB was acquired from the PubChem Compound Database (https://pubchem.ncbi.nlm.nih.gov/). Molecular docking simulations employed two distinct methodological frameworks: protein-protein interactions between SIRT7 and FOXO4 were analyzed using the HDOCK web server (http://hdock.phys.hust.edu.cn/), while the binding interface of SIRT7 with TLB was characterized through AutoDock Vina. Prior to docking procedures, all protein structures underwent systematic preprocessing via PyMOL 2.4 to ensure structural integrity and physiological relevance. This included removal of crystallization artifacts (water molecules and non-covalently bound heteroatoms), hydrogen atom addition, and protonation state optimization at pH 7.4. Docking conformations were rigorously evaluated using a tripartite assessment system: HDOCK score (quantifying binding affinity), confidence score (predictive reliability index), and ligand root-mean-square deviation (conformational stability metric). The optimal docking complex was selected based on synergistic consideration of computational scores and structural bioinformatics criteria, with particular emphasis on binding pocket complementarity and interaction thermodynamics. All molecular visualizations and structural analyses were generated using PyMOL 2.4.

### 2.27 Statistical analysis

Statistical analyses were performed using GraphPad Prism 9.5 (San Diego, CA). Continuous data are expressed as mean ± SD for normally distributed variables or median [IQR] for non-normal distributions, with normality assessed by Shapiro-Wilk test. Group comparisons were performed using the unpaired *t*-test or the Mann-Whitney *U* test (two groups); one-way ANOVA with Tukey's test or Kruskal-Wallis with Dunn's test (multiple groups); and two-way ANOVA with Tukey's test. Survival analysis employed Kaplan-Meier curves with log-rank testing. Correlations between SIRT7 expression and clinical parameters in ARDS patients were analyzed using Spearman tests. Significance was defined as *p* < 0.05 (two-tailed), denoted as *p < 0.05, **p < 0.01, ***p < 0.001.

## 3 Results

### 3.1 SIRT7 mediates protein pan-succinylation in ALI

**Fig. 1:**
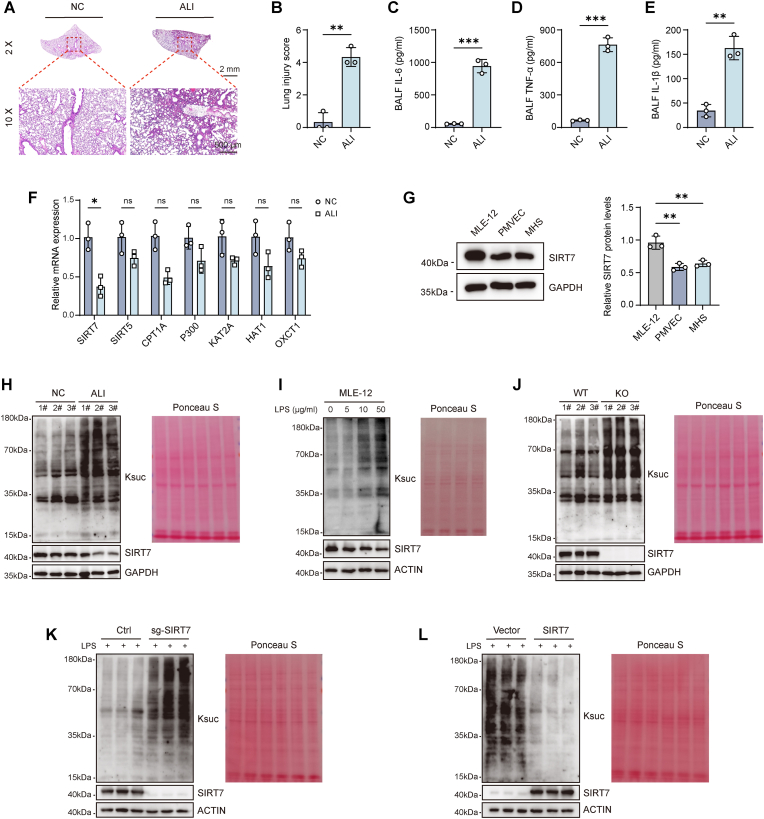
SIRT7 mediates protein Pan-Succinylation i**n ALI. A-B** Representative H&E-stained lung sections and quantitative lung injury scores from normal control (NC) and ALI model mice (n = 3, up: scale bar = 2 mm; down: scale bar = 500 μm). **C-E** BALF levels of inflammatory mediators (IL-6, TNF-α and IL-1β) were compared between NC and ALI model mice (n = 3). Student's t-test. **F** qPCR analysis of desuccinylase and succinyltransferase mRNA expression in lung tissues from NC and ALI model mice (n = 3). Student's t-test was used for pairwise comparisons, and Bonferroni correction was applied for multiple testing. **G** SIRT7 protein expression levels in MLE-12, PMVEC, and MHS cells were analyzed by Western blotting (n = 3). One-way ANOVA followed by Tukey's post-hoc test for multiple comparisons. **H** Western blotting was employed to evaluate SIRT7 protein expression and pan-succinylation levels in lung tissues derived from NC and ALI model mice (n = 3). Ponceau S staining confirmed equal loading. **I** Western blotting was performed to assess LPS concentration-dependent alterations (0, 5, 10, 50 μg/mL) in SIRT7 protein expression and pan-succinylation levels in MLE-12 cells (n = 3). Ponceau S staining confirmed equal loading. The immunoblot shown is representative of three independent biological replicates. **J** Western blotting was employed to determine pan-succinylation levels in lung tissues from WT and SIRT7-KO mice subjected to ALI (n = 3). Ponceau S staining confirmed equal loading. **K-L** Western blotting was used to quantify lysine pan-succinylation levels in MLE-12 cells following SIRT7-KO or OE (n = 3). Ponceau S staining confirmed equal loading. Data are presented as mean ± SD. Each experiment was conducted with three independent biological replicates. *p < 0.05, **p < 0.01, and ***p < 0.001; ns, not significant.

To investigate alterations in pan-succinylation during ALI, an experimental model was established using LPS-induced ALI in mice. Histological evaluation via H&E staining revealed intact alveolar architecture in control animals, whereas lung tissues from ALI mice exhibited marked pathological changes, including alveolar wall thickening, substantial neutrophil infiltration, proteinaceous exudate, and perivascular erythrocyte extravasation (Fig. 1A). Consistent with these morphological alterations, lung injury scores and levels of pro-inflammatory cytokines (IL-6, TNF-α, and IL-1β) in BALF were significantly elevated in the ALI group compared with controls (Fig. 1B–E). Western blotting further indicated a pronounced increase in pan-succinylation in lung tissues from ALI mice (Fig. 1H). Given that succinylation is dynamically regulated by enzymatic activity, qPCR was performed to assess mRNA expression of key succinyltransferases (CPT1A, P300, KAT2A, HAT1, OXCT1) and desuccinylases (SIRT5, SIRT7) in lung tissues. Expression of all enzymes examined was downregulated in ALI samples, with the most notable reduction observed for SIRT7 (Fig. 1F). This decrease was confirmed at the protein level by Western blotting (Fig. 1H), suggesting that SIRT7 may function as a principal desuccinylase modulating succinylation in ALI. Based on the central role of AECs, macrophages, and endothelial cells in ALI pathogenesis, SIRT7 protein expression was analyzed across relevant cell lines (MLE-12, MHS, PMVEC). Highest SIRT7 expression was detected in MLE-12 cells (Fig. 1G). Immunofluorescence staining of mouse lung sections demonstrated widespread SIRT7 expression, with notable co-localization with the AEC marker surfactant protein C (SP–C) (Fig. S1A), supporting a potential role for SIRT7 in regulating succinylation within alveolar epithelial compartments during ALI.

To elucidate the specific role of SIRT7 in ALI, SIRT7-KO mice were generated using CRISPR-Cas9. In the ALI model, SIRT7 deficiency markedly elevated pan-succinylation levels in lung tissue (Fig. 1J). In vitro, LPS stimulation increased pan-succinylation in MLE-12 cells in a dose-dependent manner, which was accompanied by reduced SIRT7 protein expression (Fig. 1I). In light of the phenotypic response observed herein and in alignment with prior literature, a concentration of 10 μg/mL LPS was employed for subsequent cellular stimulation experiments [7]. Consistent with these findings, genetic ablation of SIRT7 augmented succinylation in MLE-12 cells, whereas SIRT7 overexpression (SIRT7-OE) suppressed it (Fig. 1K–L). Collectively, these data indicate that SIRT7 may contribute to ALI pathogenesis by regulating succinylation of specific target proteins.

### 3.2 SIRT7 deficiency exacerbates LPS-induced ALI

**Fig. 2:**
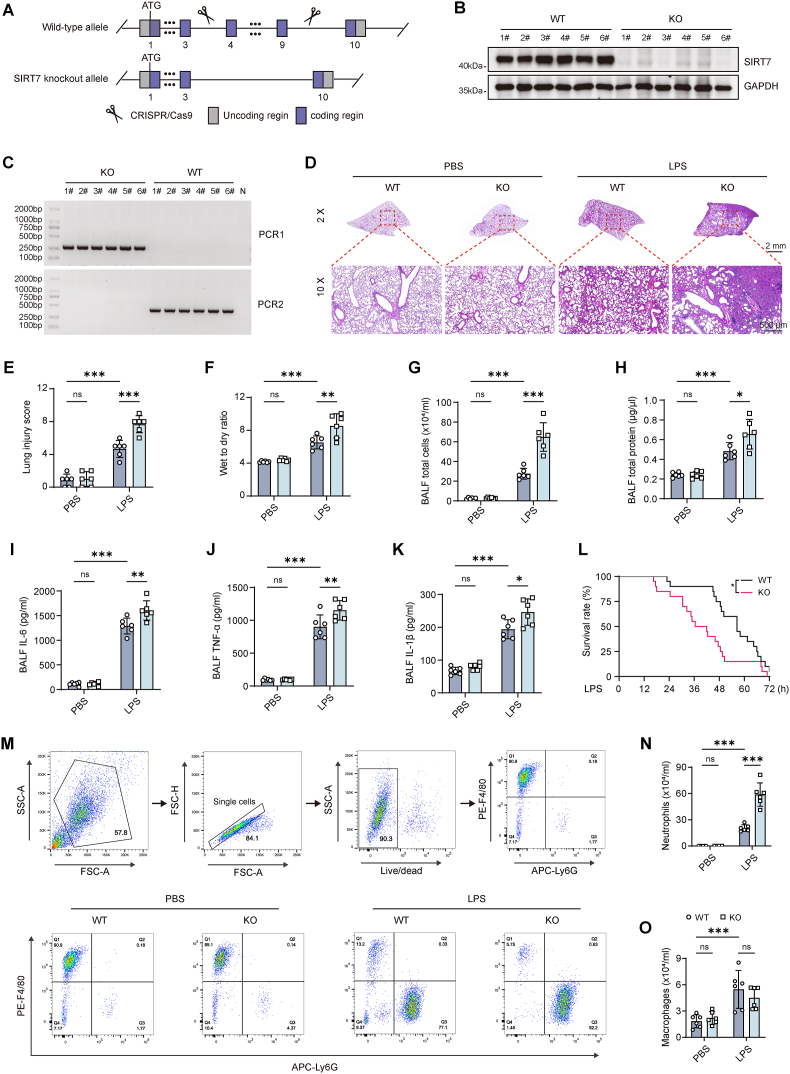
SIRT7 deficiency exacerbates LPS-triggered ALI in **vivo. A-B** Diagram of the SIRT7-KO strategy and quantitative assessment of SIRT7 protein expression in lung tissues from WT and SIRT7-KO mice (n = 6). **C** Genomic DNA from WT and SIRT7-KO mice was analyzed by agarose gel electrophoresis to confirm their respective genotypes (n = 6). N, no-template control. **D-E** Representative H&E-stained lung tissue sections and quantitative lung injury scores from WT and SIRT7-KO mice following 24 h LPS challenge (n = 6, up: scale bar = 2 mm; down: scale bar = 500 μm). Two-way ANOVA followed by Tukey's post-hoc test for multiple comparisons. **F–K** Comparison of lung W/D weight ratio and BALF parameters, including total cells, total protein, and pro-inflammatory cytokines (IL-6, TNF-α and IL-1β) levels, in LPS-challenged ALI between WT and SIRT7-KO mice (n = 6). Two-way ANOVA followed by Tukey's post-hoc test for multiple comparisons. **L** Survival curves of WT and SIRT7-KO mice following intratracheal instillation of a lethal dose of LPS (25 mg/kg) (n = 20). Log-rank test. **M** Flow cytometry gating strategy for identification of target immune cell populations in murine BALF. Single-cell suspensions were prepared from BALF samples, followed by staining with a fixable viability dye to exclude nonviable cells. Neutrophils and macrophages were identified by sequential gating on FSC/SSC and lineage-defining surface markers. Flow cytometry was performed to quantify the proportions of neutrophils and macrophages in BALF from WT and SIRT7-KO mice with LPS-challenged ALI (n = 6). Neutrophils and macrophages were identified as Ly6G^+^/F4/80^-^ and Ly6G^−^/F4/80^+^ cells, respectively. Representative dot plots are shown. **N–O** Absolute counts of neutrophils and macrophages in BALF collected from mice across all experimental groups presented in (M) (n = 6). Two-way ANOVA followed by Tukey's post-hoc test for multiple comparisons. Data are presented as mean ± SD. Each experiment was conducted with six independent biological replicates. *p < 0.05, **p < 0.01, and ***p < 0.001; ns, not significant.

To delineate the regulatory role of SIRT7 in ALI pathogenesis, we engineered a global SIRT7-KO murine model. SIRT7 gene deletion was confirmed by agarose gel electrophoresis analysis of tail-derived genomic DNA (Fig. 2A and C), and Western blotting demonstrated complete absence of SIRT7 protein in lung homogenates from homozygous mutants (Fig. 2B). Following intratracheal LPS challenge, SIRT7-KO mice exhibited exacerbated pulmonary injury at 24 h compared with WT controls. Histopathological evaluation revealed comparable tissue architecture between unchallenged genotypes. Moreover, LPS-induced ALI manifestations, including neutrophilic infiltration, alveolar wall thickening, hemorrhage, edema, and structural disruption, were markedly amplified in SIRT7-KO mice (Fig. 2D). Concordantly, histopathological injury scores and pulmonary W/D weight ratios were significantly elevated in KO animals (Fig. 2E–F). BALF analysis showed increased total cell counts, protein concentration, and proinflammatory cytokine levels (IL-6, TNF-α, and IL-1β) in SIRT7-KO mice (Fig. 2G–K). Furthermore, SIRT7-KO significantly reduced survival following LPS challenge (Fig. 2L). Flow cytometry confirmed heightened neutrophil recruitment in BALF from SIRT7-KO mice without altering macrophage populations (Fig. 2M–O). In vitro, SIRT7-KO potentiated pro-inflammatory cytokines expression in LPS-stimulated MLE-12 cells (Fig. S1B–D), whereas SIRT7-OE suppressed it (Fig. S1E–G). These data establish that SIRT7 deficiency significantly exacerbates LPS-induced ALI in vivo and in vitro.

### 3.3 SIRT7 interacts with FOXO4

**Fig. 3:**
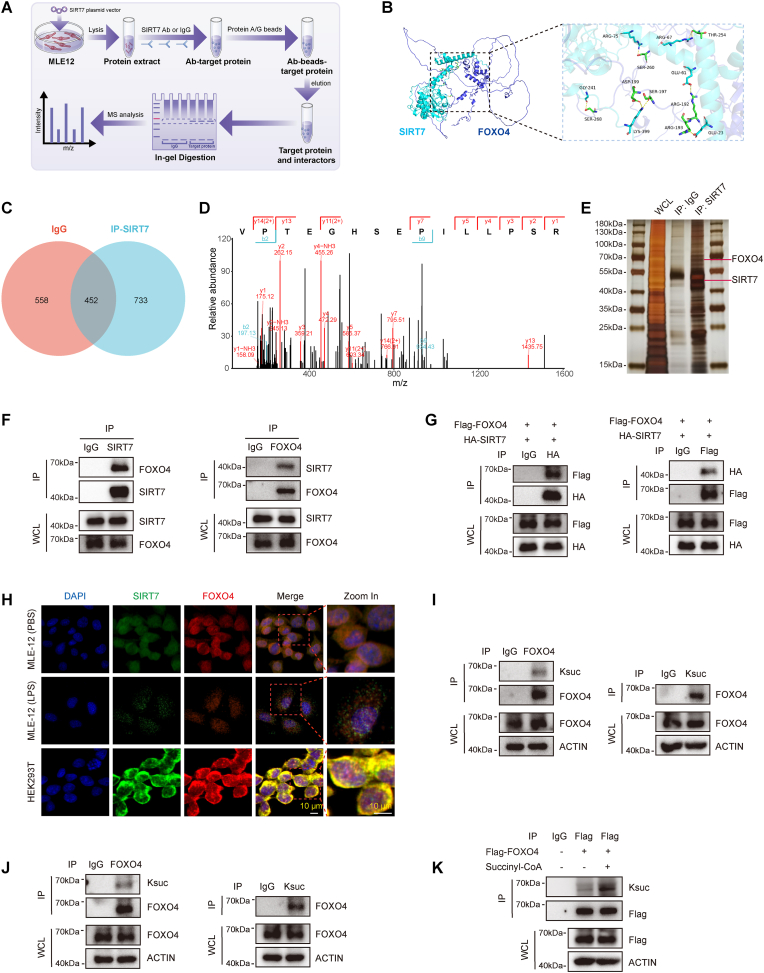
SIRT7 interacts with FOXO4. A The schematic diagram of IP-MS. **B** Molecular docking analysis of the interaction between SIRT7 and FOXO4. **C** Venn diagram illustrating the overlap of proteins identified by mass spectrometry from the IgG-enriched and SIRT7 immunoprecipitation samples. **D** FOXO4 is identified as a highly abundant protein in anti-SIRT7 immunoprecipitates by MS. **E** Silver staining of proteins from anti-SIRT7 immunoprecipitates. **F** Co-IP analysis of the endogenous FOXO4-SIRT7 interaction in MLE-12 cell lysates. **G** Co-IP analysis of the interaction between HA-tagged SIRT7 and Flag-tagged FOXO4 in co-transfected HEK293T cells. **H** Confocal microscopy analysis of SIRT7 (green) and FOXO4 (red) subcellular localization in MLE-12 cells following PBS or LPS treatment (upper and middle panels); and of HA-tagged SIRT7 (green) and Flag-tagged FOXO4 (red) in transfected HEK293T cells (lower panel). scale bar = 10 μm. LPS was applied at a concentration of 10 μg/mL for 24 h. **I** Co-IP analysis of endogenous total FOXO4 and succinylated FOXO4 in MLE-12 cells using the indicated antibodies. **J** Co-IP analysis of endogenous total FOXO4 and succinylated FOXO4 in HEK293T cells using the indicated antibodies. **K** In vitro succinylation assay of Flag-FOXO4 immunoprecipitated from HEK293T cells. The immunoprecipitates were incubated with 2 mM succinyl-CoA for 30 min and analyzed for FOXO4 succinylation. The data shown are representative of at least three independent biological experiments.

To delineate the molecular basis of SIRT7-mediated protection in ALI, we performed IP-MS to identify SIRT7-interacting proteins (Fig. 3A). After subtracting proteins detected in IgG controls, the ten most abundant proteins were ranked and listed (Fig. 3C–Table S8). Among these, FOXO4 attracted our attention due to its well-established role in modulating oxidative stress and inflammatory signaling pathways, as extensively documented in the literature [21,23,24,37]. Molecular docking demonstrates a thermodynamically favorable interaction between FOXO4 and SIRT7, with a predicted binding pose exhibiting a strongly negative binding free energy (Docking score = −292.46) and high structural complementarity (Fig. 3B). These computational results indicate the formation of a stable and specific protein-protein complex, suggesting a direct physical association that may regulate downstream cellular processes. Silver staining of SIRT7 immunoprecipitates revealed specific protein bands absent in IgG controls, including a ∼65 kDa band identified as FOXO4 by MS (Fig. 3D–E). Endogenous Co-IP in MLE-12 cells confirmed a direct SIRT7-FOXO4 interaction (Fig. 3F). Exogenous Co-IP in HEK293T cells expressing HA-tagged SIRT7 and FLAG-tagged FOXO4 also affirmed binding specificity (Fig. 3G), which was further supported by pronounced subcellular co-localization observed via immunofluorescence. Notably, under basal conditions, FOXO4 is predominantly nuclear and colocalizes with SIRT7, which retains it in the nucleus. LPS treatment rapidly induces FOXO4 cytoplasmic translocation and concurrently downregulates SIRT7 (Fig. 3H). Given the paucity of data on FOXO4 succinylation despite its regulation by diverse PTMs, we examined the succinylation status of endogenous FOXO4. Immunoprecipitation with anti-FOXO4 antibody followed by immunoblotting with anti-succinyl-lysine antibody detected a ∼65 kDa succinylation signal (Fig. 3I–J). Reverse pull-down corroborated this finding by enriching succinylated proteins using anti-succinyl-lysine antibody and probing with anti-FOXO4 antibody (Fig. 3I–J). In addition, incubation of recombinant FLAG-FOXO4 with succinyl-CoA induced dose-dependent succinylation (Fig. 3K). Together, these results demonstrate a physical interaction between SIRT7 and FOXO4 and indicate that FOXO4 is susceptible to succinylation, suggesting a potential regulatory role for SIRT7.

### 3.4 SIRT7 desuccinylates FOXO4 at K139 to inhibit its MDM2-mediated K48-linked ubiquitination and degradation

**Fig. 4:**
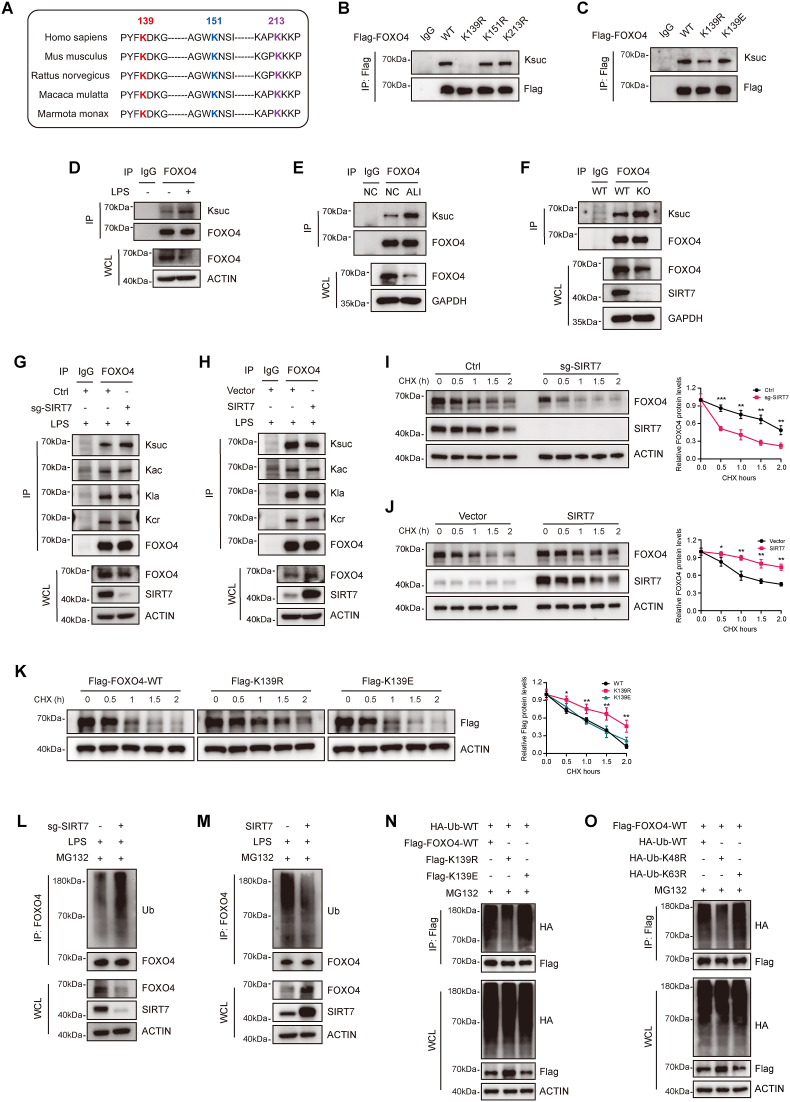
SIRT7 desuccinylated FOXO4 at K139 to inhibit its K48-linked ubiquitination degr**adation. A** The lysine residues at positions 139, 151, and 213 exhibit high evolutionary conservation across multiple species. **B** FOXO4 succinylation by anti-Flag immunoprecipitation in MLE-12 cells transfected with Flag-tagged FOXO4-WT or the K139R/K151R/K213R mutant, with IgG as the negative control. **C** FOXO4 succinylation by anti-Flag immunoprecipitation in MLE-12 cells transfected with the indicated constructs (Flag-tagged FOXO4 WT, K139R, and K139E), with IgG serving as the negative control. **D** Assessment of LPS stimulation on FOXO4 succinylation by Co-IP in MLE-12 cells. **E** FOXO4 succinylation by Co-IP in lung tissues from normal control (NC) and ALI mice. **F** FOXO4 succinylation in lung tissues from WT and SIRT7-KO mice subjected to ALI by Co-IP. **G** FOXO4 lysine succinylation (Ksuc), acetylation (Kac), lactylation (Kla) and crotonylation (Kcr) by Co-IP in MLE-12 cells from control (Ctrl) and sg-SIRT7 groups. MLE-12 cells from both groups were pretreated with LPS (10 μg/mL) for 24 h. **H** FOXO4 lysine succinylation (Ksuc), acetylation (Kac), lactylation (Kla) and crotonylation (Kcr) by Co-IP in MLE-12 cells pretreated with control (vector) and SIRT7-OE plasmids. MLE-12 cells were transfected with vector or SIRT7-OE plasmids and subsequently treated with LPS (10 μg/mL) for 24 h after 48 h of transfection. **I-J** SIRT7 was knocked out or overexpressed in MLE-12 cells, and FOXO4 protein expression was assessed by Western blotting following CHX (50 μg/mL) treatment for 0, 0.5, 1, 1.5, and 2 h (n = 3). Student's t-test. **K** Detection of FOXO4 protein levels in MLE-12 cells transfected with Flag-tagged FOXO4 WT, K139R, or K139E expressing plasmids following treatment with CHX (50 μg/mL) for the indicated times (0, 0.5, 1, 1.5, and 2 h) (n = 3). One-way ANOVA followed by Tukey's post-hoc test for multiple comparisons. **L-M** FOXO4 ubiquitination by Co-IP in MLE-12 cells with SIRT7-KO or OE, following pretreatment with LPS (10 μg/mL, 24 h) and MG132 (20 μM, 8 h). **N–O** FOXO4 ubiquitination by Co-IP in MLE-12 cells upon transfection with indicated plasmids and subsequent treatment with MG132 (20 μM for 8 h). Data are presented as mean ± SD. The data shown are representative of at least three independent biological experiments. *p < 0.05, **p < 0.01, ***p < 0.001.

Succinylation represents a prevalent PTM predominantly occurring at lysine (K) residues, characterized by its enrichment in regions of electrostatic complementarity on protein surfaces. To systematically identify putative succinylation sites within the FOXO4 protein, we utilized the GPSuc computational prediction platform (http://kurata14.bio.kyutech.ac.jp/GPSuc/index.php). The screening revealed three lysine residues (K139, K151, and K213) as high-probability modification sites (Table S9). Evolutionary conservation assessment via UniProt revealed notable conservation of these residues across vertebrate species, supporting their potential functional significance (Fig. 4A). To experimentally validate these predictions, we generated lysine to arginine (R) mutants to mimic desuccinylated states. Immunoblotting showed that K139R exhibited significantly reduced succinylation compared to K151R and K213R (Fig. 4B). In contrast, substitution of 139 lysine residues with glutamic acid (E, mimicking lysine succinylation) displayed substantially increased succinylation relative to K139R (Fig. 4C). These findings establish K139 as a principal succinylation site in FOXO4, with evolutionary conservation supporting its functional importance in regulatory mechanisms.

To delineate the regulatory function of FOXO4 succinylation in ALI, we initially quantified FOXO4 succinylation in MLE-12 cells via Co-IP. Notably, 24-h LPS stimulation induced a pronounced augmentation of FOXO4 succinylation (Fig. 4D). Concordantly, in vivo analyses of lung tissues from LPS-induced ALI models confirmed elevated FOXO4 succinylation (Fig. 4E). We next determined whether the desuccinylase SIRT7 modulates this modification. Functional assessments in vitro demonstrated that SIRT7-KO enhanced FOXO4 succinylation, whereas SIRT7-OE suppressed it. Crucially, SIRT7 manipulation did not influence other lysine modifications (e.g., acetylation, lactoylation, or crotonylation) of FOXO4, indicating exclusive specificity for succinylation (Fig. 4G–H). Consistent with these findings, SIRT7-KO mice exhibited significantly heightened FOXO4 succinylation in lung tissues under ALI conditions compared to WT controls (Fig. 4F). These data definitively identify SIRT7 as a physiological desuccinylase that specifically regulates FOXO4 succinylation during ALI.

Next, to clarify the molecular mechanism underlying lysine succinylation-mediated regulation of FOXO4 protein stability, we performed CHX chase assays in MLE-12 cells. Genetic perturbation of SIRT7 revealed that its KO substantially shortened FOXO4 protein half-life, whereas SIRT7-OE prolonged it (Fig. 4I–J). Using site-directed mutagenesis, we generated FOXO4-WT, K139R, and K139E plasmids. Half-life analyses demonstrated that the K139R significantly stabilized FOXO4 (Fig. 4K), suggesting that succinylation at K139 impairs its stability. We performed endogenous ubiquitination assays to further elucidate the regulatory mechanisms governing FOXO4 stability. These assays revealed that SIRT7-KO enhanced FOXO4 ubiquitination, whereas SIRT7-OE attenuated this modification (Fig. 4L–M). Co-IP analyses demonstrated that FOXO4-K139E robustly promotes its ubiquitination, whereas FOXO4-K139R suppresses it (Fig. 4N). Prior studies have established that distinct ubiquitin chain topologies, particularly K48- and K63-linked polyubiquitination, mediate divergent functional outcomes: K48-linked chains predominantly target substrates for proteasomal degradation, whereas K63-linked chains typically regulate non-proteolytic processes, including signal transduction and protein complex assembly [36]. To determine the linkage specificity of FOXO4 ubiquitination in our study, we transfected MLE-12 cells with expression plasmids encoding WT ubiquitin or site-specific mutants (K48R or K63R). Quantitative assessment of FOXO4 ubiquitination showed that the K48R mutation significantly diminished ubiquitin conjugation to FOXO4, whereas the K63R mutation exerted no significant effect (Fig. 4O). These findings indicate that K139 succinylation selectively facilitates K48-linked polyubiquitination of FOXO4, thereby reducing its stability.

To identify the E3 ubiquitin ligase responsible for FOXO4 ubiquitination, we performed in silico prediction using UbiBrowser 3.0 (http://ubibrowser.bio-it.cn). Candidate ligases were prioritized by functional relevance, subcellular localization, and confidence score. The top five candidates, MDM2, BTRC, UBE4B, STUB1, and UBE4A, were subjected to shRNA-mediated KD in MLE-12 cells for initial functional screening. Of these, only MDM2-KD significantly reduced FOXO4 ubiquitination, prompting further mechanistic investigation (Fig. S2A–E). Consistently, MDM2-OE in MLE-12 cells decreased FOXO4 protein levels, an effect reversed by MG132, confirming proteasomal degradation (Fig. S2F). To validate direct physical interaction, endogenous and exogenous Co-IP assays in MLE-12 and HEK293T cells confirmed MDM2-FOXO4 binding (Fig. S2G–H). Moreover, endogenous Co-IP experiments revealed that MDM2-OE markedly enhanced FOXO4 ubiquitination (Fig. S2I), a finding further corroborated in an exogenous expression system (Fig. S2J). To determine whether FOXO4 succinylation influences MDM2-mediated ubiquitination, Co-IP assays demonstrated that the FOXO4-K139E mutant markedly enhanced MDM2-dependent FOXO4 ubiquitination, whereas the FOXO4-K139R mutant significantly suppressed this modification (Fig. S2K). Furthermore, Co-IP assays also indicated that the K48R mutation markedly attenuated MDM2-dependent ubiquitination of FOXO4, while the K63R mutation exerted no discernible effect (Fig. S2L). Notably, the FOXO4-K139E mutant enhanced the physical interaction between FOXO4 and MDM2 (Fig. S2M). Collectively, these findings indicate that FOXO4 succinylation at K139 enhances its binding affinity for MDM2, thereby facilitating MDM2-dependent K48-linked polyubiquitination and subsequent degradation via the ubiquitin-proteasome system.

Finally, we investigated the impact of SIRT7 on FOXO4 subcellular localization. Both subcellular fractionation followed by Western blotting and immunofluorescence microscopy confirmed that SIRT7-KO substantially reduced nuclear accumulation of FOXO4, whereas SIRT7-OE increased nuclear FOXO4 levels (Fig. S3A–B).

### 3.5 SIRT7 deficiency aggravates LPS-induced ferroptosis in vivo

**Fig. 5:**
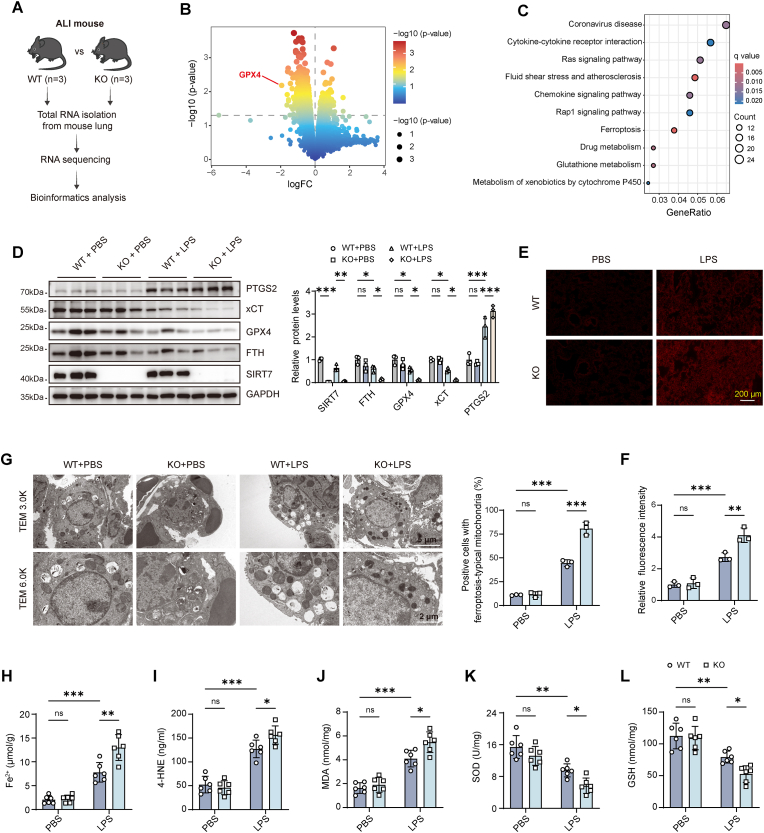
SIRT7 deficiency aggravates LPS-triggered ferroptosis **in vivo. A** Study designed RNA sequencing to understand lung tissues transcriptome alterations in SIRT7-KO ALI mice (n = 3). **B** Volcano plot depicting differentially expressed genes in lung tissue of SIRT7-KO mice with ALI (n = 3). Differential expression analysis was conducted using a two-sided statistical test, and *p*-values were adjusted for multiple testing via the Benjamini-Hochberg procedure to control the false discovery rate (FDR). **C** Bubble plot depicting the enrichment of KEGG pathways among differentially expressed genes. **D** Western blotting analysis of ferroptosis-associated protein markers (PTGS2, xCT, GPX4, and FTH1) in lung tissues from SIRT7-KO mice challenged with LPS (n = 3). One-way ANOVA followed by Tukey's post-hoc test for multiple comparisons. **E-F** DHE staining revealed the impact of SIRT7-KO on ROS levels in the lung tissue of LPS-induced ALI mice (n = 3, scale bar = 200 μm). Two-way ANOVA followed by Tukey's post-hoc test for multiple comparisons. **G** Blind quantification of positively stained cells with ferroptosis-typical mitochondrial morphology across designated experimental groups in mouse lung tissue. Representative TEM images are shown (n = 3, up: scale bar = 5 μm; down: scale bar = 2 μm). Two-way ANOVA followed by Tukey's post-hoc test for multiple comparisons. **H-L** Levels of Fe^2+^, 4-HNE, MDA, SOD, and GSH (n = 6). Two-way ANOVA followed by Tukey's post-hoc test for multiple comparisons. Data are presented as mean ± SD. Each experiment was conducted with three or six independent biological replicates. *p < 0.05, **p < 0.01, and ***p < 0.001; ns, not significant.

To elucidate the molecular regulatory mechanisms by which SIRT7 drives the pathogenesis and progression of ALI, we performed RNA sequencing (RNA-seq) on lung tissues from SIRT7-KO and WT mice subjected to LPS (Fig. 5A). Differential expression analysis unveiled 932 DEGs, with 122 up-regulated and 810 down-regulated. To validate RNA-seq data reliability, we performed qPCR on the six most DEGs ranked by absolute log_2_FC (|log_2_FC|): MYL12B (log_2_FC = −5.59), GPX4 (−1.94), SLC7A11/xCT (−1.62), ACSL6 (+1.62), SAT1 (+1.62), and CLDN1 (+1.13). qPCR results showed directional and quantitative concordance with RNA-seq measurements, confirming the robustness and reproducibility of our transcriptomic profiling (Fig. S4A–G). KEGG pathway enrichment analysis demonstrated that these DEGs were significantly enriched in the ferroptosis and glutathione metabolic pathways (Fig. 5B–C). Notably, expression of GPX4, a pivotal enzyme in glutathione metabolism and a central negative regulator of ferroptosis, was significantly down-regulated. Thus, these data indicate that SIRT7 likely serves as a negative regulator of LPS-induced ferroptosis. To corroborate the aforementioned findings, Western blotting was performed to quantify expression changes of key ferroptosis-related proteins in a murine ALI model. The results demonstrated that ALI induction significantly reduced protein levels of GPX4, ferritin heavy chain 1 (FTH1), and solute carrier family 7 member 11 (SLC7A11/xCT), while substantially increasing prostaglandin-endoperoxide synthase 2 (PTGS2) expression. SIRT7-KO markedly amplified these alterations, exhibiting more pronounced suppression of GPX4, FTH1, and SLC7A11/xCT along with enhanced PTGS2 upregulation (Fig. 5D). Ultrastructural analysis by TEM revealed characteristic ferroptotic mitochondrial pathology in LPS-challenged lungs, manifested by mitochondrial shrinkage, increased membrane density, and cristae degeneration (Fig. 5G–Table S10). These pathological changes were exacerbated in SIRT7-KO mice, consistent with the enhanced tissue damage observed in H&E-stained sections. Biochemical assessments showed significant elevations in pulmonary ROS, labile Fe^2+^, 4-HNE, and MDA following LPS exposure, with further augmentation upon SIRT7-KO (Fig. 5E–F, H-J). Conversely, LPS administration substantially decreased SOD activity and GSH levels, effects that were potentiated by SIRT7-KO (Fig. 5K–L). These collective findings establish that SIRT7 deficiency drives ferroptosis through coordinated dysregulation of ferroptosis-executing proteins, mitochondrial ultrastructural disintegration, and potentiation of oxidative stress signaling.

### 3.6 SIRT7 negatively regulates LPS-induced ferroptosis in vitro

**Fig. 6:**
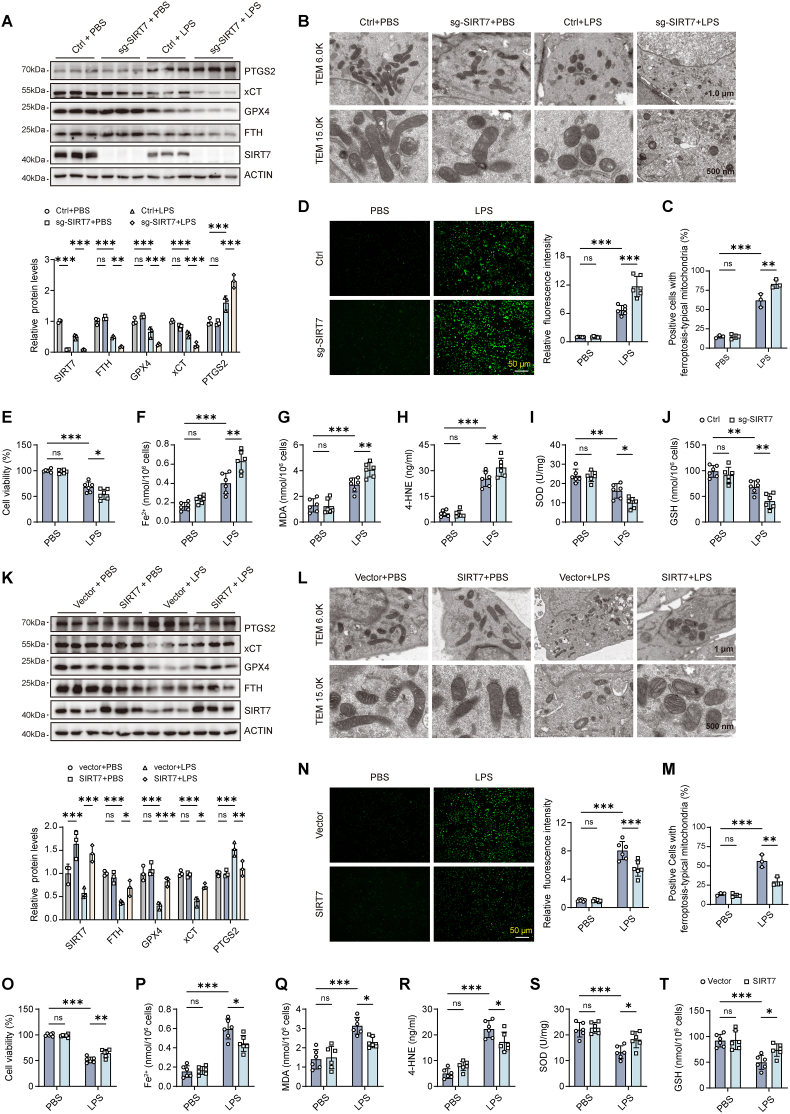
SIRT7 acts as a negative regulator of LPS-induced ferroptosis i**n vitro. A** Western blotting analysis of ferroptosis-related markers (PTGS2, xCT, GPX4, and FTH1) in SIRT7-KO MLE-12 cells (n = 3). One-way ANOVA followed by Tukey's post-hoc test for multiple comparisons. **B–C** Blind quantification of positively stained cells with ferroptosis-typical mitochondrial morphology across designated experimental groups in MLE-12 cells. Representative TEM images are shown (n = 3, up: scale bar = 1 μm; down: scale bar = 500 nm). Two-way ANOVA followed by Tukey's post-hoc test for multiple comparisons. **D** Assessment of cellular ROS levels in MLE-12 cells following SIRT7-KO using the DCFH-DA fluorescent probe (n = 6, scale bar = 50 μm). Representative images are shown. Two-way ANOVA followed by Tukey's post-hoc test for multiple comparisons. **E** CCK-8 assay to assess the impact of SIRT7-KO on MLE-12 cell viability (n = 6). Two-way ANOVA followed by Tukey's post-hoc test for multiple comparisons. **F-J** The effects of SIRT7-KO on intracellular Fe^2+^, MDA, 4-HNE, SOD, and GSH levels in MLE-12 cells (n = 6). Two-way ANOVA followed by Tukey's post-hoc test for multiple comparisons. **K** Western blotting analysis of ferroptosis-related markers (PTGS2, xCT, GPX4, and FTH1) in SIRT7-OE (SIRT7) MLE-12 cells (n = 3). One-way ANOVA followed by Tukey's post-hoc test for multiple comparisons. **L-M** Blind quantification of positively stained cells with ferroptosis-typical mitochondrial morphology across designated experimental groups in MLE-12 cells. Representative TEM images are shown (n = 3, up: scale bar = 1 μm; down: scale bar = 500 nm). Two-way ANOVA followed by Tukey's post-hoc test for multiple comparisons. **N** Assessment of cellular ROS levels in MLE-12 cells following SIRT7-OE using the DCFH-DA fluorescent probe (n = 6, scale bar = 50 μm). Representative images are shown. Two-way ANOVA followed by Tukey's post-hoc test for multiple comparisons. **O** CCK-8 assay to assess the impact of SIRT7-OE on MLE-12 cell viability (n = 6). Two-way ANOVA followed by Tukey's post-hoc test for multiple comparisons. **P-T** The effects of SIRT7-OE on intracellular Fe^2+^, MDA, 4-HNE, SOD, and GSH levels in MLE-12 cells (n = 6). Two-way ANOVA followed by Tukey's post-hoc test for multiple comparisons. LPS was applied at a concentration of 10 μg/mL. Data are presented as mean ± SD. Each experiment was conducted with three or six independent biological replicates. *p < 0.05, **p < 0.01, and ***p < 0.001; ns, not significant.

Subsequently, we systematically examined the role of SIRT7 in LPS-induced ferroptosis in vitro. Since multiple forms of regulated cell death, including apoptosis, necroptosis, and PANoptosis, are common in LPS-ALI models and may confound experimental interpretation, we used mechanistically specific inhibitors to dissect the contribution of each pathway. CCK-8 and LDH release assays demonstrated that only the ferroptosis specific inhibitor (Fer-1) significantly attenuated LPS-induced cytotoxicity in SIRT7-KO MLE-12 cells, whereas the pan-caspase inhibitor (Z-VAD-FMK) and the NLRP3 inflammasome inhibitor (MCC950) provided no measurable protection (Fig. S5A–B). These results demonstrate that ferroptosis is the predominant mode of LPS-induced cell death in SIRT7-KO MLE-12 cells. Consistent with the above-mentioned in vivo, LPS challenge triggered characteristic ferroptotic responses, evidenced by downregulation of GPX4, SLC7A11/xCT, and FTH1, alongside PTGS2 upregulation. Ultrastructural analysis revealed mitochondrial shrinkage, condensed double-membrane density, and cristae loss, consistent with the established morphological hallmarks of ferroptosis. Biochemical analyses demonstrated elevated total ROS, lipid ROS, Fe^2+^, 4-HNE, and MDA levels, coupled with reduced cell viability, SOD activity, and GSH content. SIRT7-KO exacerbated these alterations (Fig. 6A–J, Fig. S6A, Table S11), whereas SIRT7-OE attenuated LPS-induced ferroptotic phenotypes in MLE-12 cells (Fig. 6K–T, Fig. S6B Table S12). These results establish SIRT7 as a negative regulator of ferroptosis in vitro.

### 3.7 Ferroptosis inhibitors attenuate whereas inducers exacerbate LPS-induced ALI and inflammation in vivo and in vitro

**Fig. 7:**
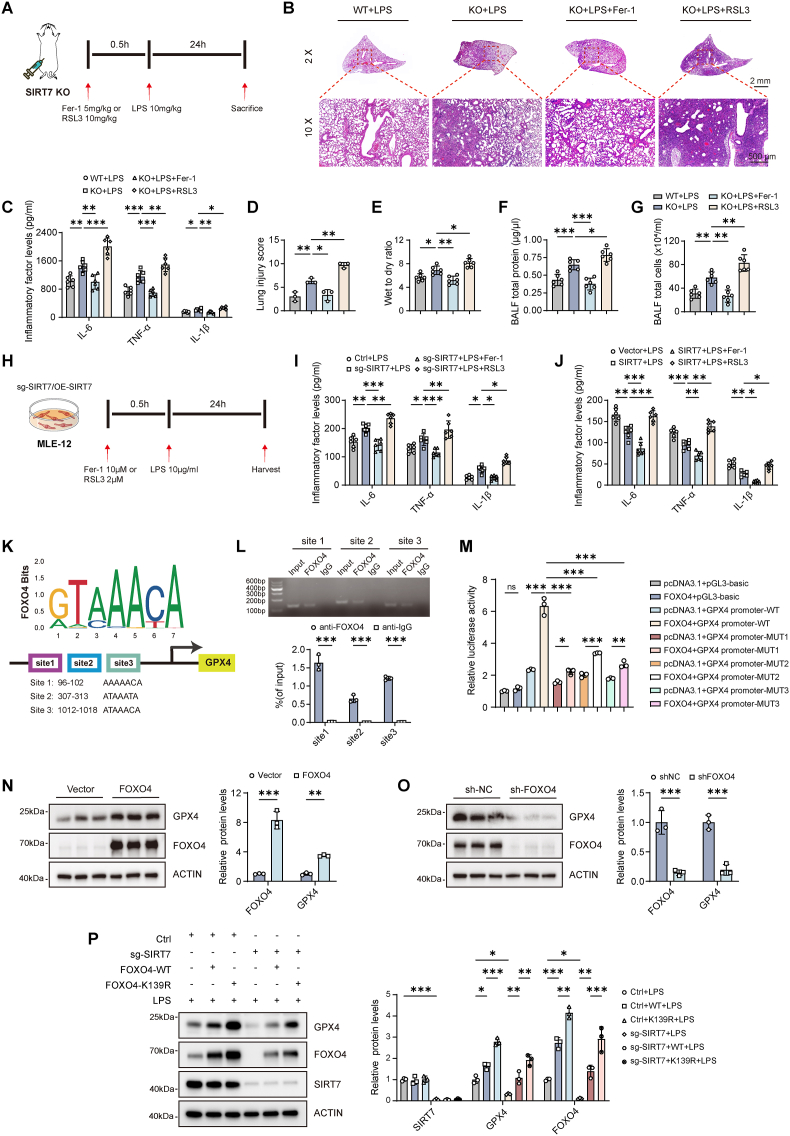
SIRT7 attenuates ferroptosis by regulating the FOXO4-GPX4 signaling **pathway. A** Schematic diagram of pharmacological inhibition or activation of ferroptosis intervention in SIRT7-KO mice. **B** Representative H&E-stained lung tissue sections in mice described in (A) (n = 3, up: scale bar = 2 mm; down: scale bar = 500 μm). **C** Levels of pro-inflammatory factors (IL-6, TNF-α and IL-1β) in BALF from mice across different groups as described in (A) (n = 6). One-way ANOVA followed by Tukey's post-hoc test for multiple comparisons. **D** Lung injury score across different groups as described in (A) (n = 3). One-way ANOVA followed by Tukey's post-hoc test for multiple comparisons. **E** W/D weight ratio across different groups as described in (A) (n = 6). One-way ANOVA followed by Tukey's post-hoc test for multiple comparisons. **F-G** Levels of total protein and total cells in BALF from mice across different groups as described in (A) (n = 6). One-way ANOVA followed by Tukey's post-hoc test for multiple comparisons. **H** Schematic diagram of pharmacological inhibition or activation of ferroptosis in SIRT7-KO or SIRT7-OE MLE-12 cells. **I-J** Levels of pro-inflammatory factors (IL-6, TNF-α and IL-1β) in MLE-12 cells across different groups as described in (H) (n = 6). One-way ANOVA followed by Tukey's post-hoc test for multiple comparisons. **K** JASPAR database prediction of three putative FOXO4 binding sites in the GPX4 promoter. **L** ChIP-qPCR showed FOXO4 binding to three putative sites in the GPX4 promoter (n = 3). Student's t-test. **M** Dual-luciferase reporter analysis of FOXO4-mediated transcriptional activation of GPX4 in MLE-12 cells (n = 3). One-way ANOVA followed by Tukey's post-hoc test for multiple comparisons. **N–O** Western blotting analysis of GPX4 protein expression in MLE-12 cells after FOXO4-OE or KD (n = 3). Student's t-test. **P** FOXO4-OE rescues GPX4 expression in SIRT7-KO MLE12 cells, with the desuccinylation-mimicking mutant K139R exerting a stronger effect than WT (n = 3). One-way ANOVA followed by Tukey's post-hoc test for multiple comparisons. LPS was applied at a concentration of 10 μg/mL. Data are presented as mean ± SD. Each experiment was conducted with three or six independent biological replicates. *p < 0.05, **p < 0.01, and ***p < 0.001; ns, not significant.

Based on prior evidence that SIRT7 regulates LPS-induced ferroptosis in vivo and in vitro, we investigated the effects of pharmacological ferroptosis modulation with the inhibitor (Fer-1) or the inducer RSL3 on LPS-induced ALI. ALI was induced in SIRT7-KO and WT mice by intratracheal LPS instillation. Thirty minutes before LPS administration, KO mice were intraperitoneally injected with Fer-1 (5 mg/kg) or RSL3 (10 mg/kg) (Fig. 7A) [7,38,39]. Histopathological assessment via H&E staining showed that LPS exposure induced characteristic acute pulmonary inflammation in WT mice, such as neutrophilic infiltration, alveolar wall thickening and fusion, focal hemorrhage, alveolar edema, and structural disruption. These pathological changes were significantly more severe in SIRT7-KO mice than in WT mice. Fer-1 pretreatment (KO + LPS + Fer-1 group) markedly attenuated all histopathological lesions in SIRT7-KO mice, along with significant decreases in lung injury scores and W/D weight ratios (Fig. 7B and 7D-E). In contrast, RSL3 pretreatment (KO + LPS + RSL3 group) exacerbated histopathological damage and increased injury scores and W/D weight ratios relative to the KO + LPS group (Fig. 7B and 7D-E). Correspondingly, analysis of BALF indicated that Fer-1 pretreatment significantly reduced total cell counts, total protein concentrations, and levels of pro-inflammatory cytokines, including IL-6, TNF-α, and IL-1β, in SIRT7-KO mice, whereas RSL3 pretreatment significantly elevated these markers compared to the KO + LPS group (Fig. 7C and 7F-G).

Then, we investigated the effects of Fer-1 and RSL3 using an established in vitro model of LPS-stimulated MLE-12 cells. Cells were pretreated with Fer-1 (10 μM) or RSL3 (2 μM) for 30 min prior to a 24-h challenge with LPS (Fig. 7H) [7,40]. Compared to control cells (Ctrl + LPS), SIRT7 deficiency markedly potentiated LPS-induced secretion of the pro-inflammatory cytokines IL-6, TNF-α, and IL-1β. This exacerbated inflammatory response in SIRT7-KO cells was significantly attenuated by Fer-1 but further amplified by RSL3 (Fig. 7I). Conversely, SIRT7-OE itself conferred a robust anti-inflammatory phenotype, substantially suppressing cytokine release upon LPS stimulation. In these SIRT7-OE cells, Fer-1 pretreatment synergistically enhanced this suppression, whereas RSL3 completely abrogated the protective effect, leading to a substantial increase in the concentrations of pro-inflammatory mediators (Fig. 7J). Together, these data mechanistically corroborate that ferroptosis inhibition attenuates, while its induction aggravates, LPS-driven inflammatory pathology, aligning with our in vivo observations.

### 3.8 SIRT7 suppresses ferroptosis by modulating the FOXO4-GPX4 signaling axis

Previous studies have established GPX4 as a critical negative regulator of ferroptosis [41,42]. Our prior work demonstrated that SIRT7 positively modulates GPX4 expression and thereby attenuates LPS-induced ferroptosis in both in vitro and in vivo models (Figs. 5C–6A and 6J). To determine whether SIRT7 mediates this protective effect through the FOXO4-GPX4 signaling axis, we performed a series of functional and mechanistic investigations. Bioinformatics analysis of the GPX4 promoter region via the JASPAR database (https://jaspar.genereg.net) [43] identified three putative FOXO4 binding motifs: site 1 (AAAAACA), site 2 (ATAAATA), and site 3 (ATAAACA) (Fig. 7K). ChIP-qPCR confirmed FOXO4 binding to all three sites, with the highest enrichment observed at site 1 (Fig. 7L). We therefore generated three site-specific mutant promoters (MUT1, MUT2, and MUT3) and evaluated FOXO4-dependent transcription using dual-luciferase reporter assays in MLE-12 cells. FOXO4-OE (FOXO4 + GPX4 promoter WT) significantly enhanced wild-type GPX4 promoter activity compared with the pcDNA3.1 + GPX4 promoter-WT group, whereas all three mutants markedly attenuated this activation, with the most pronounced effect observed at MUT1 (Fig. 7M). Correspondingly, Western blotting showed that FOXO4-OE increased GPX4 protein levels, whereas FOXO4-KD decreased them (Fig. 7N–O). To determine whether SIRT7-mediated GPX4 regulation depends on FOXO4, we performed a rescue experiment in SIRT7-KO MLE-12 cells. As hypothesized, SIRT7-KO markedly reduced GPX4 protein levels, an effect that was rescued by FOXO4-OE. More importantly, in direct comparison, the desuccinylation mimic FOXO4-K139R restored GPX4 expression significantly more efficiently than FOXO4-WT (Fig. 7P). Take together, our data establish that SIRT7 mitigates ferroptosis in LPS-induced ALI by upregulating the FOXO4-GPX4 axis.

### 3.9 Desuccinylation of FOXO4 at K139 protects against LPS-induced ALI in SIRT7-KO mice

**Fig. 8:**
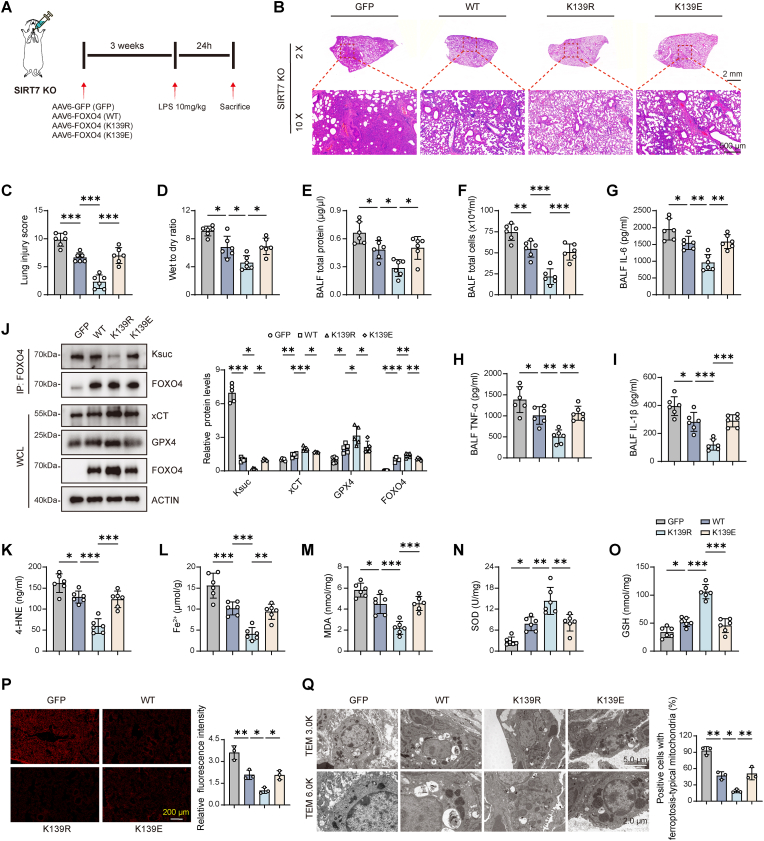
FOXO4 desuccinylation at K139 protects against LPS-challenged ALI in SIRT7-**KO mice. A** Schematic illustration of AAV6-mediated intervention in SIRT7-KO mice. Intratracheal LPS instillation to induce ALI following a 3-week indicated AAV6 vector delivery period. **B–C** Representative H&E-stained lung tissue sections and quantitative lung injury scores in SIRT7-KO mice across different groups described in (A) (n = 6, up: scale bar = 2 mm; down: scale bar = 500 μm). One-way ANOVA followed by Tukey's post-hoc test for multiple comparisons. **D-I** Lung W/D weight ratio and BALF parameters, including total cells, total protein, and pro-inflammatory cytokines (IL-6, TNF-α and IL-1β) levels in SIRT7-KO mice across different groups described in (A) (n = 6). One-way ANOVA followed by Tukey's post-hoc test for multiple comparisons. **J** Succinylated FOXO4 protein levels across different groups described in (A) (n = 5). One-way ANOVA followed by Tukey's post-hoc test for multiple comparisons. **K–O** Levels of 4-HNE Fe^2+^, MDA, SOD, and GSH across different groups described in (A) (n = 6). One-way ANOVA followed by Tukey's post-hoc test for multiple comparisons. **P** Representative DHE staining of lung tissue sections showing ROS levels across different groups described in (A) (n = 3). One-way ANOVA followed by Tukey's post-hoc test for multiple comparisons. **Q** Blind quantification of positively stained cells with ferroptosis-typical mitochondrial morphology across different groups described in (A) in mouse lung tissue. Representative TEM images are shown (n = 3, up: scale bar = 5 μm; down: scale bar = 2 μm). One-way ANOVA followed by Tukey's post-hoc test for multiple comparisons. Data are presented as mean ± SD. Each experiment was conducted with three, five, or six independent biological replicates. *p < 0.05, **p < 0.01, and ***p < 0.001.

To further elucidate the in vivo function of SIRT7-mediated FOXO4 desuccinylation in LPS-induced ALI, systemic SIRT7-KO mice were intratracheally administered AAV6 vectors encoding GFP (control), FOXO4-WT, FOXO4-K139R, or FOXO4-K139E. Following this intervention, ALI was induced via intratracheal LPS instillation to evaluate the effects of each treatment on ALI parameters (Fig. 8A). Histological assessment by H&E staining revealed that lung tissues from control mice displayed typical features of severe inflammatory injury, characterized by extensive neutrophil infiltration, diffuse thickening and fusion of alveolar walls, focal hemorrhage, alveolar edema, and disruption of tissue architecture. In comparison, mice expressing FOXO4-WT, FOXO4-K139R, or FOXO4-K139E exhibited significantly attenuated lung injury, as reflected by markedly lower histopathological scores and reduced lung W/D weight ratios (Fig. 8B–D). Notably, the most substantial histological improvement was observed in the FOXO4-K139R group (Fig. 8B–D). Correspondingly, analysis of BALF indicated that all FOXO4-expressing groups had significantly decreased total cell counts, total protein concentrations, and levels of the pro-inflammatory cytokines IL-6, TNF-α, and IL-1β compared with the control group, with the most pronounced reductions occurring in the FOXO4-K139R group (Fig. 8E–I).

Subsequent molecular analysis of mouse lung tissue samples from the four cohorts focused on ferroptosis pathways, yielding results congruent with the graded histopathological lung injury. Immunoblotting indicated that expression of the key ferroptosis-inhibitory proteins GPX4 and SLC7A11/xCT was significantly upregulated in the three treatment groups compared to the control, with the most substantial increase observed in the FOXO4-K139R group (Fig. 8J). Correspondingly, succinylation at the K139 site of FOXO4 was markedly decreased across treatment groups, reaching its lowest level in the K139R mutant group (Fig. 8J). Assessment of biochemical markers revealed significantly lower pulmonary concentrations of ROS, labile Fe^2+^, 4-HNE, and MDA in the treatment groups relative to the control, with the FOXO4-K139R group exhibiting the greatest reduction (Fig. 8K–M, 8P). In contrast, antioxidant defenses, as measured by SOD activity and GSH levels, were significantly potentiated, most notably in the FOXO4-K139R group (Fig. 8N–O). Ultrastructural examination via TEM confirmed that mitochondria from control mice exhibited characteristic ferroptotic morphology, including contraction, heightened membrane density, and cristae loss. These pathological mitochondrial changes were markedly attenuated in the treatment groups, with the FOXO4-K139R group demonstrating the most preserved mitochondrial architecture (Fig. 8Q–Table S13). Collectively, these in vivo data establish that SIRT7 mitigates LPS-induced ALI by desuccinylating FOXO4 at K139.

### 3.10 SIRT7 agonists mitigate ALI in LPS-challenged mice

**Fig. 9:**
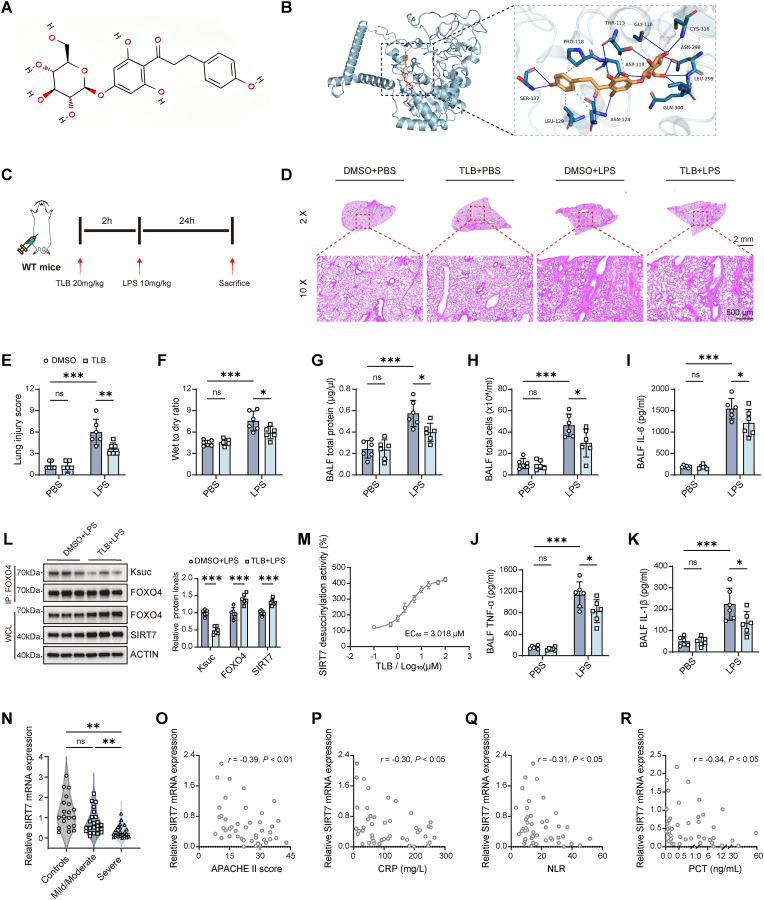
SIRT7 agonists mitigate LPS-induced ALI **in vivo. A** The molecular architecture of SIRT7**. B** Molecular docking revealed a putative binding mode and favorable affinity between TLB and SIRT7. **C** Schematic representation of TLB administration in C57BL/6J mice. **D-E** Representative H&E-stained lung tissue sections and quantitative lung injury scores in C57BL/6J mice pretreated with DMSO or TLB prior to LPS challenge (n = 6, up: scale bar = 2 mm; down: scale bar = 500 μm). Two-way ANOVA followed by Tukey's post-hoc test for multiple comparisons. **F–K** Lung W/D weight ratio and BALF parameters, including total cells, total protein, and pro-inflammatory cytokines (IL-6, TNF-α and IL-1β) levels in C57BL/6J mice pretreated with DMSO or TLB prior to LPS challenge (n = 6). Two-way ANOVA followed by Tukey's post-hoc test for multiple comparisons. **L** Protein expression levels of SIRT7 and FOXO4, as well as succinylation status of FOXO4, in lung tissue from C57BL/6J mice pretreated with DMSO or TLB prior to LPS challenge (n = 6). Student's t-test. **M** TLB directly activates SIRT7 desuccinylase activity in vitro (n = 3). Recombinant human SIRT7 was incubated with the H3K122suc-AMC fluorescent peptide substrate in the presence of increasing concentrations of TLB (0.1-100 μM). Relative enzymatic activity was normalized to the vehicle control (0 μM TLB, set as 100%). The dose-response curve was fitted using a four-parameter logistic (4 PL) model, yielding an EC_50_ of 3.018 μM (95% CI: 2.304-3.968 μM), a Hill slope of 1.103 (95% CI: 0.826-1.431), and R^2^ = 0.977. **N** SIRT7 mRNA levels quantified by qPCR in BALF from controls and patients with ARDS across severity grades (mild/moderate and severe). Kruskal-Wallis followed by Dunn's post-hoc test for multiple comparisons. **O–R** Correlation analysis of SIRT7 mRNA levels in BALF with ARDS severity and systemic inflammatory biomarkers, including the APACHE II score, serum C-reactive protein (CRP), neutrophil-to-lymphocyte ratio (NLR), and procalcitonin (PCT). Correlations were assessed using Spearman's rank-order correlation coefficient. All data from animal and in vitro experiments are expressed as mean ± SD, with each experiment performed using three or six independent biological replicates. *p < 0.05, **p < 0.01, and ***p < 0.001; ns, not significant.

TLB has been shown to selectively upregulate SIRT7 expression, thereby attenuating cerebral I/R injury [44]. Separately, the aqueous extract of sweet tea (WEL), in which phlorizin and TLB are putative bioactive constituents, ameliorates LPS-induced ALI [45]. However, the potential involvement of TLB in mediating SIRT7-dependent protection against LPS-induced ALI remains unclear. Molecular docking revealed a strong direct interaction between TLB and SIRT7 (binding energy: - 7.8 kcal/mol) (Fig. 9A–B). To evaluate therapeutic efficacy, we pre-treated mice with TLB (20 mg/kg, i.p.) 2 h before LPS challenge (Fig. 9C). Compared to the DMSO + LPS group, TLB markedly attenuated pathological lung injury, as demonstrated by reduced neutrophil infiltration, preserved alveolar architecture, diminished hemorrhage and edema, and significantly lower lung injury scores and W/D weight ratios (Fig. 9D–F). Consistent with this, TLB reduced total cell count, protein concentration, and levels of key pro-inflammatory mediators (IL-6, TNF-α, and IL-1β) in BALF (Fig. 9G–K). Immunoblotting demonstrated that TLB not only upregulated SIRT7 protein expression in lung tissues but also specifically reduced the succinylation level of FOXO4 (Fig. 9L). To evaluate whether TLB directly modulates SIRT7 enzymatic activity, we performed an in vitro desuccinylase assay using recombinant SIRT7 protein and a fluorescent H3K122suc-AMC peptide. As shown in Fig. 9M, TLB stimulated SIRT7 mediated desuccinylation in a concentration dependent manner, with an EC_50_ of 3.018 μM (95% CI: 2.304-3.968 μM) and a Hill slope of 1.103 (95% CI: 0.826-1.431). Maximum activation reached 4.242-fold relative to the vehicle control, with excellent fit (R^2^ = 0.977). Collectively, our study demonstrates that TLB upregulates SIRT7 expression in vivo and potentiates its desuccinylase activity in vitro, establishing a dual mode of action involving both transcriptional upregulation and direct enzymatic activation. Through this combined mechanism, TLB effectively attenuates LPS-induced ALI, underscoring its potential as a therapeutic candidate.

Finally, to elucidate the pathophysiological role of SIRT7 in ARDS, BALF was obtained from 45 ARDS patients and 20 healthy controls for qPCR analysis (Table S1). SIRT7 mRNA expression in BALF was significantly downregulated in ARDS patients compared with controls, with the most pronounced reduction observed in severe cases (Fig. 9N). Furthermore, SIRT7 mRNA levels demonstrated a strong inverse correlation with established markers of disease severity and systemic inflammation, including the APACHE II score, serum C-reactive protein (CRP), neutrophil-to-lymphocyte ratio (NLR), and procalcitonin (PCT) (Fig. 9O–R). These data suggest that SIRT7 likely exerts a protective, anti-inflammatory function in ARDS, and that its downregulation is linked to both aggravated clinical pathology and an enhanced inflammatory state.

## 4 Discussions

**Fig. 10:**
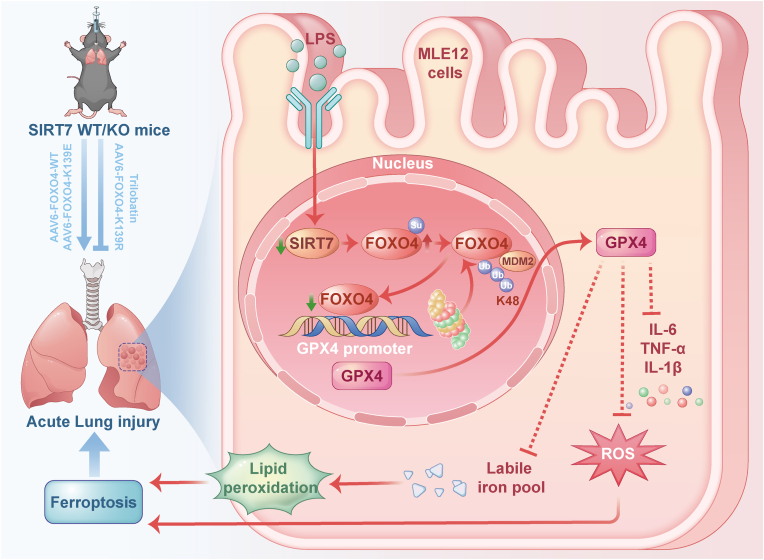
Graphic overview. Schematic illustration of a mechanistic pathway wherein SIRT7 desuccinylated FOXO4 to suppress ferroptosis and mitigate LPS-induced ALI.

Although prior studies have implicated SIRT7 in regulating metabolism, aging, and inflammation [[17], [18], [19], [20]], its exact role and mechanism in ALI pathogenesis remain poorly defined. Here, we identify that SIRT7 protects against ALI by inhibiting AECs ferroptosis via the FOXO4-GPX4 axis. SIRT7 protein was significantly downregulated in LPS-induced ALI models and in LPS-stimulated MLE-12 cells. Genetic gain- and loss-of-function experiments confirmed that SIRT7 attenuates ALI by suppressing ferroptosis. Mechanistically, SIRT7 desuccinylates FOXO4 at K139, inhibiting MDM2-mediated K48-linked polyubiquitination, stabilizing FOXO4, maintaining nuclear retention, and subsequently upregulating GPX4 expression to limit ferroptosis. Furthermore, AAV6-mediated delivery of the desuccinylated FOXO4-K139R mutant significantly reduces lung pathological injury in SIRT7-KO mice. Notably, pharmacological activation of SIRT7 by TLB significantly attenuates LPS-induced ALI in mice (Fig. 10). Clinically, SIRT7 mRNA levels in BALF from patients with ARDS were markedly decreased and inversely correlated with disease severity. Taken together, our findings demonstrate for the first that the SIRT7-FOXO4 axis represents a critical pathway in ALI and a promising therapeutic target.

Protein lysine succinylation has emerged as a critical PTM with profound regulatory roles in protein localization, activity, and structural integrity [12,15,16]. Dysregulation of this modification is increasingly implicated in the pathogenesis of diverse diseases, such as cancer, I/R injury, chronic inflammatory disorders, and metabolic syndromes. For instance, Yu et al. demonstrated that acetaminophen-induced liver injury elevates pan-succinylation, and that SIRT5 attenuates this injury by specifically desuccinylating ALDH2 at K385 [46]. Separately, Wang et al. linked oxidative stress-induced hyper-succinylation of UCP1 at K56 and K151 in adipose tissue to protein destabilization and disrupted fatty acid oxidation homeostasis [47]. Through integrated succinylome and proteome analysis, Zhou et al. further identified 384 differentially expressed proteins and 749 altered succinylation sites in smoke-induced lung injury, highlighting the broad involvement of succinylated proteins in lung injury-related pathways [48]. In this study, we report a pronounced increase in pan-succinylation in LPS-challenged murine lung tissues and MLE-12 cells, which coincided with downregulation of the nuclear desuccinylase SIRT7. Collectively, our data posit SIRT7 as a pivotal regulator of ALI pathogenesis, exerting its function through the precise control of protein succinylation dynamics.

SIRT7 is a nucleolar-enriched, NAD^+^-dependent deacylase within the sirtuin family, possessing selective long-chain deacylase activity. In addition to deacetylation, it catalyzes several lysine acylations, such as succinylation and glutarylation, thereby regulating diverse cellular pathways [[17], [18], [19], [20]]. A growing body of evidence reveals a context-dependent, dual role for SIRT7 in inflammatory diseases, impacting pathogenesis, progression, and outcomes. For instance, SIRT7 deficiency in mice promotes progressive myocardial hypertrophy and dilated cardiomyopathy, accompanied by inflammatory infiltration and fibrosis (Vakhrusheva et al.) [49]. Sun et al. reported that SIRT7 inhibits Pg-LPS-driven proinflammatory cytokine release via the AKT/mTOR axis [50]. Another study identified SIRT7 as a key effector mediating the protective action of miR-762 in experimental ALI (Wang et al.) [51]. Conversely, SIRT7 can exert pro-inflammatory effects in certain settings. Its absence attenuates cisplatin-induced acute kidney injury (Miyasato et al.) [52], while its pharmacological inhibition reduces NF-κB-dependent inflammatory mediator secretion in pulmonary endothelial cells (Wyman et al.) [53]. In murine colitis, SIRT7 expression is upregulated, and its inhibition alleviates inflammation (Kim et al.) [54]. Myeloid-specific SIRT7-KO also ameliorates alcohol-induced liver injury and inflammation (Wang et al.) [55]. This functional duality suggests that SIRT7's role is governed by tissue context, disease model, pathological stage, and specific substrates. Our study demonstrates that SIRT7 deletion exacerbates LPS-induced lung inflammation and histopathological damage. Notably, in BALF from patients with ARDS, SIRT7 mRNA levels inversely correlate with disease severity. These data imply a protective role for SIRT7 in ALI. Future work should elucidate the context dependent regulatory mechanisms governing the dual functions of SIRT7.

FOXO4 is a pivotal transcription factor that orchestrates diverse biological processes, including inflammatory responses, cellular metabolism, oxidative stress adaptation, and programmed cell death [[21], [22], [23], [24], [25], [26], [27], [28]]. Its activity is precisely regulated by an array of PTMs. For instance, under oxidative stress, USP7 deubiquitinates FOXO4 to facilitate its nuclear translocation and enhance target gene transactivation (van der Horst et al.) [25]. In cortical neurons subjected to oxygen-glucose deprivation/reperfusion, Smurf2-mediated ubiquitination of FOXO4 modulates pyroptosis (Yan et al.) [24]. Oxidative stress also induces O-GlcNAcylation of FOXO4, augmenting its transcriptional output (Ho et al.) [26]. Furthermore, CBP-catalyzed acetylation of FOXO4 suppresses the Wnt/β-catenin pathway, promoting postmenopausal osteoporosis (Huang et al.) [27], whereas SIRT1-mediated deacetylation maintains extracellular matrix homeostasis, protecting against osteochondritis dissecans (Ma et al.) [28]. Additionally, SIRT7 regulates FOXO4 acetylation in idiopathic pulmonary fibrosis (Choudhury et al.) [22]. Cumulatively, these studies delineate a multi-layered PTM network that finetunes FOXO4 function in a context-dependent manner.

Here, by combining IP-MS, molecular docking, and cell-based functional assays, we identified a direct interaction between SIRT7 and FOXO4 in ALI. Functionally, this interaction promoted FOXO4 nuclear accumulation and transcriptional activation of the antioxidant gene GPX4. Of note, the AlphaFold-predicted structures used for docking represented monomeric relaxed conformations. Given that FOXO4 is highly dynamic, particularly during interactions with PTM effectors, the predicted binding interface may not fully recapitulate the native complex. Thus, future validation via alanine scanning mutagenesis of the predicted interface residues combined with functional assays is required. Gain- and loss-of-function studies revealed that SIRT7 specifically catalyzed FOXO4 desuccinylation at K139, without affecting its acetylation, lactoylation, or crotonylation. Mechanistically, desuccinylation at K139 attenuated MDM2-mediated K48-linked polyubiquitination, thereby stabilizing the protein. Accordingly, in vivo, a succinylation-mimetic FOXO4 mutant (K139E) blunted the protective effect of SIRT7 against ALI, whereas a desuccinylation-mimetic mutant (K139R) potentiated it. MDM2 has been established as an E3 ubiquitin ligase that regulates both mono- and polyubiquitination of FOXO4 [56,57]. However, whether this regulatory mechanism is operative in the pathological setting of ALI remains unclear. In this study, we demonstrate that MDM2 specifically catalyzes K48-linked polyubiquitination of FOXO4, thereby targeting it for proteasomal degradation. Furthermore, succinylation of FOXO4 at K139 does not antagonize ubiquitination at the same residue; rather, it enhances MDM2-FOXO4 interaction and potentiates K48-linked ubiquitination, representing a functionally cooperative (e.g., “activation-type”) crosstalk among PTMs. Nevertheless, high-resolution structural insights, for example, from cross-linking mass spectrometry or cryo-electron microscopy, are still necessary to fully delineate the conformational dynamics governing this regulatory interface. To our knowledge, this work provides the first mechanistic evidence that SIRT7-mediated desuccinylation of FOXO4 at K139 controls its stability, nuclear localization, and transcriptional activity, defining a novel SIRT7-FOXO4 axis that protects against ALI. Our findings extend the known PTM landscape of FOXO4 and offer fresh insight into ALI pathogenesis. Clinically, the modification status or expression of SIRT7 and FOXO4 may serve as potential biomarkers for early diagnosis or prognosis of ARDS. However, future studies should evaluate the translational relevance of this axis across diverse lung injury models and patient cohorts to assess its therapeutic potential.

Ferroptosis is a regulated, iron-dependent cell death process driven by lethal lipid peroxidation and consequent oxidative damage. Substantial evidence has established its critical involvement in the pathogenesis and progression of ALI [6,7,9]. Recent studies implicate protein succinylation as a key PTM that mechanistically regulates ferroptotic signaling [[58], [59], [60], [61]]. Exemplifying this, SUCLA2 was shown to suppress ferroptosis in renal tubular epithelial cells by inhibiting SHMT2 succinylation (Lyu et al.) [58], while KAT2A-mediated H3K79 succinylation promotes ferroptosis in podocytes via SAT2 upregulation (Peng et al.) [59]. Furthermore, SIRT5-mediated desuccinylation of PRDX6 at K209 mitigates ferroptosis and sepsis-induced kidney injury (Lin et al.) [60], and CPT1A exerts a protective role in sepsis-associated ALI by enhancing ACSL4 succinylation to inhibit ferroptosis (Wang et al.) [61]. These studies collectively establish a pivotal crosstalk between succinylation modifications and ferroptosis in inflammatory pathologies. Notably, the sirtuin family SIRT7 has recently been implicated in ferroptotic regulation across disease contexts. For instance, SIRT7 activation was found to alleviate renal ferroptosis in hypertensive mice via the KLF15/Nrf2 axis (Li et al.) [62], whereas its deficiency exacerbates oxidative stress-induced ferroptosis in melanocytes, accelerating vitiligo progression through the SMAD3-ATF3-GPX4 pathway (Wu et al.) [41]. Aligning with this, our transcriptomic profiling revealed that SIRT7-KO exacerbates ferroptosis in ALI models. Pharmacological inhibition of ferroptosis markedly attenuated pulmonary histopathological damage and inflammatory responses in SIRT7-KO mice; conversely, administration of a ferroptosis agonist exacerbated these phenotypes. Further in vivo investigation demonstrated that K139E potentiated ferroptosis in lung tissue, whereas K139R exerted a protective effect. To our knowledge, this study is the first to establish that SIRT7 suppresses LPS-induced ferroptosis and concomitant lung injury through site-specific desuccinylation of FOXO4 at K139. Collectively, these findings define a novel SIRT7-FOXO4 axis and provide a mechanistically grounded foundation for ferroptosis-targeted therapeutic strategies in ALI. GPX4 is a central regulator of ferroptosis, and its expression is tightly controlled by multiple transcription factors [41,42]. As a member of the FOXO transcription factor family, FOXO4 is well known to mediate cellular responses to oxidative stress. Prior work has established that the related family member FOXO3A directly binds to the GPX4 promoter and enhances its transcription, thereby inhibiting ferroptosis [63]. Although FOXO4 and FOXO3A share substantial functional overlap in the regulation of cell death, redox homeostasis, and metabolism [64], whether FOXO4 similarly contributes to the transcriptional control of GPX4 remains unknown. Using ChIP-qPCR and luciferase reporter assays, we demonstrate that FOXO4 also directly binds to the GPX4 promoter and transactivates its expression under ALI conditions. Moreover, rescue experiments confirmed that FOXO4-K139R restored GPX4 expression significantly more efficiently than FOXO4-WT in SIRT7-KO MLE-12 cells. This work not only expands the regulatory spectrum of the FOXO family in ferroptosis but also identifies FOXO4 as a crucial transcription factor linking oxidative stress responses to GPX4 expression in lung pathology. Future studies are warranted to elucidate the detailed molecular interplay governing the FOXO4-GPX4 axis, to validate its relevance across broader pulmonary disease models, and to explore its therapeutic potential.

TLB, a naturally occurring flavonoid, has demonstrated consistent anti-inflammatory and antioxidant efficacy across diverse experimental paradigms of inflammation-associated diseases. Specifically, Gao et al. elucidated that TLB significantly attenuated neuroinflammation and oxidative stress subsequent to cerebral I/R injury by modulating the TLR4/NF-κB and Nrf2/Keap1 signaling axes [65]. In a model of fulminant hepatic failure, Hou et al. established the ROS/TLR4/NLRP3 inflammasome axis as a critical target of TLB, wherein it effectively suppressed inflammatory activation, oxidative stress, pyroptosis, and apoptotic cell death [66]. Chen et al. further demonstrated that in diabetic nephropathy, TLB ameliorated renal inflammation and pyroptosis via inhibition of the AGEs/RAGE/NF-κB pathway [67]. Notably, Zhong et al. delineated that TLB mitigated LPS-induced ALI through activation of the AMPK/GSK3β-Nrf2 signaling cascade [30]. This study uncovered a novel mechanistic insight that TLB upregulates SIRT7 expression in vivo and potentiates its desuccinylase activity in vitro, thereby conferring protection against LPS-induced ALI. While these findings collectively underscore TLB's multi-faceted therapeutic potential in ALI, critical knowledge gaps persist regarding its direct molecular targets, in vivo pharmacokinetic and pharmacodynamic properties, long-term toxicological safety, and clinical translatability for ALI management. Rigorous mechanistic studies and systematic translational research are therefore required to bridge these gaps and advance TLB toward clinical development.

Although our study establishes a critical role for the SIRT7-FOXO4 axis in AECs, ALI pathogenesis involves a complex interplay among multiple cell types, including resident macrophages and pulmonary microvascular endothelial cells. Our work, primarily based on the MLE-12 cell line, does not directly assess whether this regulatory axis functions similarly in these other populations. Nevertheless, accumulating evidence suggests cell-type-specific effects of this pathway. In macrophages, SIRT7 has been reported to regulate the inflammatory response via the NF-κB/CCL2 axis [55]. Besides, Zhu et al. demonstrated that FOXO4-KO macrophages, upon LPS stimulation in vitro, exhibited significantly elevated IL-6 secretion and ROS production [68]. Therefore, the SIRT7-FOXO4 axis may regulate inflammatory responses in alveolar macrophages. In endothelial cells, FOXO4 has been reported to specifically activate arginase 1 (Arg1) transcription during the post-myocardial infarction inflammatory response, thereby exacerbating neutrophil infiltration and upregulating pro-inflammatory factor expression [69]. Given that SIRT7-dependent desuccinylation can alter FOXO4 transcriptional activity, this axis may similarly influence inflammatory responses in endothelial cells during ALI. We fully acknowledge that the current data are limited to the epithelial compartment, and the in vivo role of the SIRT7-FOXO4 axis in macrophages and endothelial cells remains speculative. Future studies employing cell-specific SIRT7 or FOXO4 conditional KO mice, along with co-culture systems and single-cell analyses, will be essential to dissect the relative contributions of each cell type and to determine whether therapeutic targeting of this axis exerts beneficial or detrimental effects across different lung cell populations.

Our study has several limitations. First, the reliance on a systemic SIRT7-KO model precludes the precise attribution of observed phenotypes exclusively to AECs. Future investigations employing conditional SIRT7-KO models are warranted to definitively establish cell-autonomous mechanisms. Second, owing to ethical constraints in clinical research and limited availability of human lung tissue specimens, this study initially utilized BALF from ARDS patients for molecular analysis. Future studies should prioritize the acquisition of matched clinical lung tissue specimens to directly validate SIRT7 expression and functional activity at the histopathological level. Finally, direct evidence of FOXO4 succinylation at K139 in clinical specimens from patients with ARDS is currently absent, owing primarily to the unavailability of a site-specific antibody. Future studies aim to develop a site-specific antibody and apply IP-MS to prospectively collected BALF or lung tissue samples from patients with ARDS and control subjects to validate the correlation between this modification and clinical outcomes in larger cohorts.

In summary, our study provides novel evidence that SIRT7-mediated desuccinylation of FOXO4 attenuates LPS-induced ALI. These findings uncover a previously unrecognized SIRT7-FOXO4 axis in ALI pathogenesis and establish a mechanistic foundation for the development of targeted therapeutic strategies against ALI.

## Data availability

The datasets generated and analyzed during the current study are available from the corresponding author upon reasonable request.

## Ethics statement

This study was conducted in compliance with all relevant ethical guidelines. The protocols involving human participants were reviewed and approved by the Ethics Review Committee of Jinling Hospital, Medical School of Nanjing University (Approval No.: 2025DZGJJ-052) and adhered to the principles of the Declaration of Helsinki. Prior to biological sample or clinical data collection, each participant received detailed information regarding the study objectives, procedures, potential risks, and protective measures for their rights and welfare; written informed consent was obtained from all individuals before enrollment. All animal experiments were approved by the Experimental Animal Ethics Committee of Jinling Hospital, Medical School of Nanjing University (Approval No.: DZGDWLS202503000244) and performed in accordance with the Guide for the Care and Use of Laboratory Animals (NIH, 8th edition, USA).

## CRediT authorship contribution statement

**Kaikai Shen:** Conceptualization, Data curation, Formal analysis, Investigation, Methodology, Software, Writing – original draft, Writing – review & editing. **Yuqing Wei:** Conceptualization, Formal analysis, Funding acquisition, Methodology, Software. **Hao Xu:** Data curation, Formal analysis, Methodology, Software, Supervision. **Zhangmin Ke:** Investigation, Methodology, Software, Visualization. **He Zhang:** Funding acquisition, Software, Supervision. **Xinyu Zhou:** Formal analysis, Resources, Software. **Peilin Chen:** Resources, Software. **Ping Zhan:** Conceptualization, Methodology, Supervision. **Fang Zhang:** Methodology, Software, Supervision. **Suhua Zhu:** Formal analysis, Resources, Supervision. **Jiajia Jin:** Conceptualization, Funding acquisition, Methodology, Software, Supervision, Writing – review & editing. **Tangfeng Lv:** Conceptualization, Funding acquisition, Methodology, Project administration, Supervision, Writing – review & editing. **Yong Song:** Funding acquisition, Methodology, Supervision, Writing – review & editing.

## Declaration of competing interest

The authors declare that they have no conflict of interest.
