# Supplementary material for: SIRT7-mediated desuccinylation of FOXO4 suppresses ferroptosis to alleviate LPS-induced acute lung injury
Source: Redox Biol. 2026 Jul 18;95:104315. doi: 10.1016/j.redox.2026.104315 (PMC13393011; doi:10.1016/j.redox.2026.104315)
Supplement: Multimedia component 1 [file mmc1.docx]

Supplementary Material

**SIRT7-Mediated Desuccinylation of FOXO4 Suppresses Ferroptosis to Alleviate LPS-Induced Acute Lung Injury**

Kaikai Shen^1#^, Yuqing Wei^2#^, Hao Xu^3#^, Zhangmin Ke^1,4^, He Zhang^2^, Xinyu Zhou^1^, Peilin Chen^1^, Ping Zhan^1^, Fang Zhang^1^, Suhua Zhu^1^, Jiajia Jin^1*^, Tangfeng Lv^1*^, Yong Song^1*^

1 Department of Respiratory and Critical Care Medicine, Jinling Hospital, Affiliated Hospital of Medical School, Nanjing University, Nanjing 210002, China.

2 Department of Respiratory and Critical Care Medicine, The First Affiliated Hospital of Wannan Medical University (Yijishan Hospital of Wannan Medical University), Wuhu 241000, China.

3 Department of Respiratory and Critical Care Medicine, The People’s Hospital of Danyang, Affiliated Danyang Hospital of Nantong University, Danyang 212300, China.

4 Department of Respiratory and Critical Care Medicine, Afﬁliated Jiangning Hospital of Nanjing Medicine University, Nanjing 210002, China.

# This author contributed equally to this work.

* Corresponding author

Jiajia Jin, Ph.D. E-mail: [jiajialmf@foxmail.com](mailto:jiajialmf@foxmail.com)

Tangfeng Lv, Ph.D. E-mail: [TangfengLv7210@nju.edu.cn](mailto:TangfengLv7210@nju.edu.cn)

Yong Song, Ph.D. E-mail: [yong.song@nju.edu.cn](mailto:yong.song@nju.edu.cn%20)

The authors declare no competing financial interests.


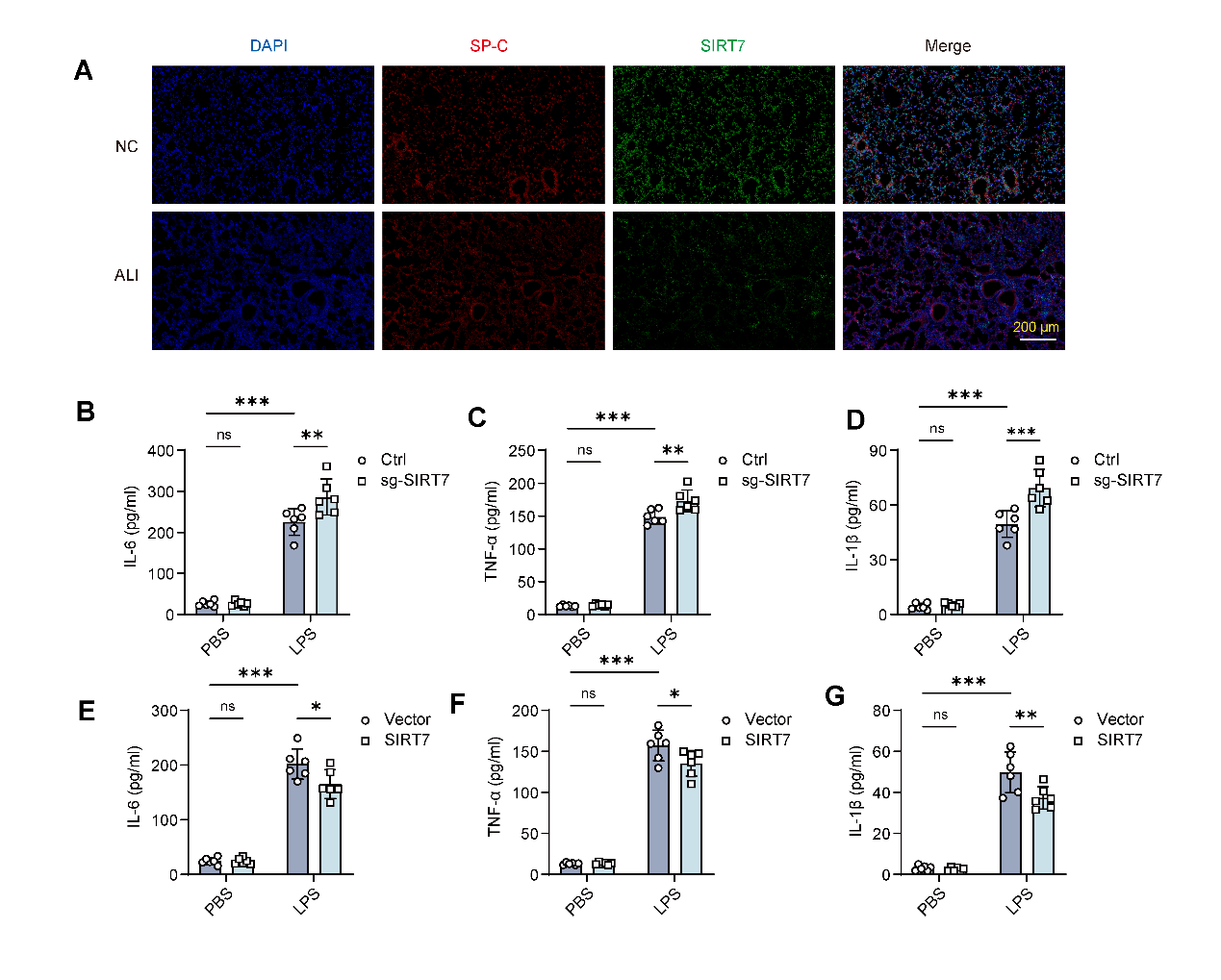


**Fig. S1 Immunofluorescence demonstrates SIRT7 and SP-C co-localization in lung tissues from NC and ALI mice, while analysis of cell culture supernatants reveals altered expression of pro-inflammatory cytokines upon SIRT7-KO or overexpression in MLE-12 cells. A** Immunofluorescence staining for SIRT7 (green) and SP-C (red) in lung tissues from control and ALI mice (n = 3, scale bar = 200 μm). **B-D** Expression of pro-inflammatory cytokines (IL-6, TNF-α and IL-1β) in supernatants from SIRT7-KO MLE-12 cells (n = 6). Two-way ANOVA followed by Tukey’s post-hoc test for multiple comparisons. **E-G** Expression of pro-inflammatory cytokines (IL-6, TNF-α and IL-1β) in supernatants from SIRT7-OE MLE-12 cells (n = 6). Two-way ANOVA followed by Tukey’s post-hoc test for multiple comparisons. LPS was applied at a concentration of 10 μg/mL. Data are presented as mean ± SD. Each experiment was conducted with three or six independent biological replicates. **p* < 0.05, ***p* < 0.01, and ****p* < 0.001; ns, not signiﬁcant.


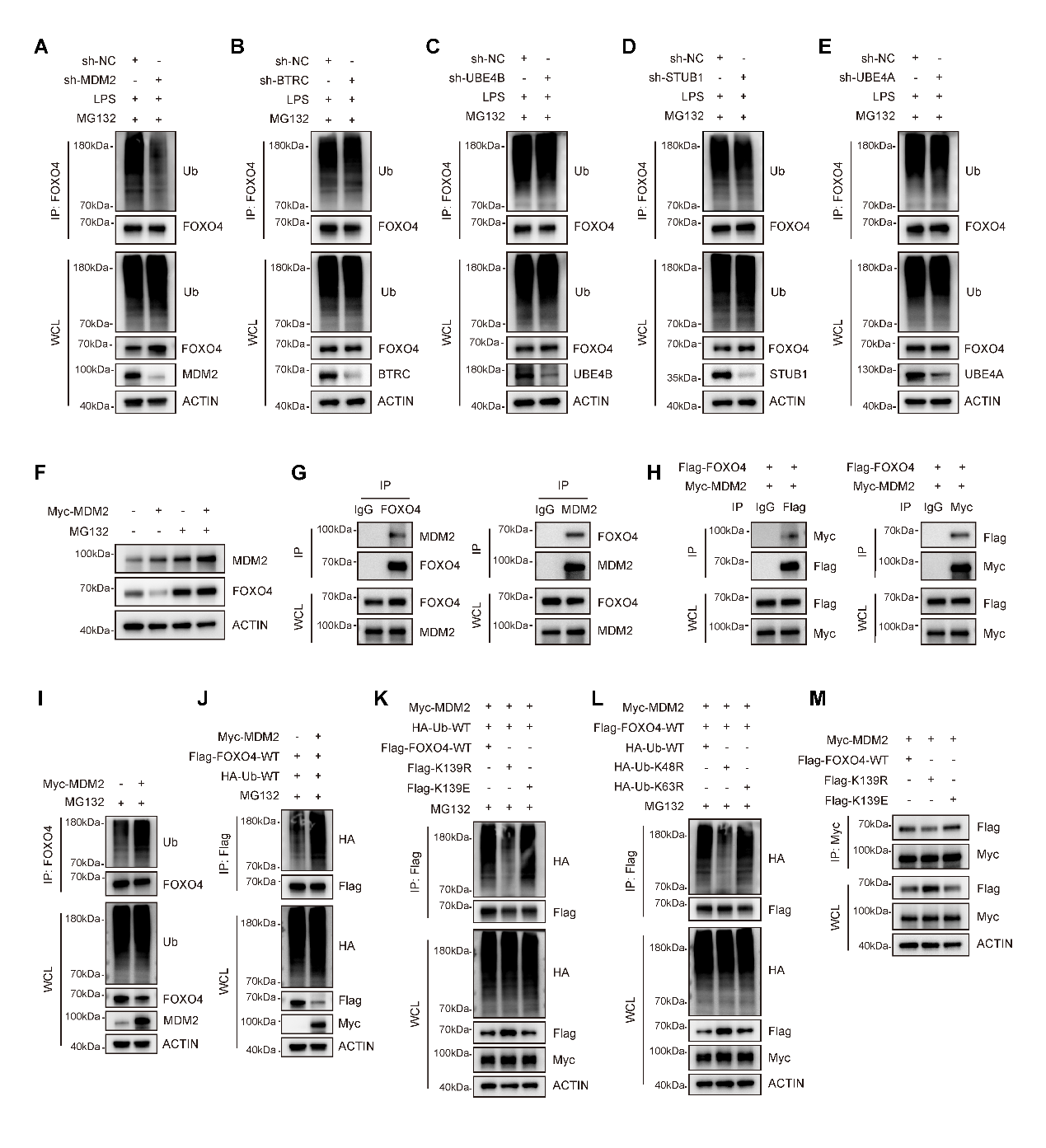


**Fig. S2 K139 succinylation enhances MDM2-mediated K48-linked ubiquitination and subsequent proteasomal degradation of FOXO4. A-E** Co-IP analysis of FOXO4 ubiquitination in MLE-12 cells following knockdown of MDM2, BTRC, UBE4B, STUB1, or UBE4A. Cells were pretreated with LPS (10 μg/mL, 24 h) and MG132 (20 μM, 8 h). **F** Western blot analysis of FOXO4 protein levels in MLE-12 cells following MDM2-OE and MG132 (20 μM, 8 h) pretreatment. **G** Co-IP analysis of the endogenous MDM2-FOXO4 interaction in MLE-12 cell lysates. **H** Co-IP analysis of the interaction between Myc-tagged MDM2 and Flag-tagged FOXO4 in co-transfected HEK293T cells. **I** Co-IP analysis of FOXO4 ubiquitination in MLE-12 cells following MDM2-OE and MG132 pretreatment (20 μM, 8 h). **J** Co-IP analysis of FOXO4 ubiquitination in MLE-12 cells after transfection with Myc-tagged MDM2, Flag-tagged FOXO4, and HA-tagged Ub-WT plasmids following MG132 pretreatment (20 μM, 8 h). **K-L** Co-IP analysis of FOXO4 ubiquitination in MLE-12 cells following transfection with indicated plasmids and MG132 pretreatment (20 μM, 8 h). **M** Co-IP analysis the interaction between MDM2 and FOXO4 following transfection with indicated plasmids. The data shown are representative of at least three independent biological experiments.


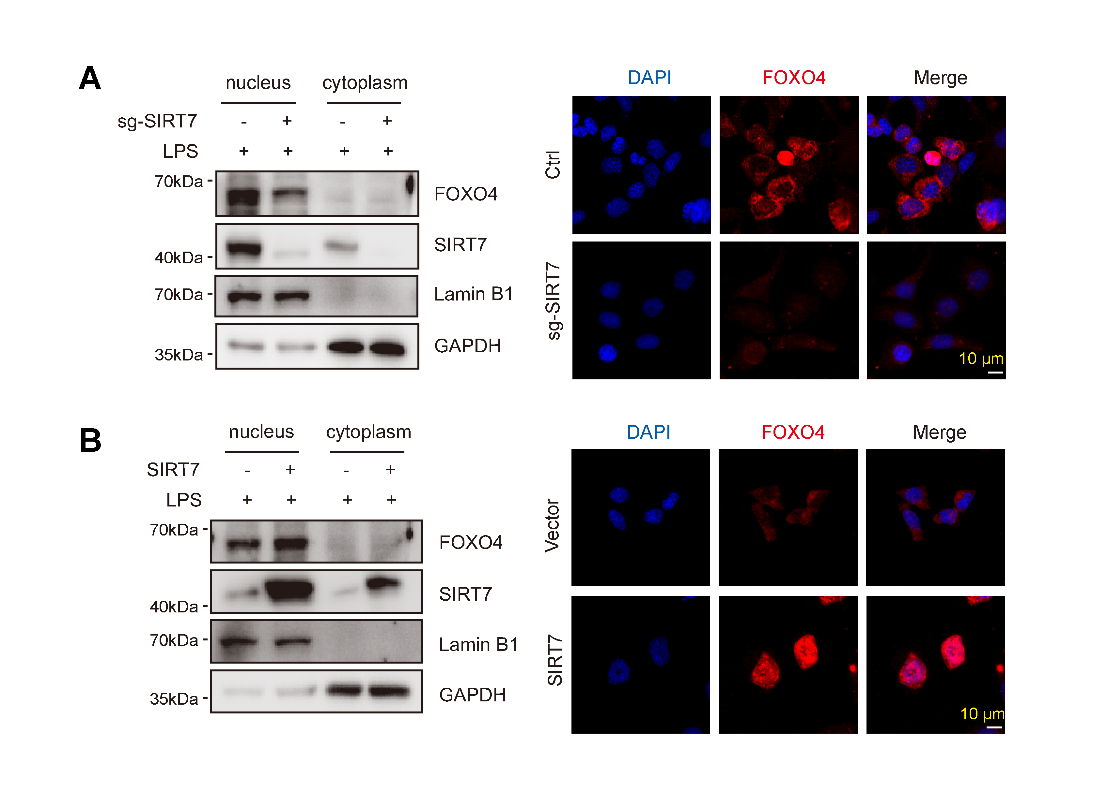


**Fig. S3 SIRT7 facilitates the nuclear retention of FOXO4 in MLE-12 cells. A** Western blotting quantification of FOXO4 protein levels in cytoplasmic and nuclear fractions from SIRT7-KO MLE-12 cells. Immunofluorescence was used to assess FOXO4 subcellular localization (red) in SIRT7-KO MLE-12 cells (scale bar = 10 μm). **B** Western blotting quantification of FOXO4 protein levels in cytoplasmic and nuclear fractions from SIRT7-OE MLE-12 cells. Immunofluorescence was used to assess FOXO4 subcellular localization (red) in SIRT7-OE MLE-12 cells (scale bar = 10 μm). LPS was applied at a concentration of 10 μg/mL. The data shown are representative of at least three independent biological experiments.


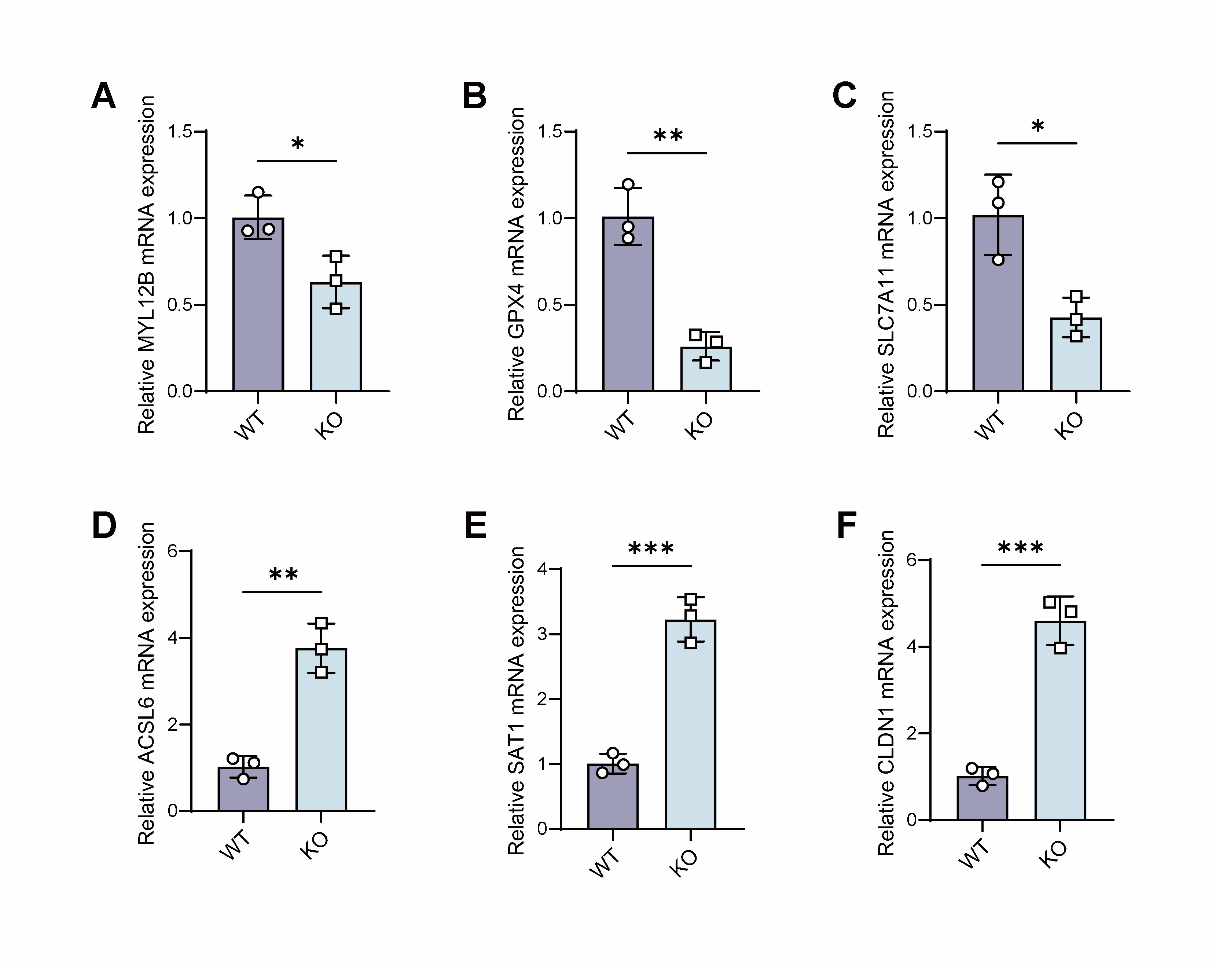


**Fig. S4** qRT-PCR validation of the six most DEGs ranked by absolute log_2_FC. **A-F** Relative mRNA expression levels of MYL12B, GPX4, SLC7A11, ACSL6, SAT1, and CLDN1 in lung tissue from WT and KO mice (n = 3). Student’s t-test. Data are presented as mean ± SD. Each experiment was conducted with three independent biological replicates. **p* < 0.05, ***p* < 0.01, and ****p* < 0.001.


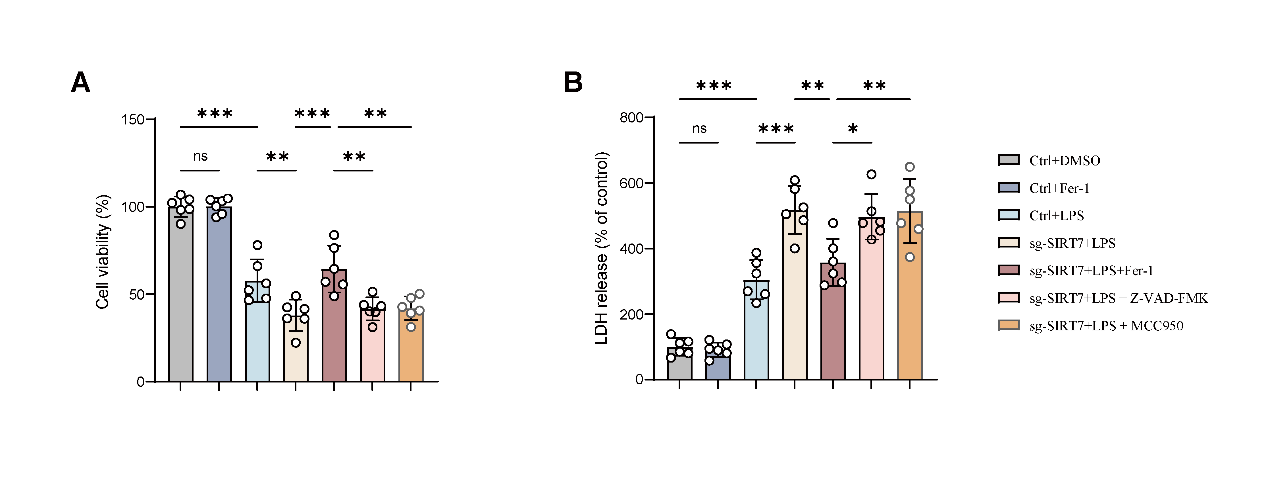


**Fig. S5** **Pharmacological evidence indicates that LPS-induced cell death in SIRT7-KO MLE-12 cells is ferroptosis, not apoptosis or pyroptosis. A** SIRT7-KO MLE-12 cells were treated with LPS (10 μg/mL, 24 h) alone or in combination with Fer-1 (10 μM), Z-VAD-FMK (20 μM), or MCC950 (10 μM). Cell viability was assessed by CCK‑8 assay (n = 6). One-way ANOVA followed by Tukey’s post-hoc test for multiple comparisons. **B** LDH release in the same experimental groups (n = 6). One-way ANOVA followed by Tukey’s post-hoc test for multiple comparisons. Data are presented as mean ± SD. Each experiment was conducted with six independent biological replicates. **p* < 0.05, ***p* < 0.01, and ****p* < 0.001; ns, not signiﬁcant.


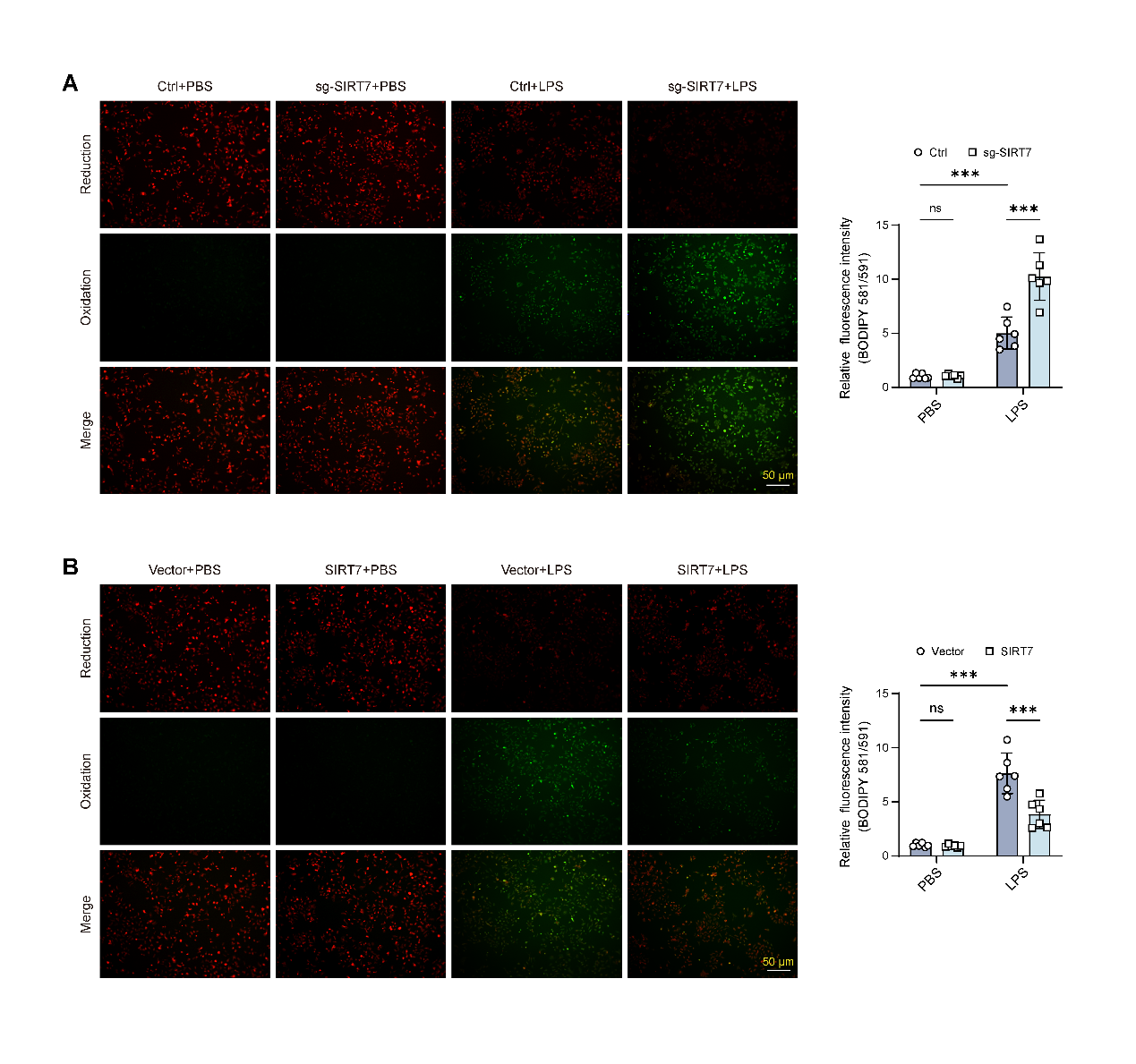


**Fig. S6 Detection of lipid peroxidation levels. A** Lipid ROS following SIRT7-KO in MLE-12 cells detected using the C11-BODIPY 581/591 fluorescent probe (n = 6). Two-way ANOVA followed by Tukey’s post-hoc test for multiple comparisons. **B** Lipid ROS following SIRT7-OE in MLE-12 cells detected using the C11-BODIPY 581/591 fluorescent probe (n = 6). Two-way ANOVA followed by Tukey’s post-hoc test for multiple comparisons. Data are presented as mean ± SD. Each experiment was conducted with six independent biological replicates. **p* < 0.05, ***p* < 0.01, and ****p* < 0.001; ns, not signiﬁcant.

**Table S1 Clinical characteristics of ARDS patients versus those of controls.**

| **Characteristic** | **Controls (n = 20)** | **ARDS (n = 45)** | **t/Z/χ2** | ***p* value** |
| --- | --- | --- | --- | --- |
| **Demographics** |  |  |  |  |
| Age, years (mean ± SD) | 65.30 ± 10.62 | 70.38 ± 11.58 | -1.672 | 0.100 |
| Male sex, n (%) | 11 (55.0) | 27 (60.0) | 0.143 | 0.706 |
| **Behavioral history** |  |  |  |  |
| Smoking status, n (%) | 10 (50.0) | 19 (42.2) | 0.339 | 0.560 |
| Alcohol use, n (%) | 10 (50.0) | 18 (40.0) | 0.565 | 0.452 |
| **Comorbidities, n (%)** |  |  |  |  |
| Hypertension | 11 (55.0) | 28 (62.2) | 0.301 | 0.583 |
| Diabetes mellitus | 7 (35.0) | 21 (46.7) | 0.769 | 0.381 |
| **Laboratory tests, Median (IQR)** |  |  |  |  |
| WBC count, ×10⁹/L | 6.85 (5.93, 8.03) | 15.60 (10.96, 18.46) | -6.155 | < 0.001 |
| Hemoglobin, g/L | 135.00 (126.50, 146.50) | 90.00 (76.00, 115.50) | -5.232 | < 0.001 |
| Platelet count, ×10⁹/L | 220.50 (178.50, 248.75) | 174.00 (108.50, 306.00) | -1.066 | 0.286 |
| NLR | 4.25 (3.43, 6.18) | 14.40 (9.70, 26.50) | -5.721 | < 0.001 |
| CRP, mg/L | 2.20 (0.95, 4.60) | 92.10 (46.40, 194.00) | -6.233 | < 0.001 |
| PCT, ng/mL | 0.10 (0.04, 0.30) | 0.50 (0.13, 4.36) | -3.484 | < 0.001 |
| **Disease severity score, Median (IQR)** |  |  |  |  |
| APACHE II scores | / | 23.00 (14.00, 30.50) | / | / |
| **SIRT7 mRNA level, Median (IQR)** | 1.03 (0.55, 1.72) | 0.43 (0.19, 0.75) | -3.681 | < 0.001 |
| **Berlin categories, n (%)** |  |  |  |  |
| Mild | / | 11 (24.4) | / | / |
| Moderate | / | 12 (26.7) | / | / |
| Severe | / | 22 (48.9) | / | / |

**Table S2 Primers for SIRT7-KO mice qPCR detection.**

| **PCR NO.** | **Primer name** | **Primer sequence (5' to 3')** | **Band Szie (bp)** |
| --- | --- | --- | --- |
| PCR1 | SIRT7-5WT-tF1 | AAGCGTGAAGACCCGAGTTTG | WT: 4013; KO: 272 |
|  | SIRT7-3WT-tR1 | CATCAACAATGTGGCACAGGAG |  |
| PCR2 | SIRT7-WT-tF1 | GCTGTGGTATTCAGGTTCCATTC | WT: 389; KO: 0 |
|  | SIRT7-WT-tR1 | CGGCAGTGGAGTTATTCTAAGC |  |

**Table S3** **sgRNA and shRNA sequences used to generate SIRT7-KO and the respective gene‑knockdown MLE‑12 cell lines**

|  | **Sequence** | **Supplier** |
| --- | --- | --- |
| Control | CACCGGGTTCTCCGAACGTGTCACGT | Tsingke Biotechnology |
| sg-SIRT7-1 | CGAGCGGCTCAGACCGCCAC | Tsingke Biotechnology |
| sg-SIRT7-2 | GGTCGAAGTCGGCGGCGTGA | Tsingke Biotechnology |
| sg-SIRT7-3 | AAGCTGCTGAGCGGGTCCGG | Tsingke Biotechnology |
| sh-NC | ACTACCGTTGTTATAGGTGT | Tsingke Biotechnology |
| sh-FOXO4 | GCACGGTGCCCTACTTCAAGG | Tsingke Biotechnology |
| sh-MDM2 | GCATCAGGATCTTGACGAT | Tsingke Biotechnology |
| sh-BTRC | GCCAGGCTTTGCATAAACCAA | Tsingke Biotechnology |
| sh-UBE4A | GCTCGATTATTGCTTCAAGAT | Tsingke Biotechnology |
| sh-UBE4B | CGCTATCACATTAGCACTATT | Tsingke Biotechnology |
| sh-STUB1 | GAGAGTTATGATGAGGCCATT | Tsingke Biotechnology |

**Table S4 Recombinant DNA in this manuscript.**

| **Recombinant DNA** | **Supplier** |
| --- | --- |
| pcDNA3.1-Flag-FOXO4-WT | Tsingke Biotechnology |
| pcDNA3.1-Flag-FOXO4-K139R | Tsingke Biotechnology |
| pcDNA3.1-Flag-FOXO4-K139E | Tsingke Biotechnology |
| pcDNA3.1-Flag-FOXO4-K151R | Tsingke Biotechnology |
| pcDNA3.1-Flag-FOXO4-K213R | Tsingke Biotechnology |
| pcDNA3.1-FOXO4 | Tsingke Biotechnology |
| pcDNA3.1-HA-Ub-WT | Tsingke Biotechnology |
| pcDNA3.1-HA-Ub-K48R | Tsingke Biotechnology |
| pcDNA3.1-HA-Ub-K63R | Tsingke Biotechnology |
| pcDNA3.1-HA-SIRT7 | Tsingke Biotechnology |
| pcDNA3.1-SIRT7 | Tsingke Biotechnology |
| pcDNA3.1 control | Tsingke Biotechnology |
| pcDNA3.1-Myc-MDM2 | Tsingke Biotechnology |
| pGL3-basic GPX4 promoter-WT | Tsingke Biotechnology |
| pGL3-basic GPX4 promoter-MUT1 | Tsingke Biotechnology |
| pGL3-basic GPX4 promoter-MUT2 | Tsingke Biotechnology |
| pGL3-basic GPX4 promoter-MUT3 | Tsingke Biotechnology |

**Table S5 Primers for qPCR detection.**

| **Primer name** | **Primer sequence (5' to 3')** |
| --- | --- |
| m-SIRT7-F | GCACTTGGTTGTCTACACGG |
| m-SIRT7-R | TGTCCATACTCCATTAGGACCC |
| h-SIRT7-F | GACCTGGTAACGGAGCTGC |
| h-SIRT7-R | CGACCAAGTATTTGGCGTTCC |
| m-SIRT5-F | CCAGTTGTGTTGTAGACGAAAGC |
| m-SIRT5-R | TTCCGAAAGTCTGCCATATTTGA |
| m-CPT1A-F | TGGCATCATCACTGGTGTGTT |
| m-CPT1A-R | GTCTAGGGTCCGATTGATCTTTG |
| m-P300-F | AGCCAAGCGGCCTAAACTC |
| m-P300-R | CGCCACCATTGGTTAGTCCC |
| m-KAT2A-F | AACCTGAGCGAGTTGTGCC |
| m-KAT2A-R | GCCGGTTAATCTCGTCCTCTG |
| m-HAT1-F | ACACCAACACAGCAATCGAG |
| m-HAT1-R | TGTAACCGAAAGCAGTTTCATCA |
| m-OXCT1-F | GCCCTGCATAAGGGGTGTG |
| m-OXCT1-R | GCAAGGTTGCACCATTAGGAAT |
| m-SAT1-F | GAGAACACCCCTTCTACCACT |
| m-SAT1-R | GCCTCTGTAATCACTCATCACGA |
| m-ACSL6-F | AAGTGACAGAGAGTCAGTGGG |
| m-ACSL6-R | TAGGGCGGAGAGCCTTCAT |
| m-CLDN1-F | TGCCCCAGTGGAAGATTTACT |
| m-CLDN1-R | CTTTGCGAAACGCAGGACAT |
| m-SLC7A11-F | GGCACCGTCATCGGATCAG |
| m-SLC7A11-R | CTCCACAGGCAGACCAGAAAA |
| m-GPX4-F | TGTGCATCCCGCGATGATT |
| m-GPX4-R | CCCTGTACTTATCCAGGCAGA |
| m-MYL12B-F | TGGGGGATCGGTTTACAGATG |
| m-MYL12B-R | TTTCAGGATGCGTGTGAACTC |
| m-Beta actin-F | GGCTGTATTCCCCTCCATCG |
| m-Beta actin-R | CCAGTTGGTAACAATGCCATGT |
| h-Beta actin-F | ACCTTCTACAATGAGCTGCG |
| h-Beta actin-R | CCTGGATAGCAACGTACATGG |

**Table S6 Antibody information and application details.**

| **Antibody** | **Supplier** | **Cat No.** | **Concentration** |
| --- | --- | --- | --- |
| Anti-SIRT7 | Proteintech | 12994-1-AP | 1:2000 for WB; 1:50 for IF |
| Anti-SIRT7 | Santa Cruz | sc-365344 | 4 μg for IP |
| Anti-FOXO4 | Abcam | ab128908 | 1:1000 for WB |
| Anti-FOXO4 | Santa Cruz | sc-373877 | 4 μg for IP; 1:100 for IF; 4 μg for ChIP |
| Anti-MDM2 | Abcam | ab259265 | 1:1000 for WB |
| Anti-MDM2 | Santa Cruz | sc-965 | 4 μg for IP |
| Anti-STUB1 | Selleck | F1053 | 1:10000 for WB |
| Anti-TRCP | Selleck | F0415 | 1:1000 for WB |
| Anti-UBE4A | Proteintech | 21548-1-AP | 1:2000 for WB |
| Anti-UBE4B | CST | 17673 | 1:1000 for WB |
| Anti-Pan-Acetyllysine | PTM Biolabs | PTM-105RM | 1:1000 for WB |
| Anti-Pan-Lactyllysine | PTM Biolabs | PTM-1401RM | 1:1000 for WB |
| Anti-Pan-Crotonyllysine | PTM Biolabs | PTM-501 | 1:1000 for WB |
| Anti-Pan-succinyllysine | PTM Biolabs | PTM-401 | 1:1000 for WB |
| Anti-Pan-succinyllysine | PTM Biolabs | PTM-419 | 4 μg for IP |
| Anti-Flag Tag | CST | 8146 | 1:50 for IP |
| Anti-Flag Tag | CST | 14793 | 1:1000 for WB; 1:500 for IF |
| Anti-HA Tag | Proteintech | 66006-2-Ig | 4 μg for IP; 1:500 for IF |
| Anti-HA Tag | Proteintech | 51064-2-AP | 1:5000 for WB |
| Anti-Myc Tag | CST | 2276 | 1:250 for IP |
| Anti-Myc Tag | CST | 2278 | 1:1000 for WB |
| Anti-Ubiquitin | Proteintech | 10201-2-AP | 1:2000 for WB |
| Anti-GAPDH | Proteintech | 60004-1-Ig | 1:100000 for WB |
| Anti-Beta Actin | Proteintech | 66009-1-Ig | 1:20000 for WB |
| Anti-Lamin B1 | Proteintech | 12987-1-AP | 1:10000 for WB |
| Anti-PTGS2 | Proteintech | 27308-1-AP | 1:500 for WB |
| Anti-Prosurfactant Protein C | Proteintech | 10774-1-AP | 1:50 for IF |
| Anti-GPX4 | Abcam | ab125066 | 1:1000 for WB |
| Anti-SLC7A11(xCT) | Affinity Biosciences | DF12509 | 1:1000 for WB |
| Anti-FTH | Abcam | ab75973 | 1:1000 for WB |
| Alexa Fluor® 488-conjugated Goat Anti-Rabbit IgG (H+L) | Servicebio | GB25303 | 1:400 for IF |
| Alexa Fluor® 488-conjugated Goat Anti-Mouse IgG (H+L) | Servicebio | GB25301 | 1:400 for IF |
| Cy3 conjugated Goat Anti-Rabbit IgG (H+L) | Servicebio | GB21303 | 1:300 for IF |
| Cy3 conjugated Goat Anti-mouse IgG(H+L) | Servicebio | GB21301 | 1:300 for IF |
| Goat Anti-Rabbit IgG(H+L) | Proteintech | SA00001-2 | 1:10000 for WB |
| Goat Anti-Mouse IgG(H+L) | Proteintech | SA00001-1 | 1:10000 for WB |
| Anti-Rabbit IgG | Proteintech | 30000-0-AP | 4 μg for IP |
| Anti-Mouse IgG | Proteintech | B900620 | 4 μg for IP; 4μg for ChIP |
| Anti-Mouse CD16/CD32 | BD Biosciences | 553141 | 1.0 μg/million cells for FCM |
| Anti-PE-F4/80 | BioLegend | 123110 | 1.0 μg/million cells for FCM |
| Anti-APC-Ly6G | BioLegend | 127614 | 0.06 μg/million cells for FCM |

**Table S7 Primers for ChIP-qPCR detection.**

| **Primer name** | **Primer sequence (5' to 3')** | **Band Szie (bp)** |
| --- | --- | --- |
| GPX4 site1 F | GACAGCCAGGTCTACACAGA | 136 |
| GPX4 site1 R | CTTCGGAAGAGCAGTCGGG |  |
| GPX4 site2 F | AGGAGATCTGACGCCCTCTT | 153 |
| GPX4 site2 R | TCTCCTGTGAACTTGAACAAACT |  |
| GPX4 site3 F | TCCGCATGTACAGTCACAGG | 143 |
| GPX4 site3 R | GCTGGCCTGGAACTTGCTAT |  |

**Table S8 Top 10 most abundant proteins identified by MS.**

| **No.** | **Gene name** | **Description** | **Abundance** |
| --- | --- | --- | --- |
| 1 | Sirt7 | NAD-dependent protein deacetylase sirtuin-7 | 15800000000 |
| 2 | Lamb1 | Laminin subunit beta-1 | 1990000000 |
| 3 | Ighg2a | Immunoglobulin heavy constant gamma 2a | 1980000000 |
| 4 | Rtcb | RNA-splicing ligase Rtcb homolog | 1290000000 |
| 5 | Foxo4 | Forkhead box protein O4 | 1260000000 |
| 6 | Dis3l | DIS3-like exonuclease 1 | 1100000000 |
| 7 | Igkc | Immunoglobulin kappa constant | 911000000 |
| 8 | Zbtb48 | Telomere zinc finger-associated protein | 852000000 |
| 9 | Mrps15 | Small ribosomal subunit protein us15m | 797000000 |
| 10 | Aoah | Acyloxyacyl hydrolase | 769000000 |

**Table S9 The succinylation modification sites of FOXO4 predicted by the GPSuc websites.**

| **Sites** | **Score** | **Justification** |
| --- | --- | --- |
| 6 | 0.660 | Non Succinylation |
| 7 | 0.624 | Non Succinylation |
| 57 | 0.605 | Non Succinylation |
| 93 | 0.612 | Non Succinylation |
| 120 | 0.658 | Non Succinylation |
| **139** | **0.712** | **Succinylation** |
| 141 | 0.635 | Non Succinylation |
| **151** | **0.680** | **Succinylation** |
| 163 | 0.618 | Non Succinylation |
| 166 | 0.649 | Non Succinylation |
| 174 | 0.651 | Non Succinylation |
| 186 | 0.658 | Non Succinylation |
| 189 | 0.652 | Non Succinylation |
| 203 | 0.642 | Non Succinylation |
| 210 | 0.655 | Non Succinylation |
| **213** | **0.702** | **Succinylation** |
| 214 | 0.615 | Non Succinylation |
| 215 | 0.602 | Non Succinylation |
| 237 | 0.600 | Non Succinylation |
| 408 | 0.589 | Non Succinylation |
| 448 | 0.618 | Non Succinylation |

**Table S10 Blind quantification of positively stained cells with ferroptosis-typical mitochondrial morphology for Fig. 5G.**

| Group | Fields analyzed (n) | Total cells analyzed (n) | Cells with ferroptosis-typical mitochondria (n) | Positive cells |
| --- | --- | --- | --- | --- |
| WT+PBS | 10 | 36 | 4 | 11.11% |
|  | 10 | 35 | 4 | 11.43% |
|  | 10 | 29 | 3 | 10.34% |
| KO+PBS | 10 | 37 | 5 | 13.51% |
|  | 10 | 31 | 4 | 12.90% |
|  | 10 | 36 | 3 | 8.33% |
| WT+LPS | 10 | 37 | 15 | 40.54% |
|  | 10 | 35 | 16 | 45.71% |
|  | 10 | 36 | 17 | 47.22% |
| KO+LPS | 10 | 30 | 26 | 86.67% |
|  | 10 | 38 | 28 | 73.68% |
|  | 10 | 33 | 27 | 81.82% |

**Table S11 Blind quantification of positively stained cells with ferroptosis-typical mitochondrial morphology for Fig. 6B.**

| Group | Fields analyzed (n) | Total cells analyzed (n) | Cells with ferroptosis-typical mitochondria (n) | Positive cells |
| --- | --- | --- | --- | --- |
| Ctrl+PBS | 10 | 33 | 5 | 15.15% |
|  | 10 | 31 | 4 | 12.90% |
|  | 10 | 36 | 6 | 16.67% |
| sg-SIRT7+PBS | 10 | 35 | 4 | 11.43% |
|  | 10 | 33 | 5 | 15.15% |
|  | 10 | 33 | 6 | 18.18% |
| Ctrl+LPS | 10 | 30 | 21 | 70.00% |
|  | 10 | 36 | 19 | 52.78% |
|  | 10 | 35 | 22 | 62.86% |
| sg-SIRT7+LPS | 10 | 37 | 33 | 89.19% |
|  | 10 | 33 | 26 | 78.79% |
|  | 10 | 32 | 26 | 81.25% |

**Table S12 Blind quantification of positively stained cells with ferroptosis-typical mitochondrial morphology for Fig. 6L.**

| Group | Fields analyzed (n) | Total cells analyzed (n) | Cells with ferroptosis-typical mitochondria (n) | Positive cells |
| --- | --- | --- | --- | --- |
| Vector+PBS | 10 | 34 | 5 | 14.71% |
|  | 10 | 35 | 4 | 11.43% |
|  | 10 | 35 | 5 | 14.29% |
| SIRT7+PBS | 10 | 31 | 3 | 9.68% |
|  | 10 | 34 | 5 | 14.71% |
|  | 10 | 36 | 4 | 11.11% |
| Vector+LPS | 10 | 34 | 22 | 64.71% |
|  | 10 | 35 | 17 | 48.57% |
|  | 10 | 36 | 20 | 55.56% |
| SIRT7+LPS | 10 | 33 | 9 | 27.27% |
|  | 10 | 33 | 12 | 36.36% |
|  | 10 | 35 | 9 | 25.71% |

**Table S13 Blind quantification of positively stained cells with ferroptosis-typical mitochondrial morphology for Fig. 8Q.**

| Group | Fields analyzed (n) | Total cells analyzed (n) | Cells with ferroptosis-typical mitochondria (n) | Positive cells |
| --- | --- | --- | --- | --- |
| GFP | 10 | 36 | 35 | 97.22% |
|  | 10 | 38 | 32 | 84.21% |
|  | 10 | 33 | 32 | 96.97% |
| WT | 10 | 33 | 13 | 39.39% |
|  | 10 | 35 | 17 | 48.57% |
|  | 10 | 32 | 17 | 53.13% |
| K139R | 10 | 30 | 5 | 16.67% |
|  | 10 | 35 | 6 | 17.14% |
|  | 10 | 36 | 8 | 22.22% |
| K139E | 10 | 31 | 14 | 45.16% |
|  | 10 | 37 | 16 | 43.24% |
|  | 10 | 33 | 21 | 63.64% |
