# Supplementary material for: SIRT7-mediated desuccinylation of FOXO4 suppresses ferroptosis to alleviate LPS-induced acute lung injury
Source: Redox Biol. 2026 Jul 18;95:104315. doi: 10.1016/j.redox.2026.104315 (PMC13393011; doi:10.1016/j.redox.2026.104315)

WB original gels

SDS-PAGE band profile of the PageRuler Prestained Protein Ladder

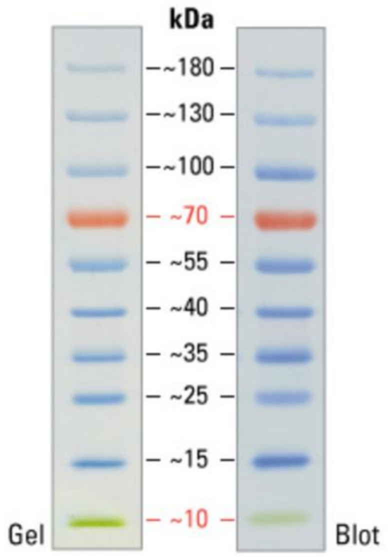

Thermo Scientific Prestained Protein Ladder

Catalog number: 26616

Fig. 1G

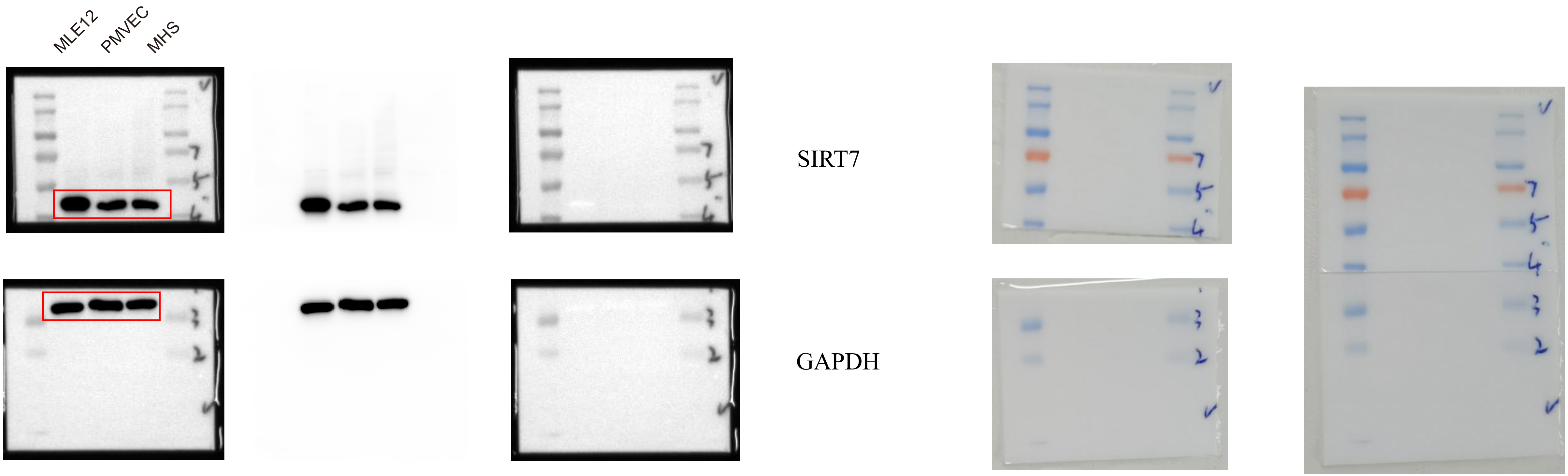

Fig. 1H

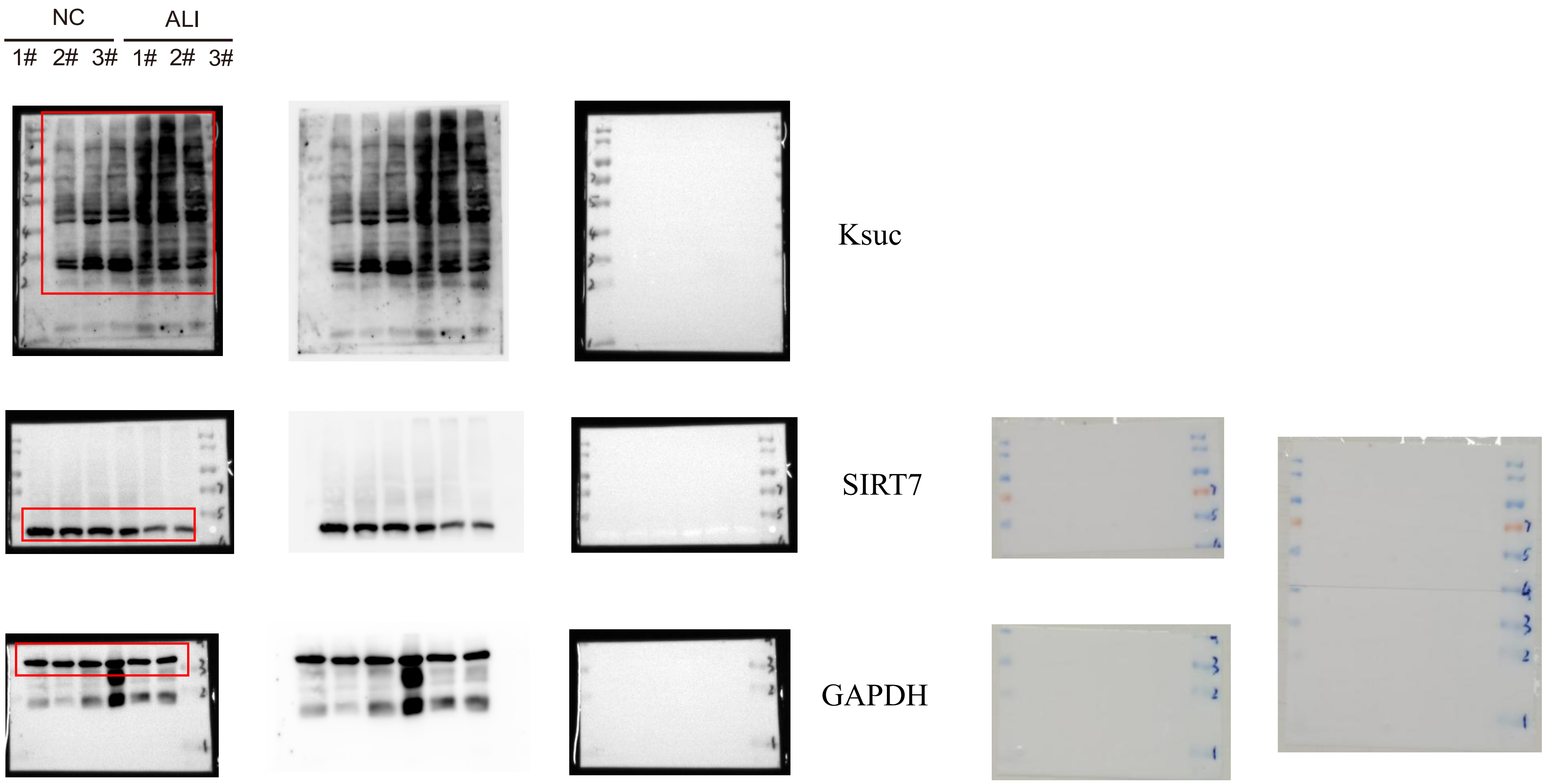

Fig. 1I

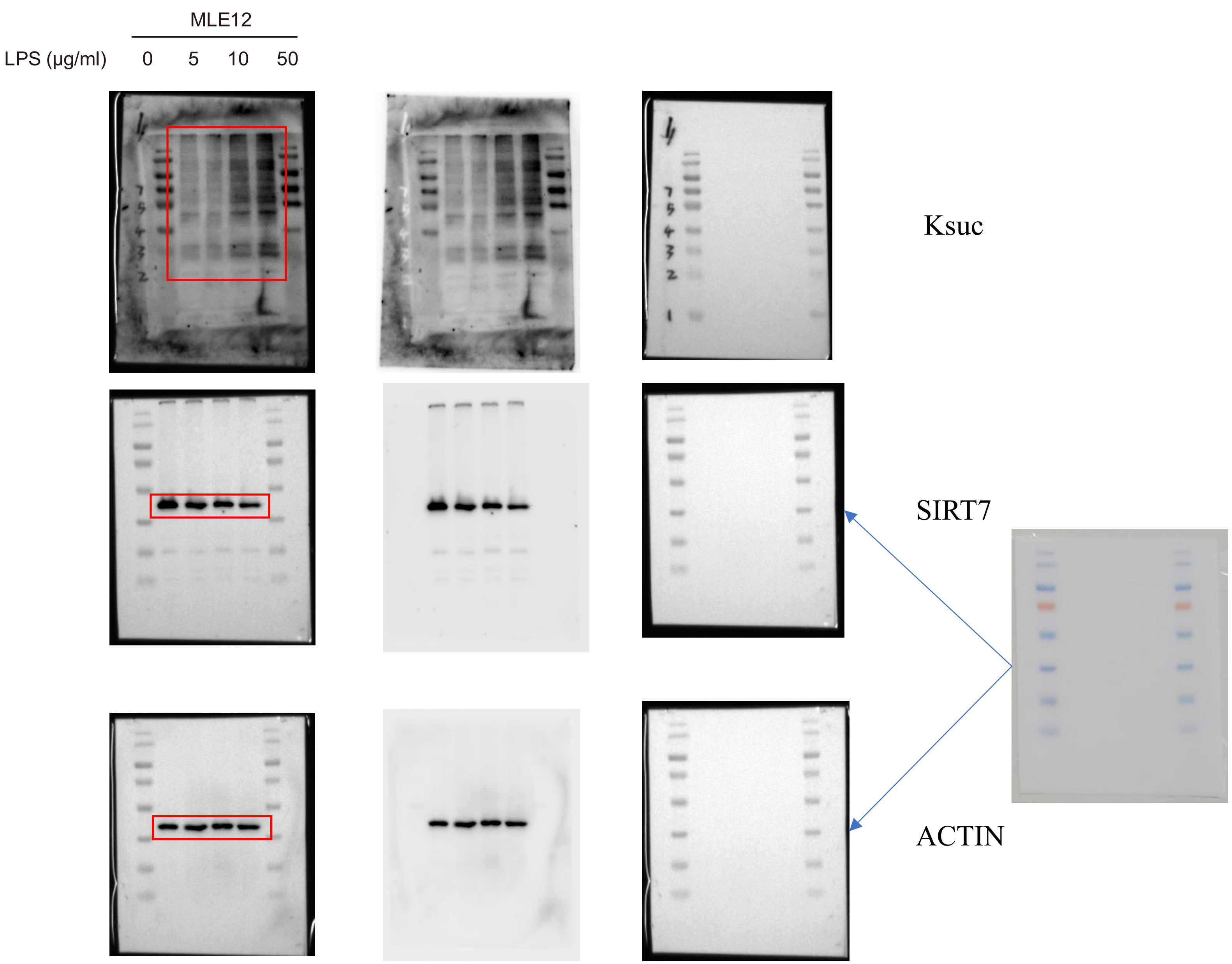

Fig. 1J

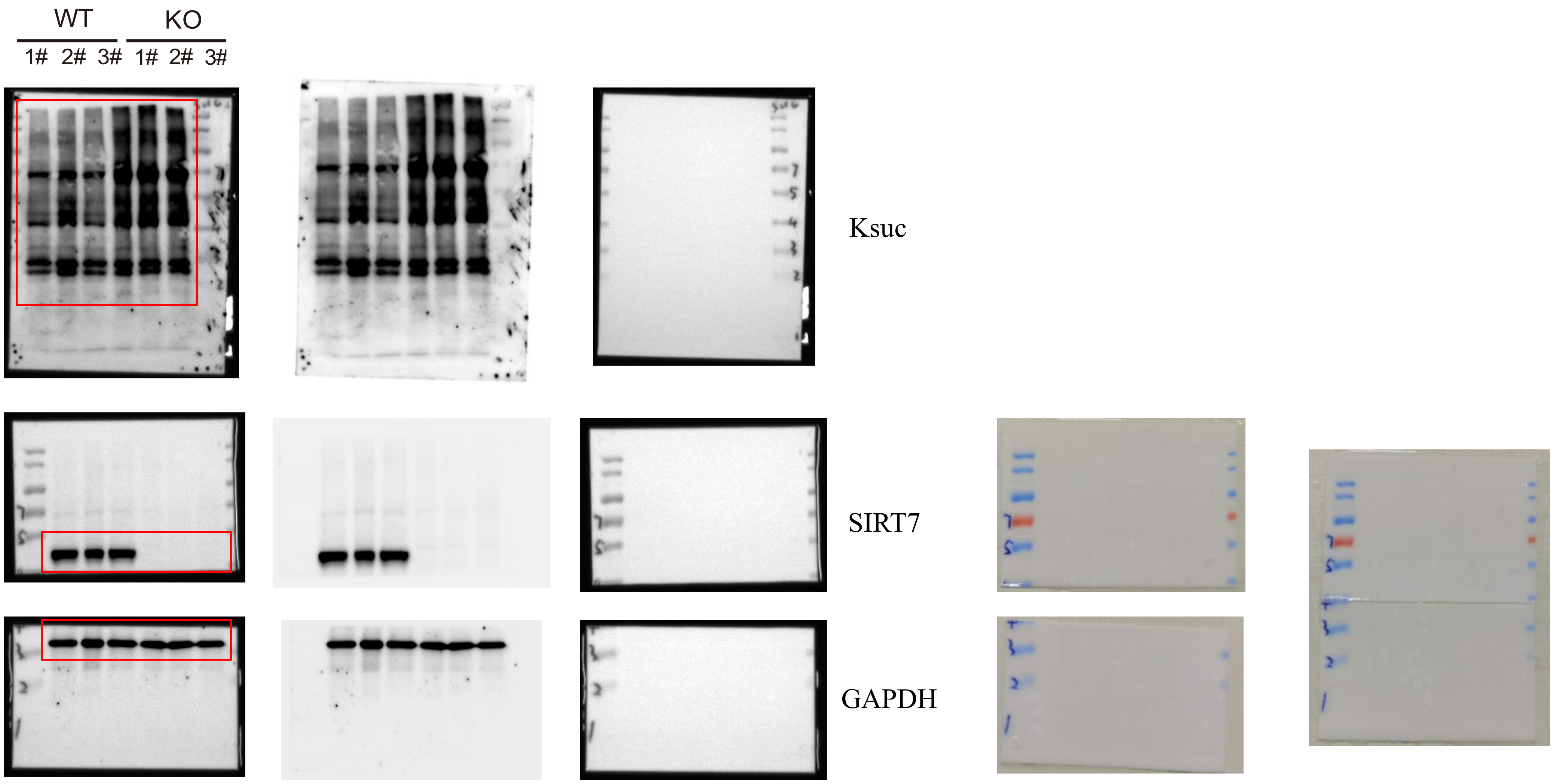

Fig. 1K

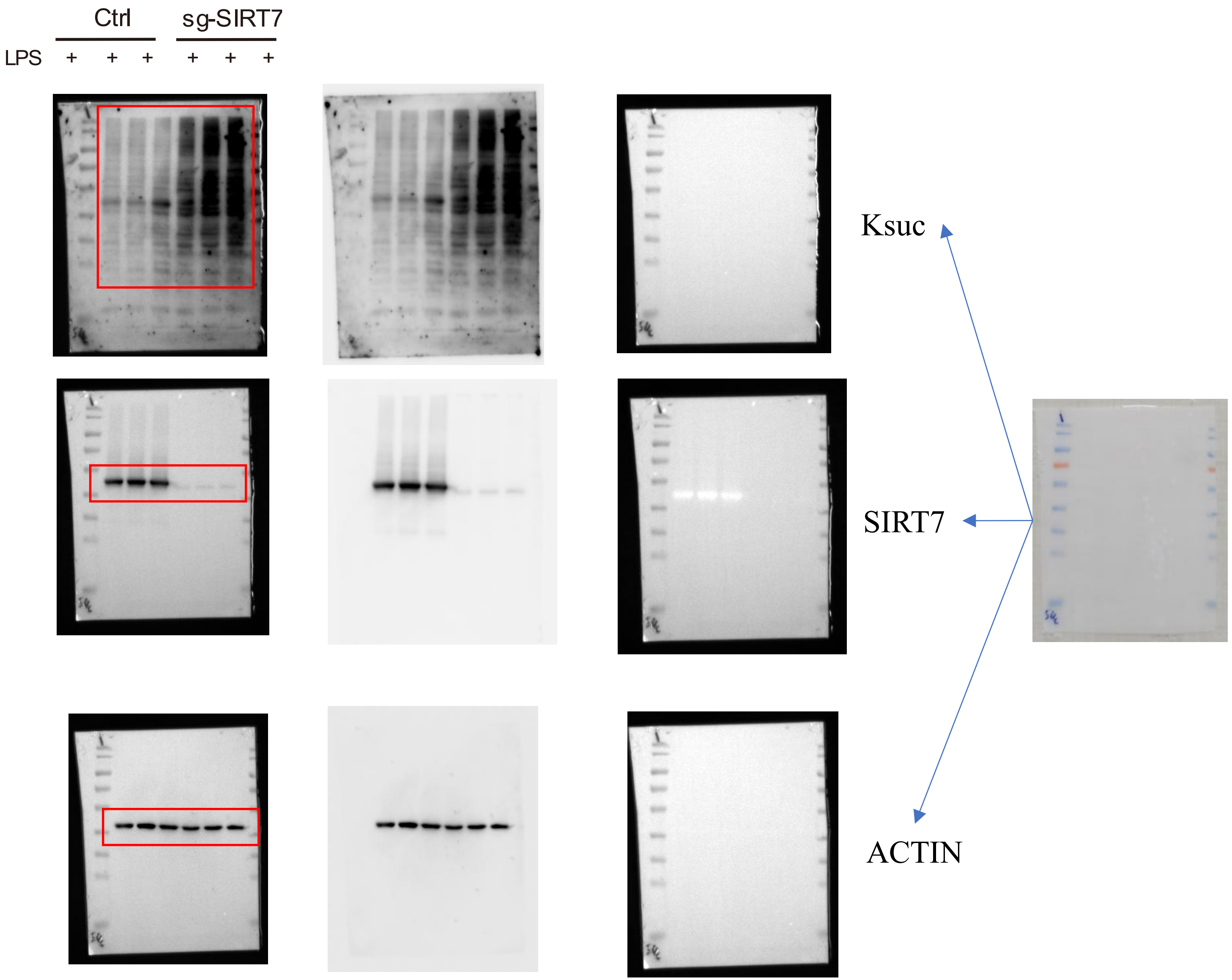

Fig. 1L

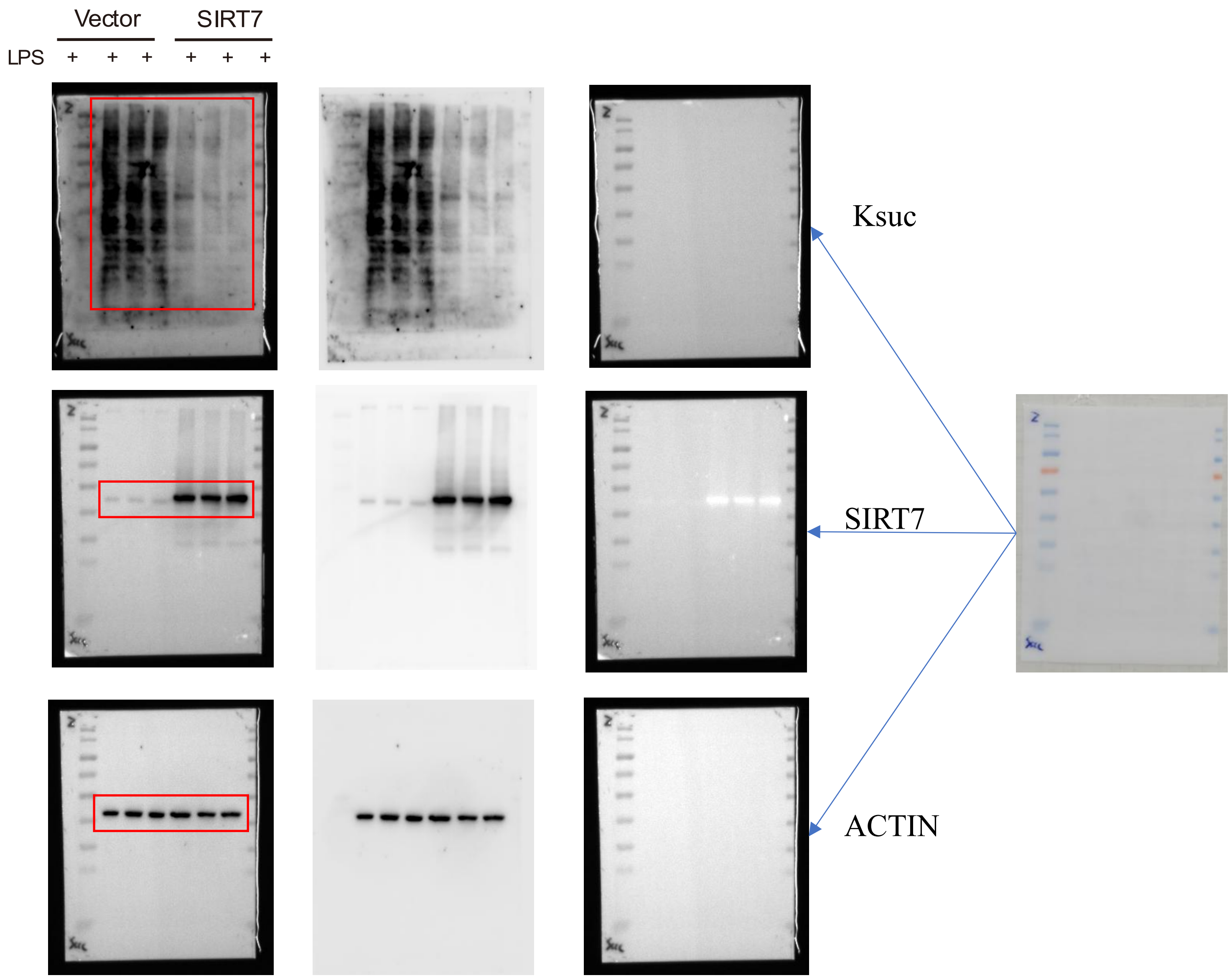

Fig. 2B

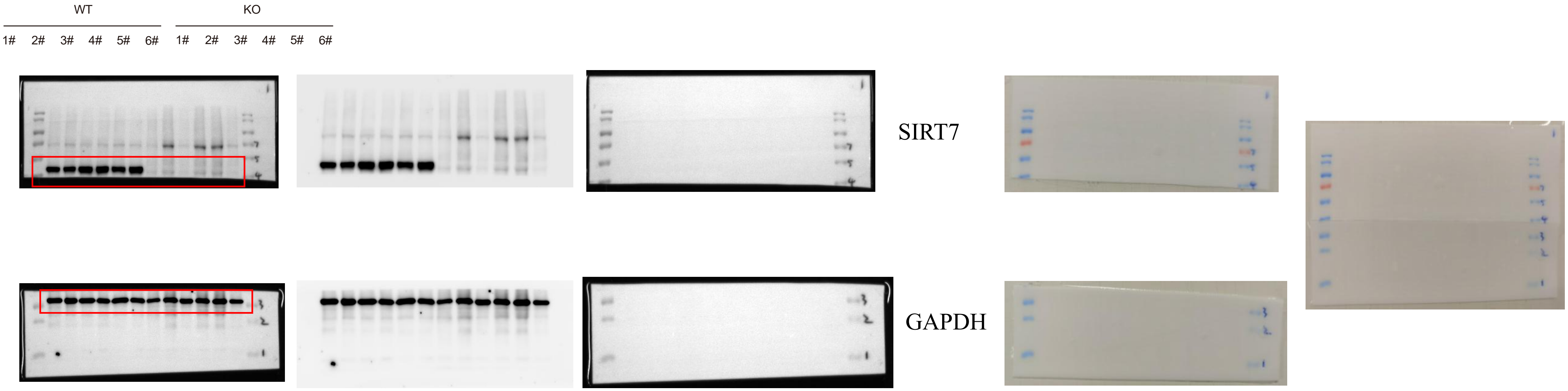

Fig. 3F

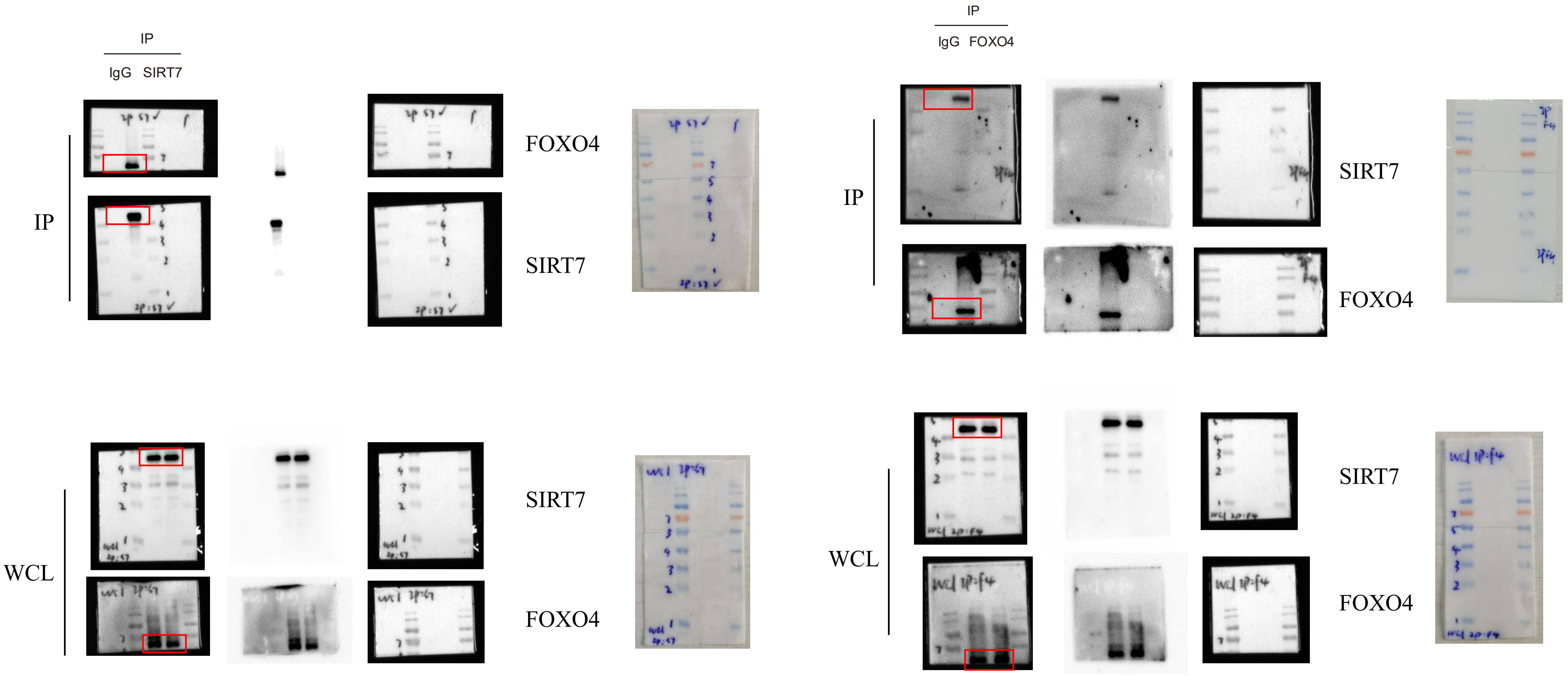
IP
IgG SIRT7
IP
FOXO4
SIRT7
WCL
SIRT7
FOXO4
IP
IgG FOXO4
IP
SIRT7
FOXO4
WCL
SIRT7
FOXO4

Fig. 3G

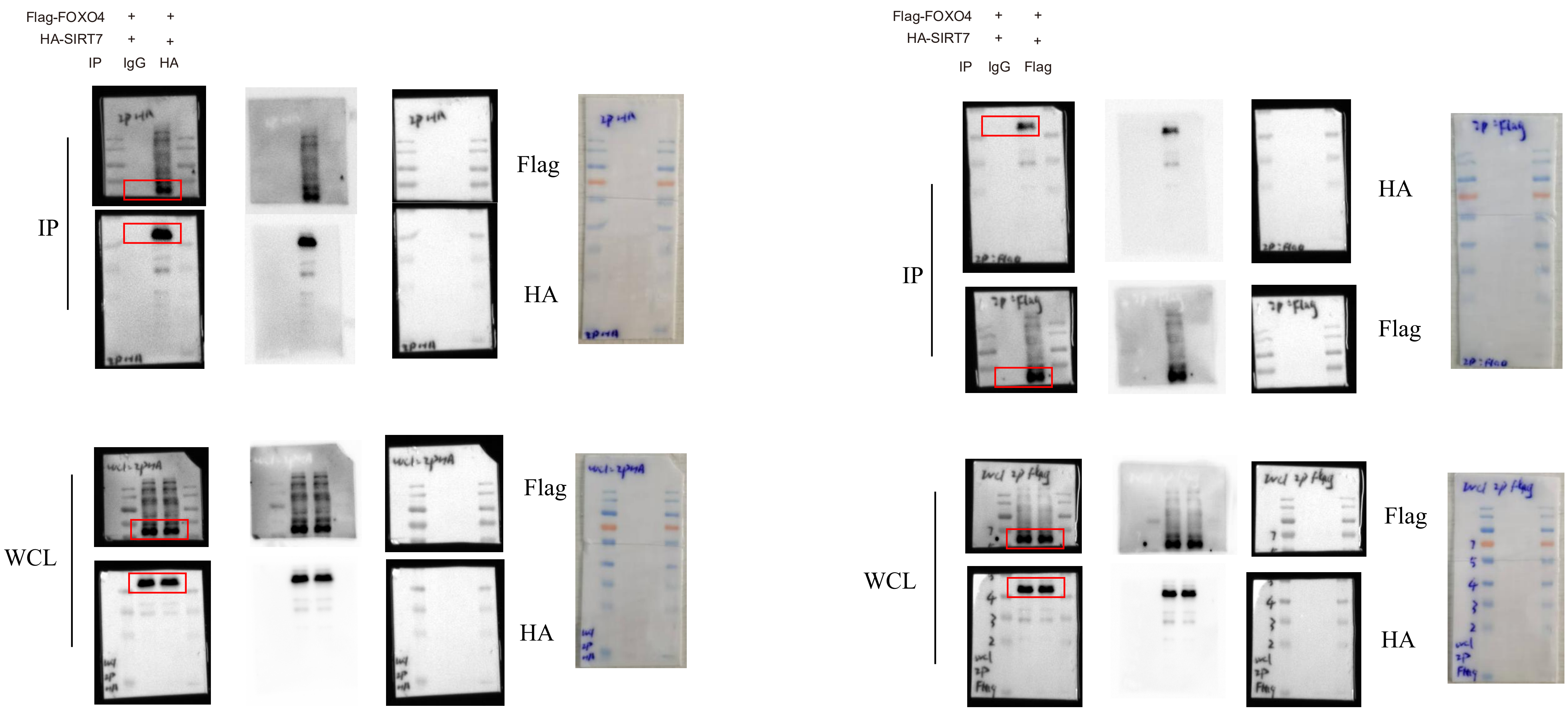
Flag-FOXO4 + +
HA-SIRT7 + +
IP IgG HA
IP
WCL
Flag
HA
Flag
HA
Flag-FOXO4 + +
HA-SIRT7 + +
IP IgG Flag
IP
WCL
HA
Flag
Flag
HA

Fig. 3I

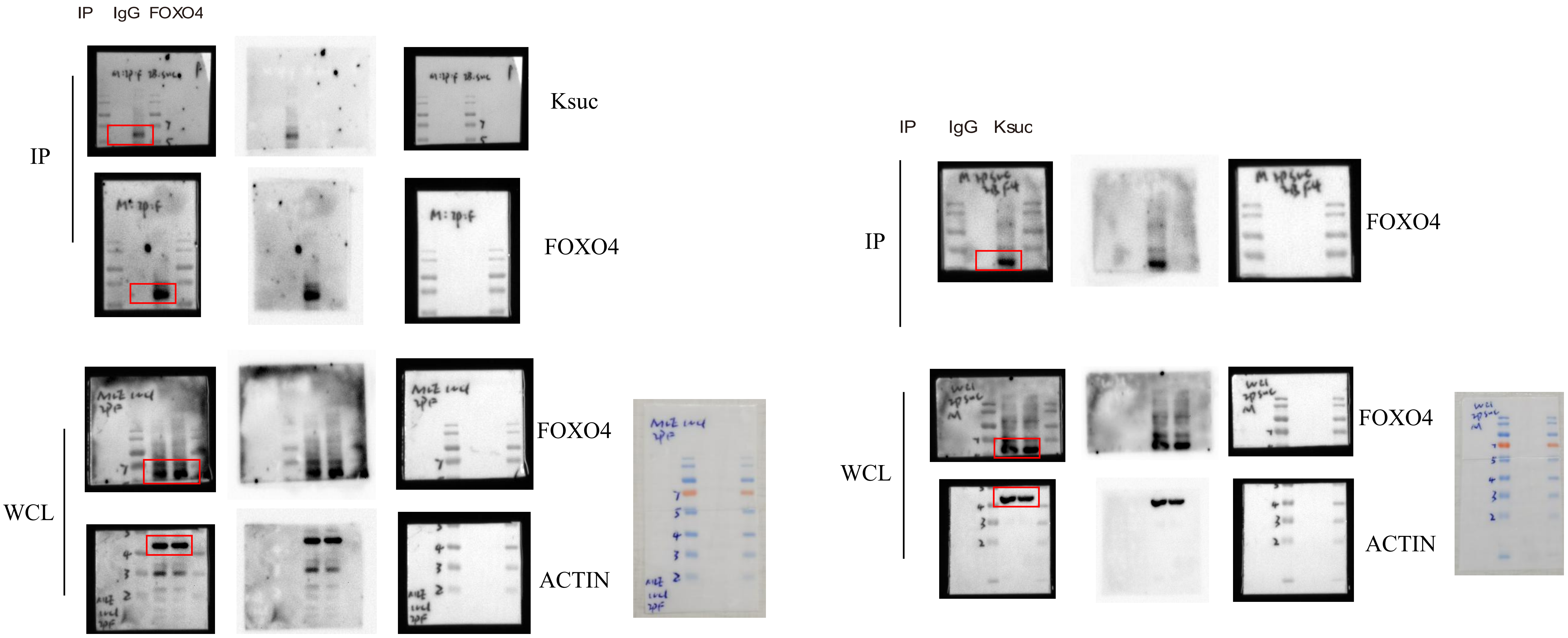
IP
IgG
FOXO4
IP
Ksuc
FOXO4
FOXO4
WCL
ACTIN
IP
IgG
Ksuc
IP
FOXO4
WCL
FOXO4
ACTIN

Fig. 3J

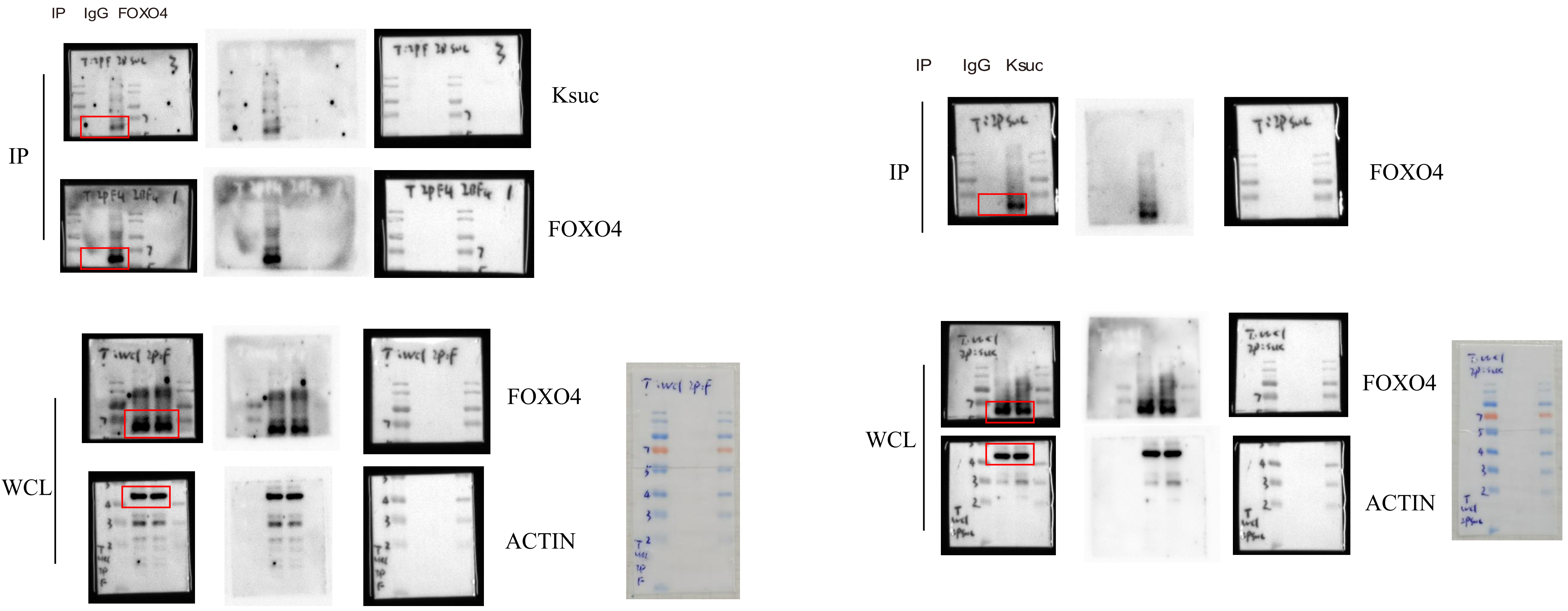
IP IgG FOXO4
IP
Ksuc
FOXO4
WCL
FOXO4
ACTIN
IP IgG Ksuc
IP
FOXO4
WCL
FOXO4
ACTIN

HEK293T

Fig. 3K

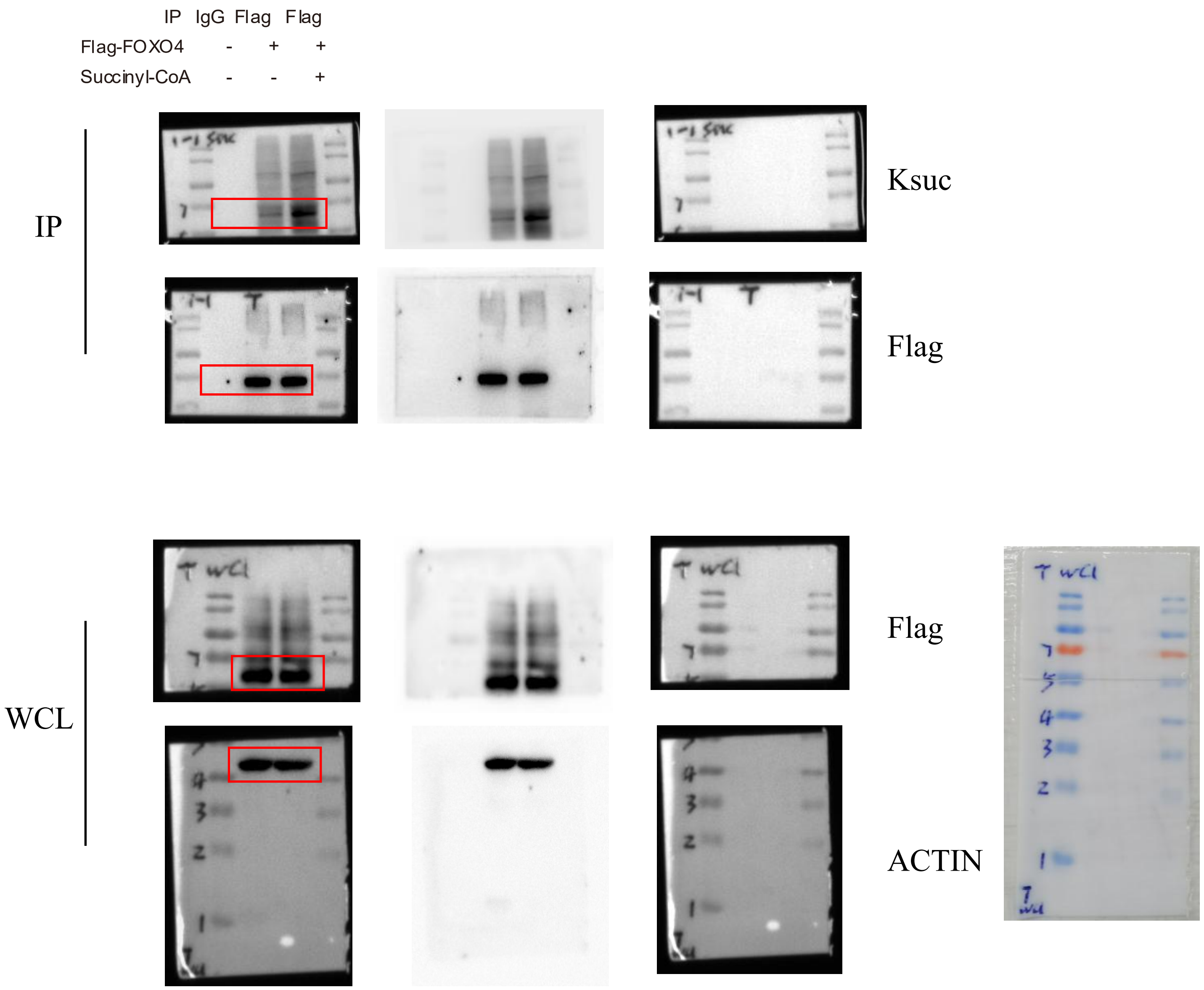

Fig. 4B

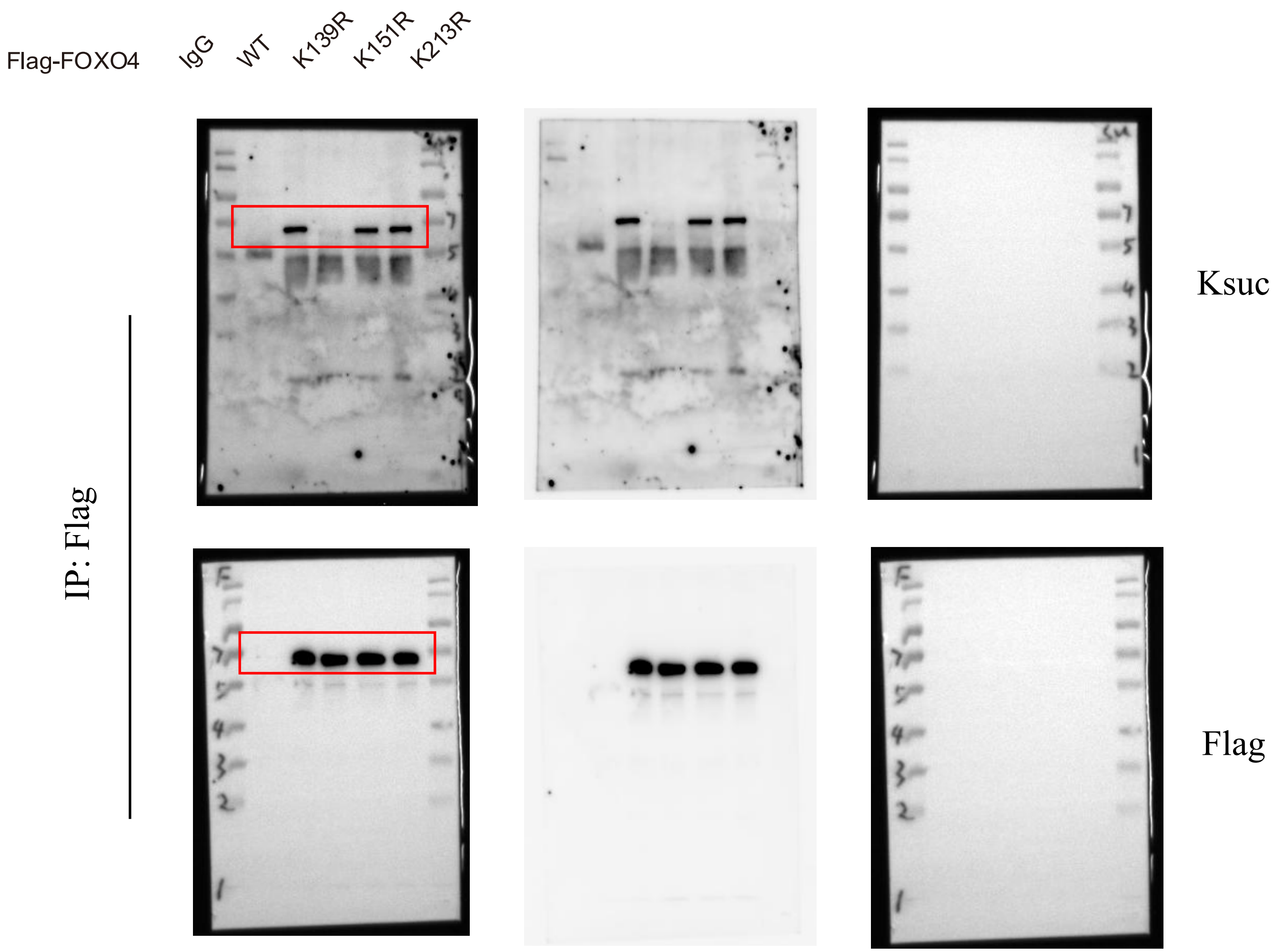

Fig. 4C

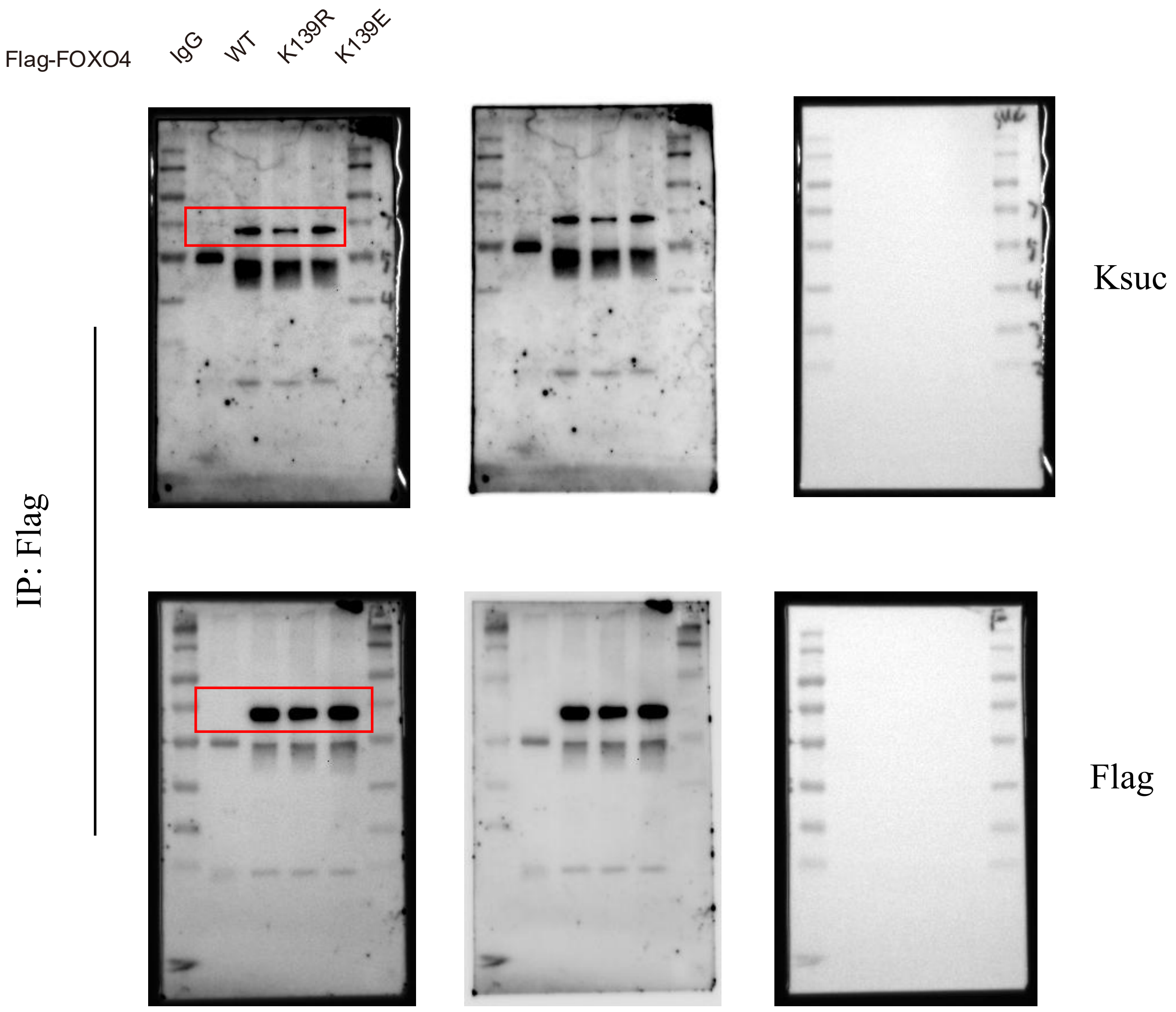

Fig. 4D

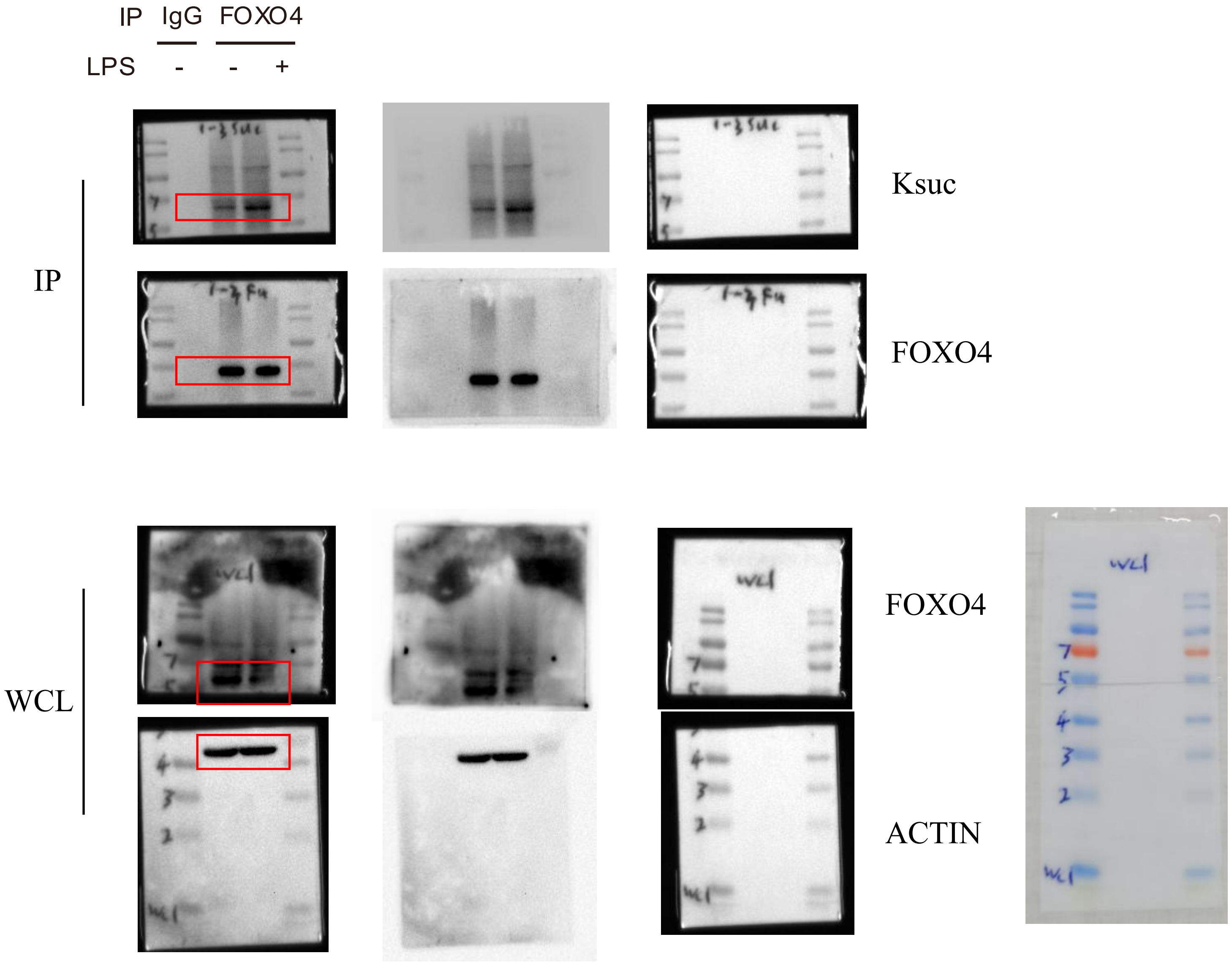

Fig. 4E

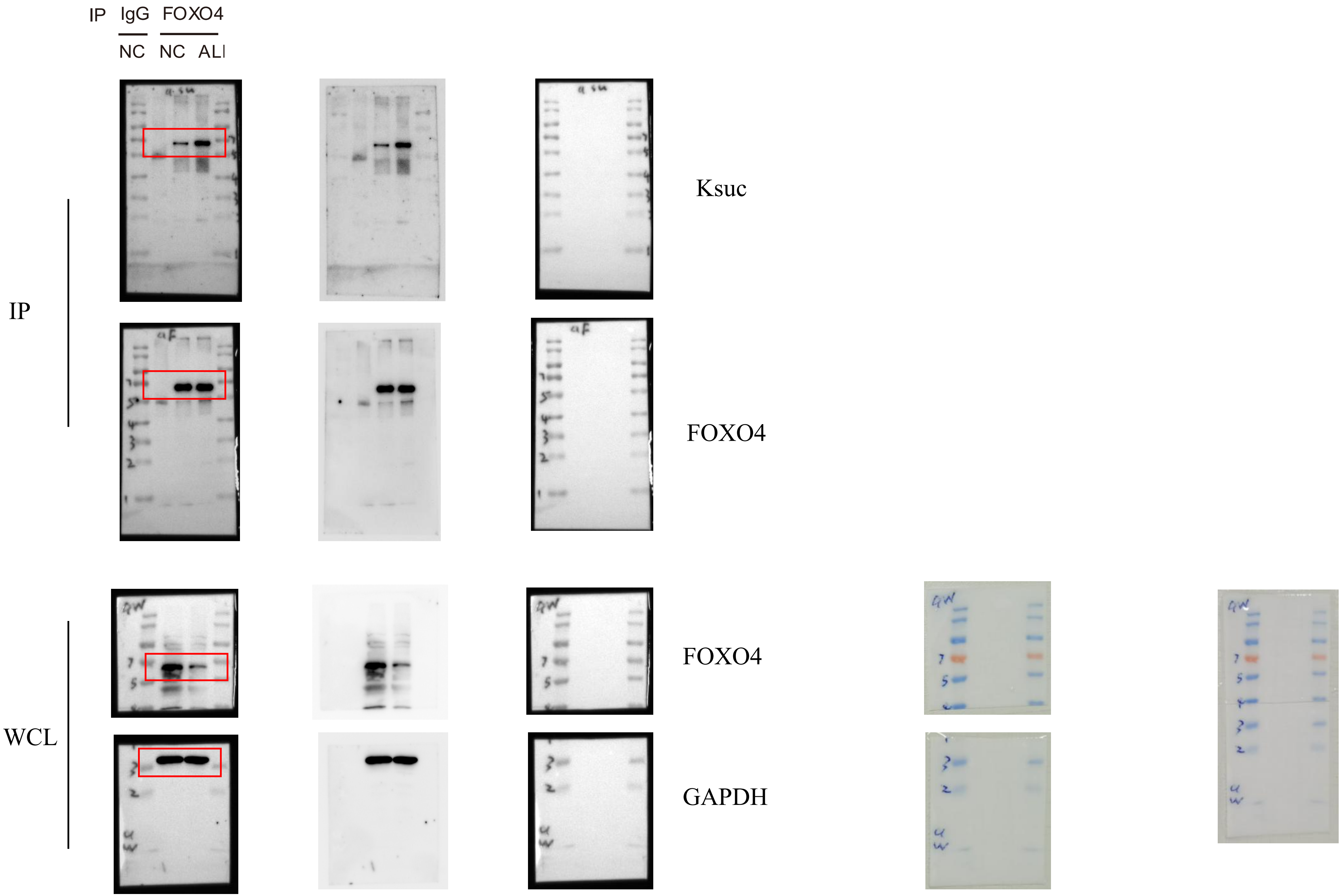

Fig. 4F

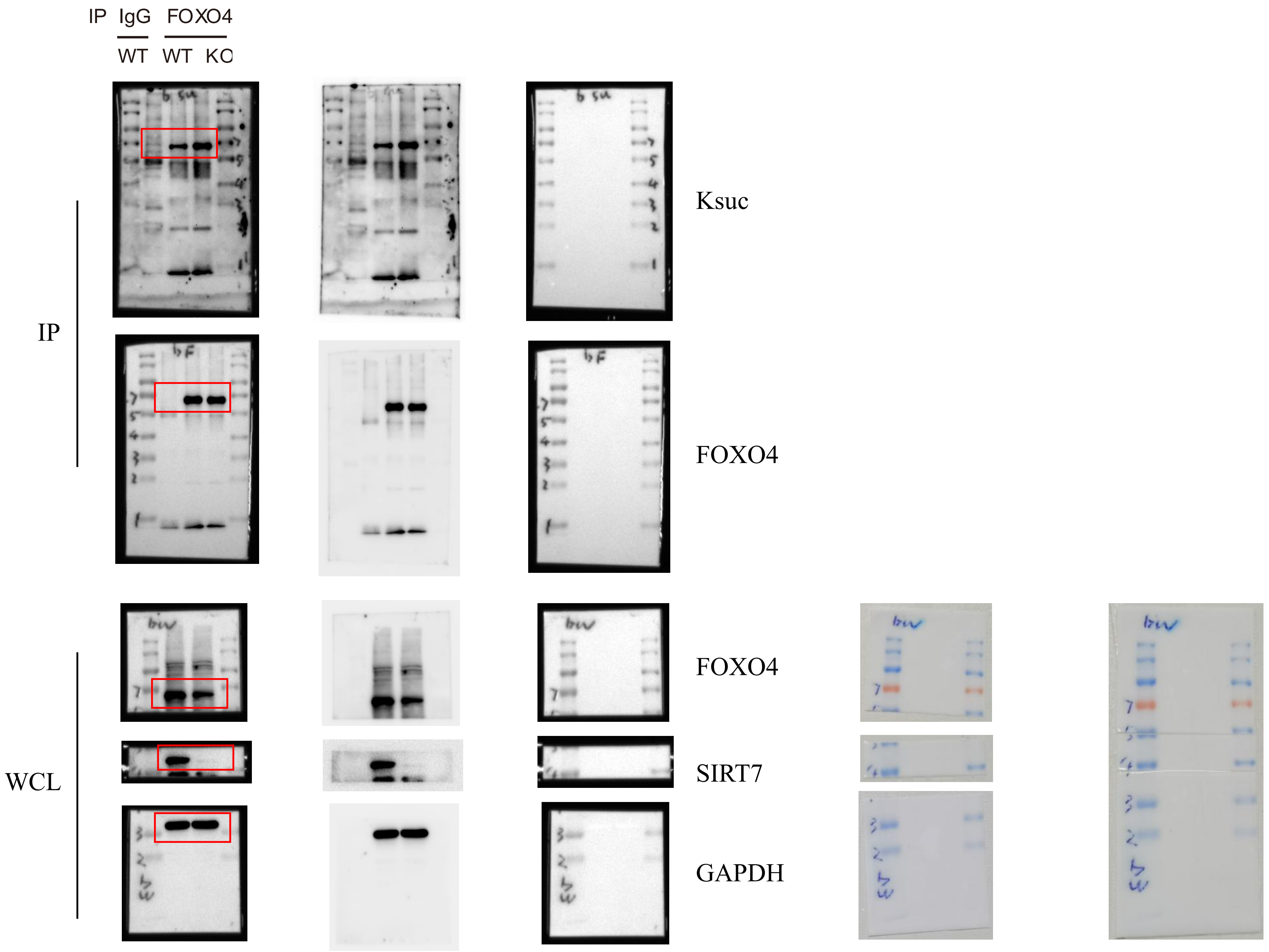

Fig. 4G

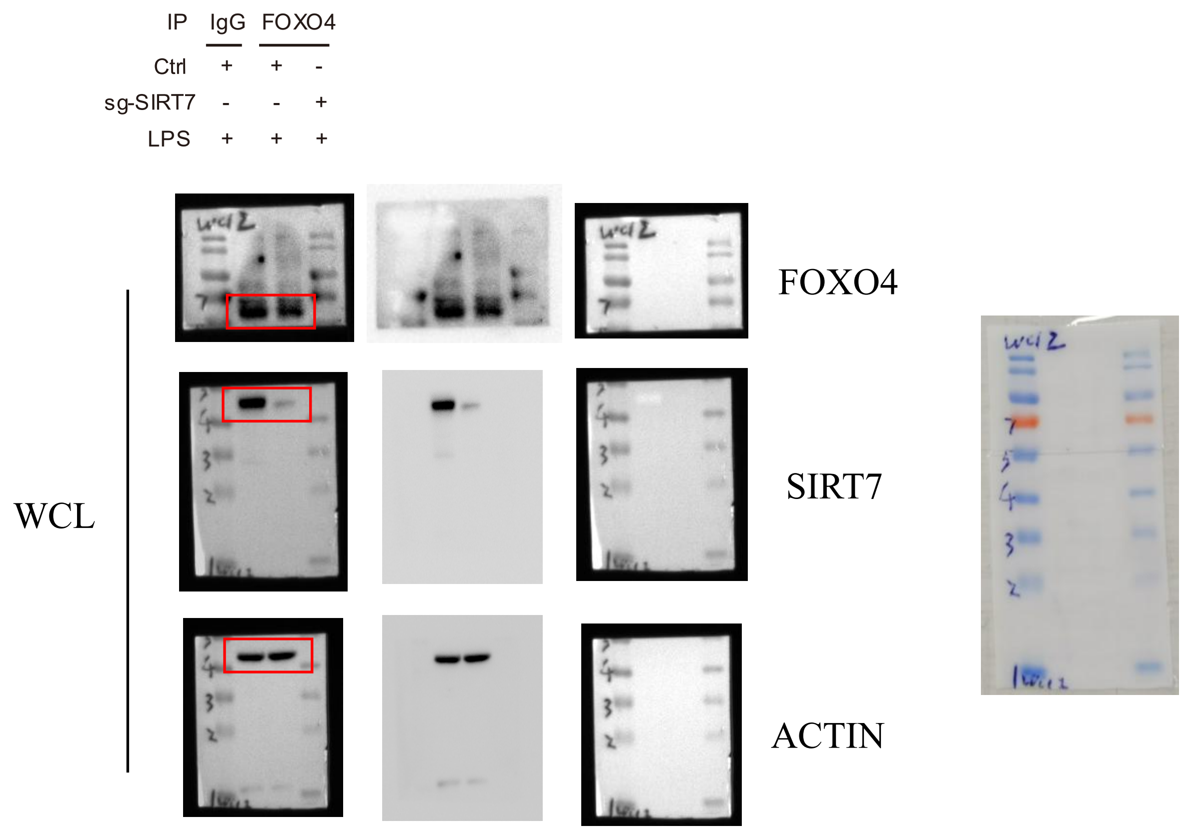
IP IgG FOXO4
Ctrl + + -
sg-SIRT7 - - +
LPS + + +
WCL
FOXO4
SIRT7
ACTIN

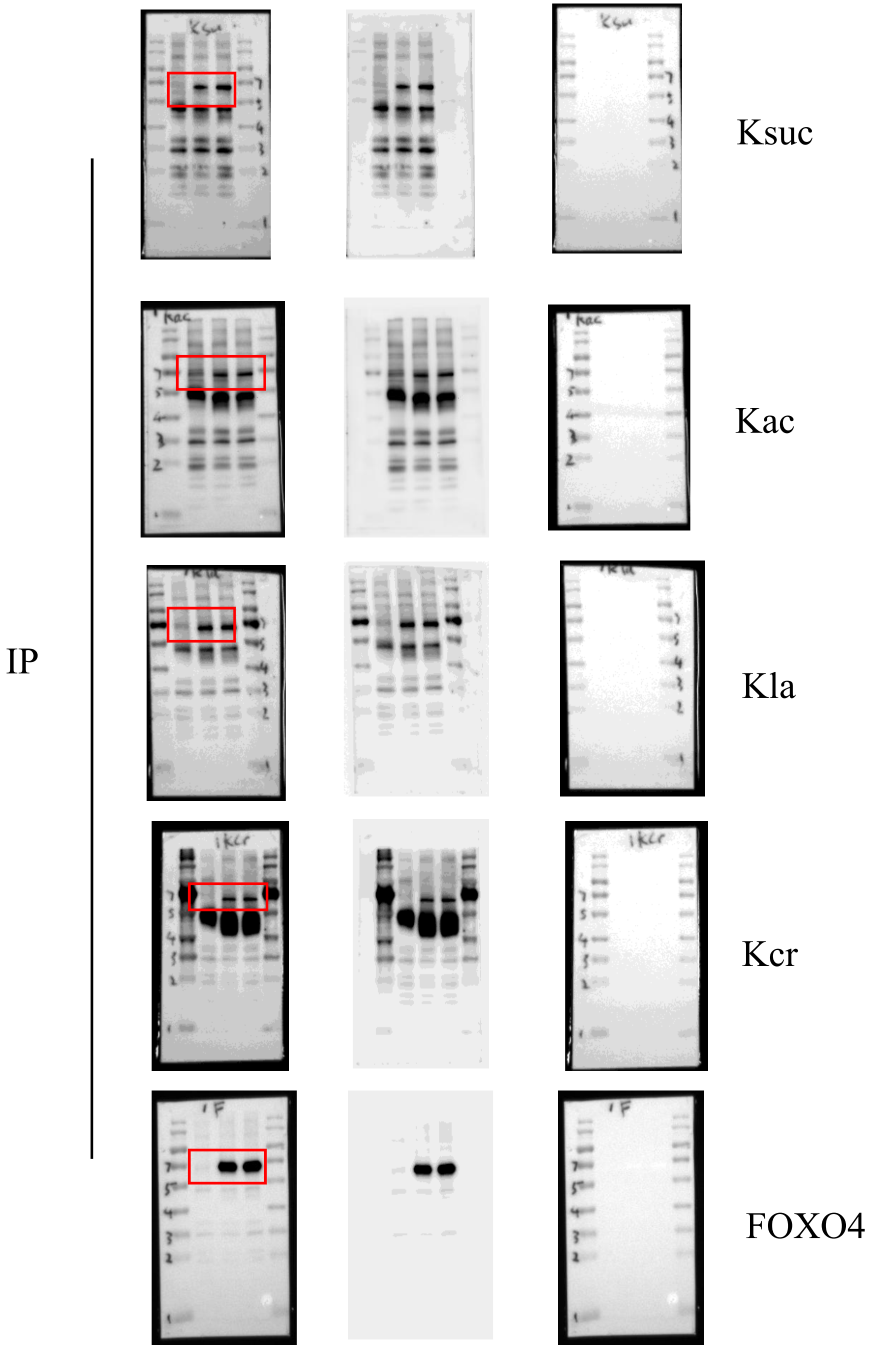
IP
Ksuc
Kac
Kla
Kcr
FOXO4

Fig. 4H

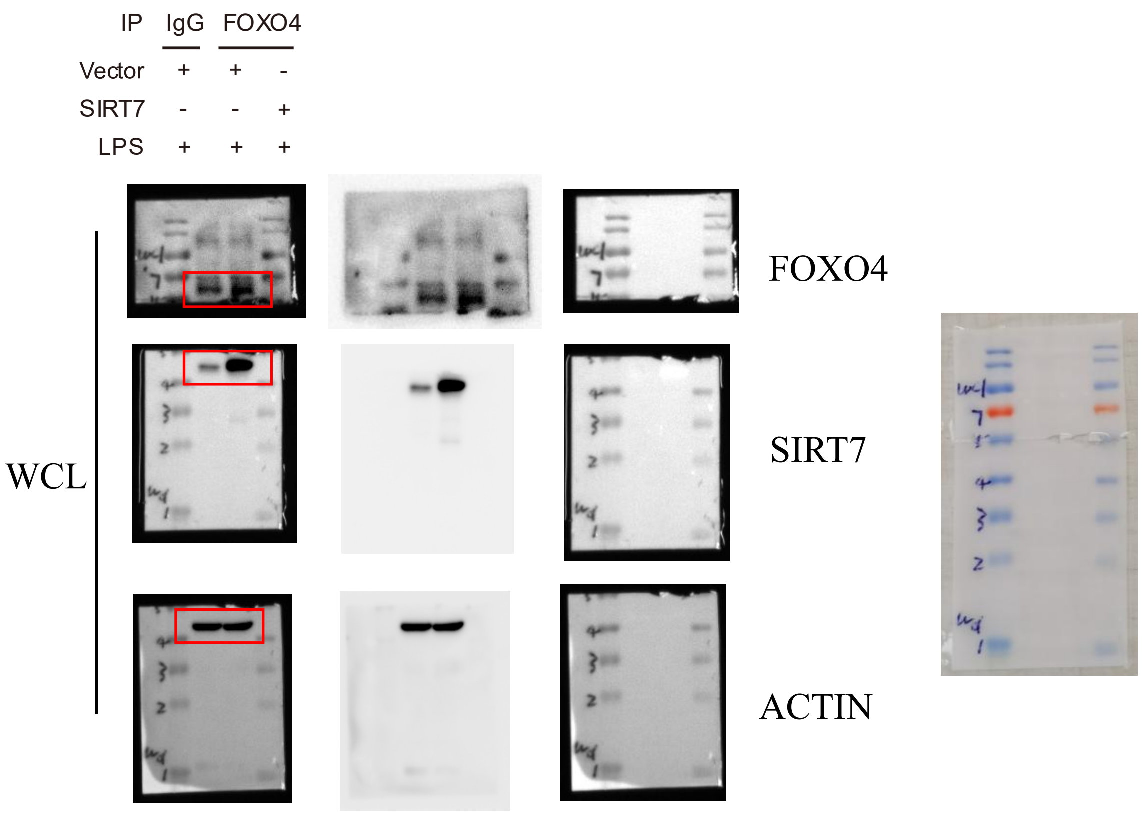
IP IgG FOXO4
Vector + + -
SIRT7 - - +
LPS + + +
WCL
FOXO4
SIRT7
ACTIN

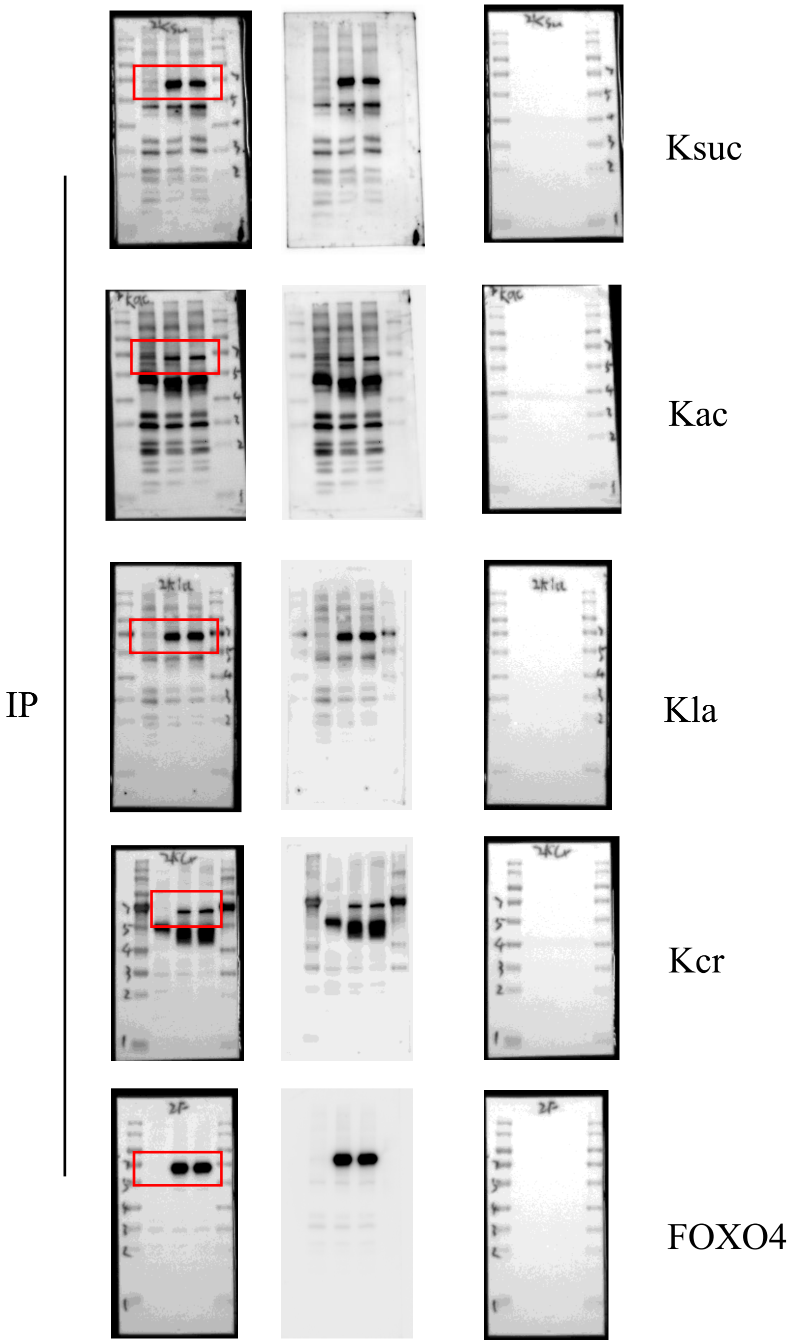
IP
Ksuc
Kac
Kla
Kcr
FOXO4

Fig. 4I

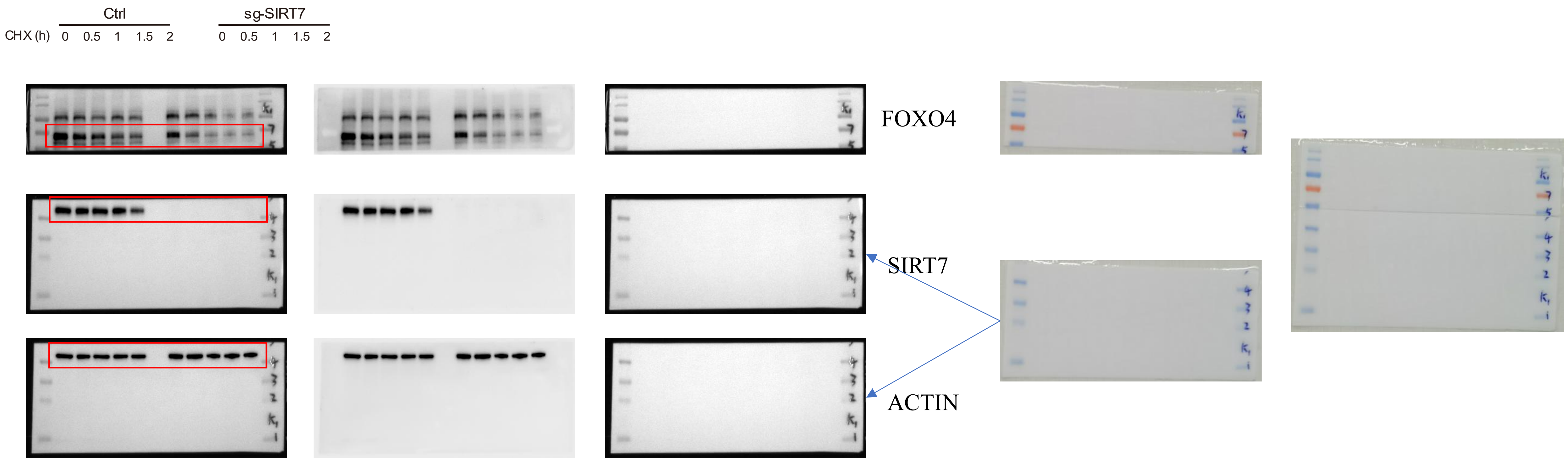

Fig. 4J

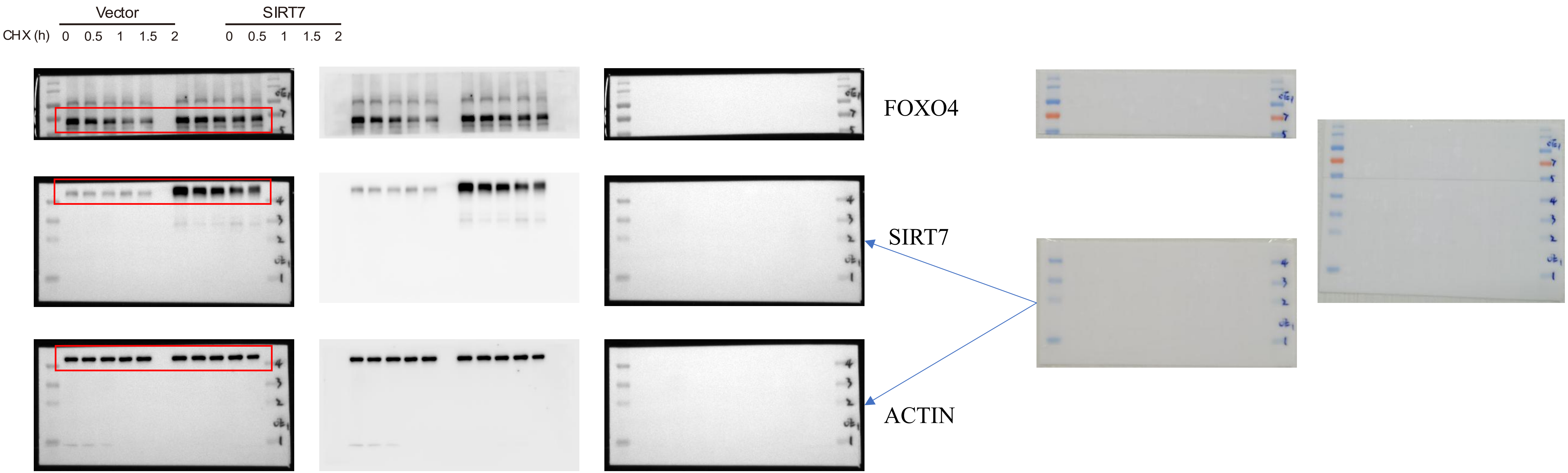

Fig. 4K

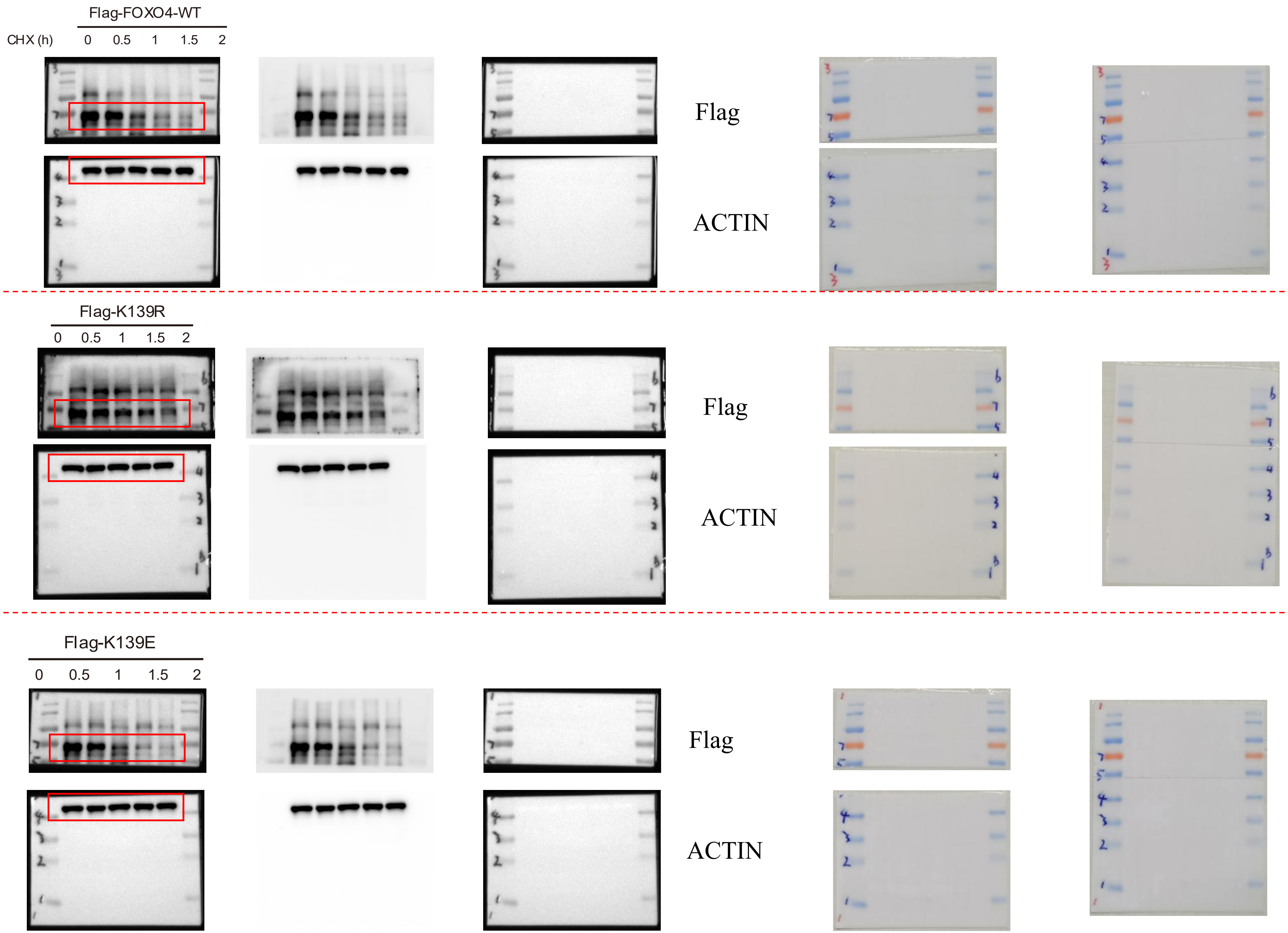

Flag-FOXO4-WT
CHX (h)
0
0.5
1
1.5
2
Flag
ACTIN
Flag-K139R
0
0.5
1
1.5
2
Flag
ACTIN
Flag-K139E
0
0.5
1
1.5
2
Flag
ACTIN

Fig. 4L

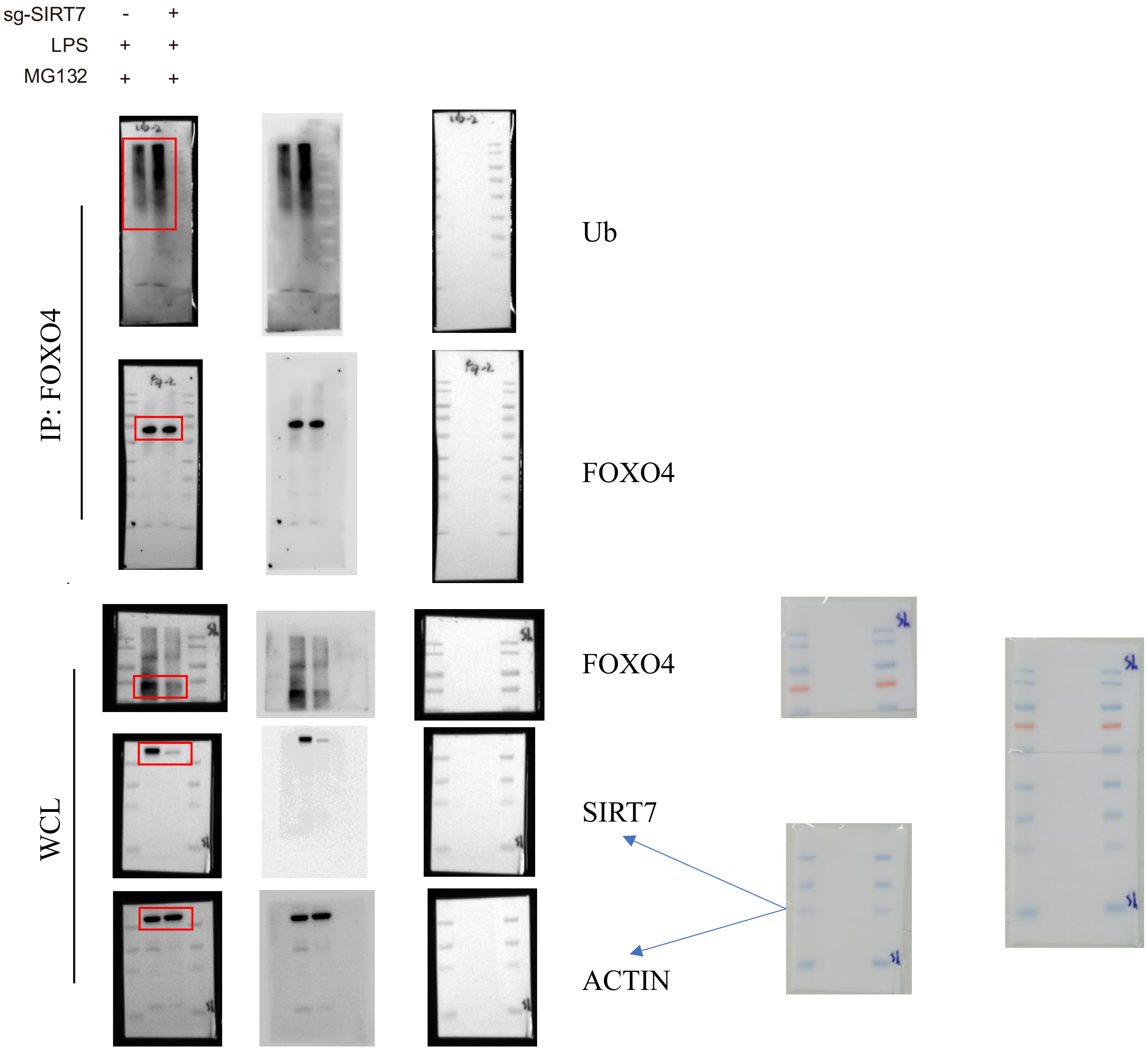

Fig. 4M

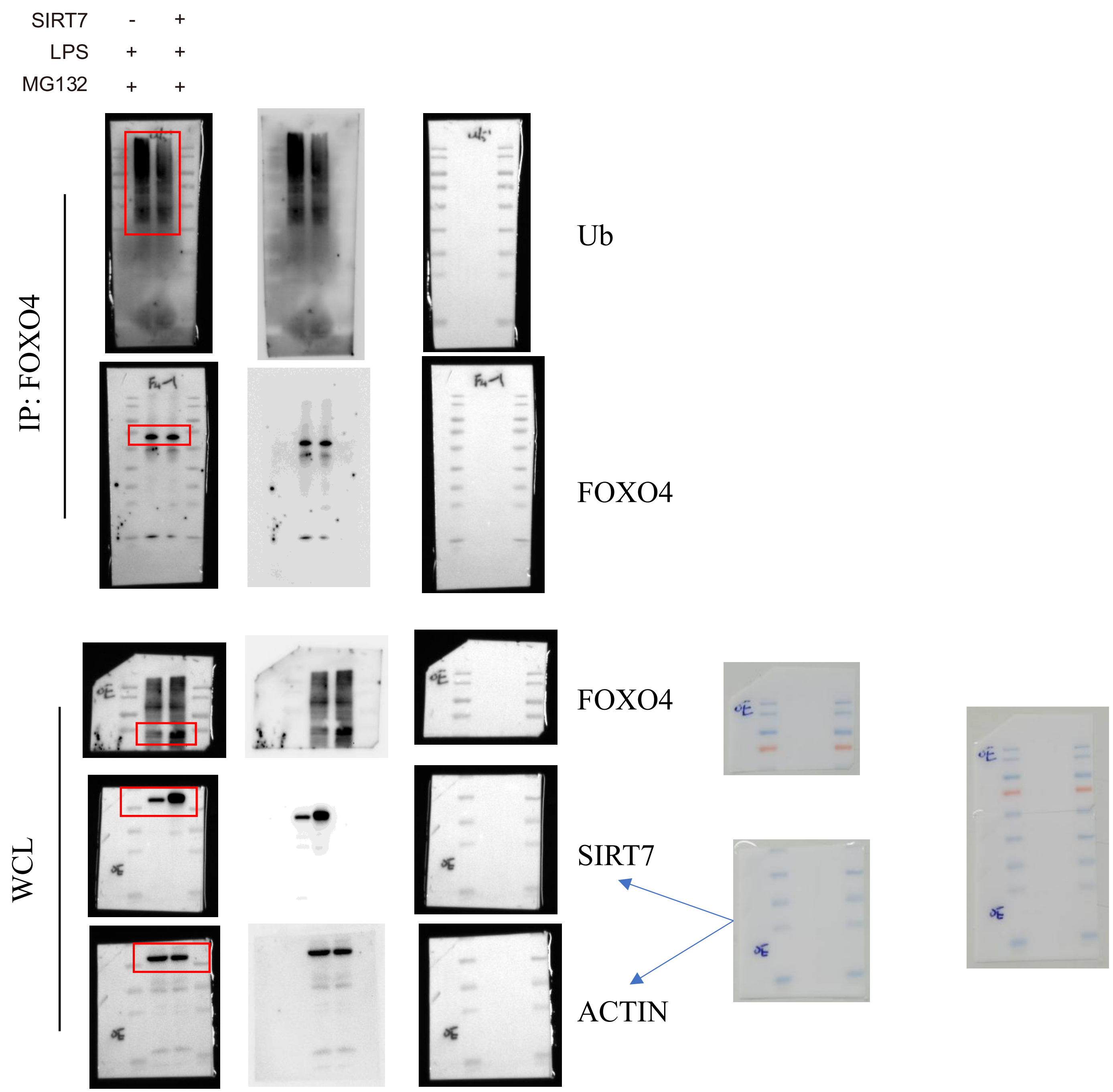

Fig. 4N

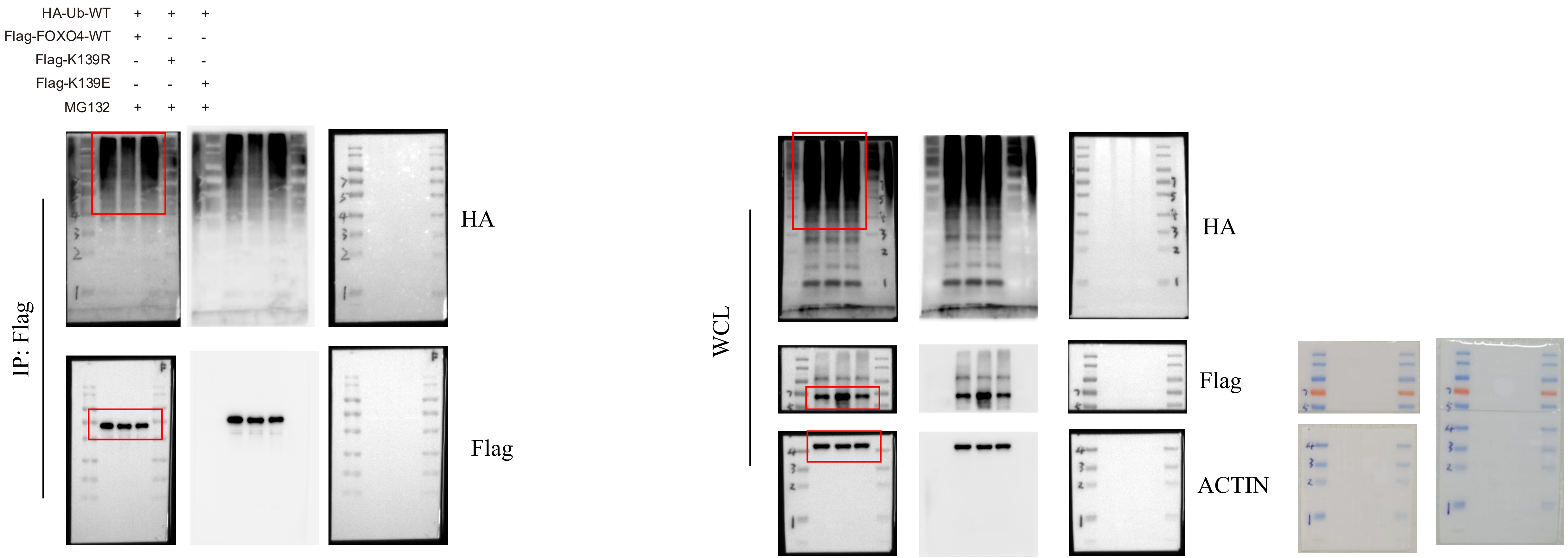
HA-Ub-WT + + +
Flag-FOXO4-WT + - -
Flag-K139R - + -
Flag-K139E - - +
MG132 + + +
IP: Flag
HA
Flag
WCL
HA
Flag
ACTIN

Fig. 4O

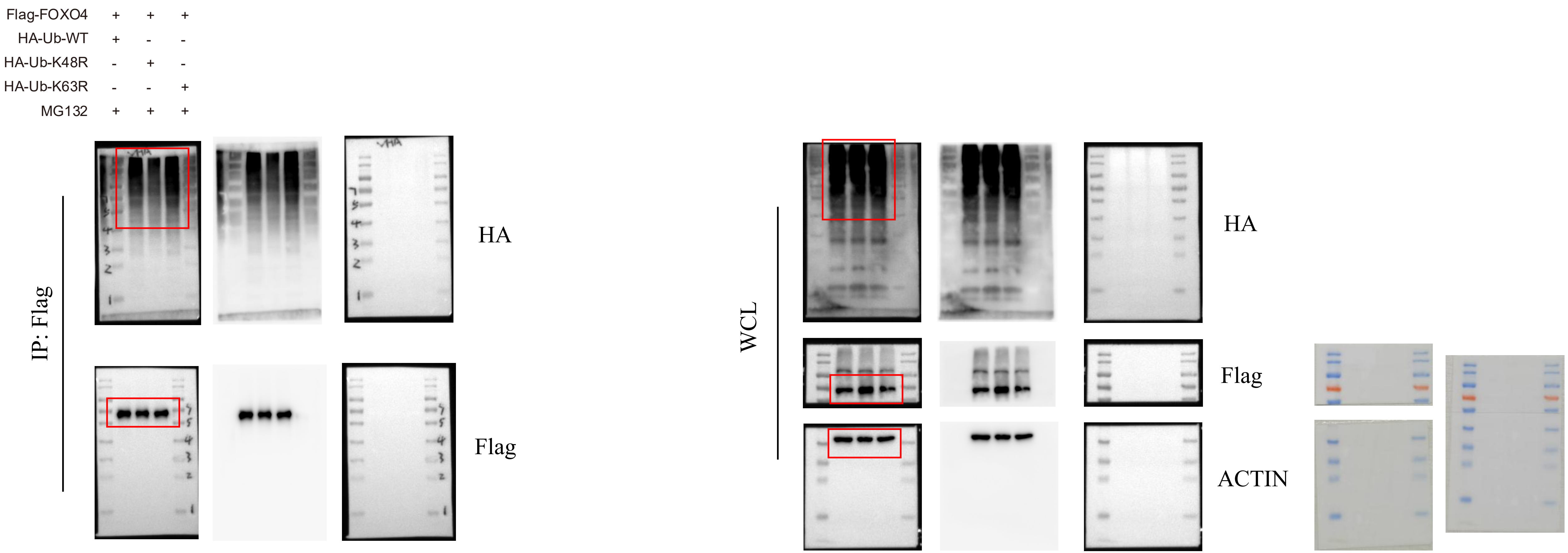

Flag-FOXO4 + + +
HA-Ub-WT + - -
HA-Ub-K48R - + -
HA-Ub-K63R - - +
MG132 + + +
IP: Flag
HA
Flag
WCL
HA
Flag
ACTIN

Fig. 5D

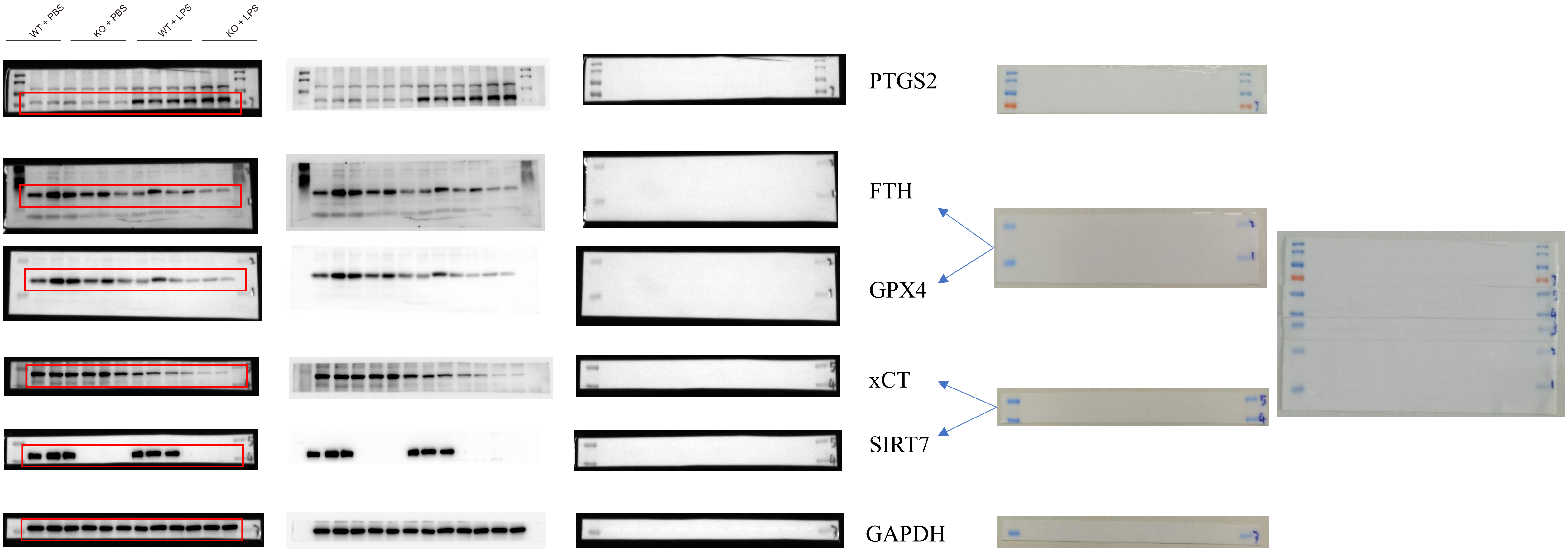

Fig. 6A

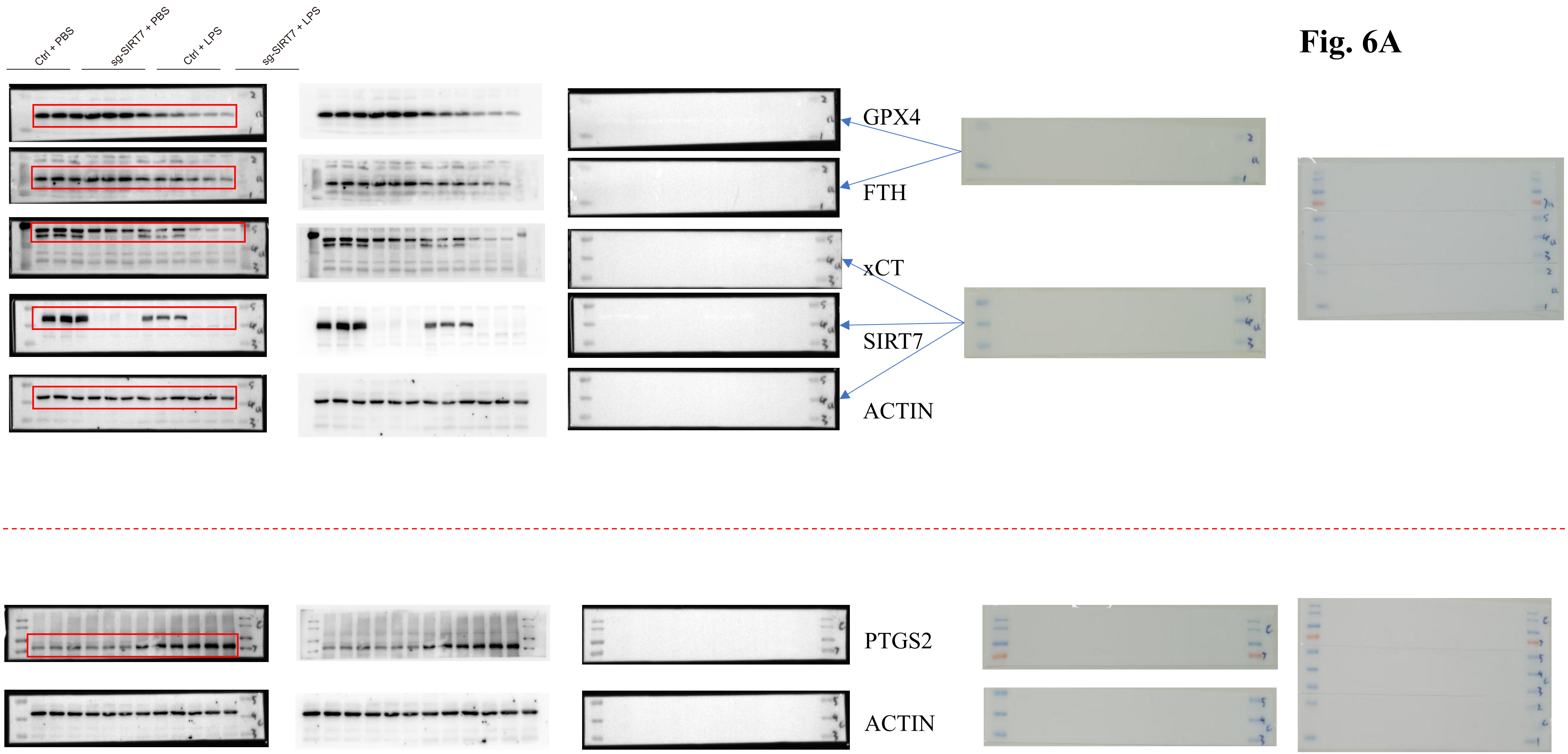
Ctrl + PBS
sg-SIRT7 + PBS
Ctrl + LPS
sg-SIRT7 + LPS
GPX4
FTH
xCT
SIRT7
ACTIN
PTGS2
ACTIN

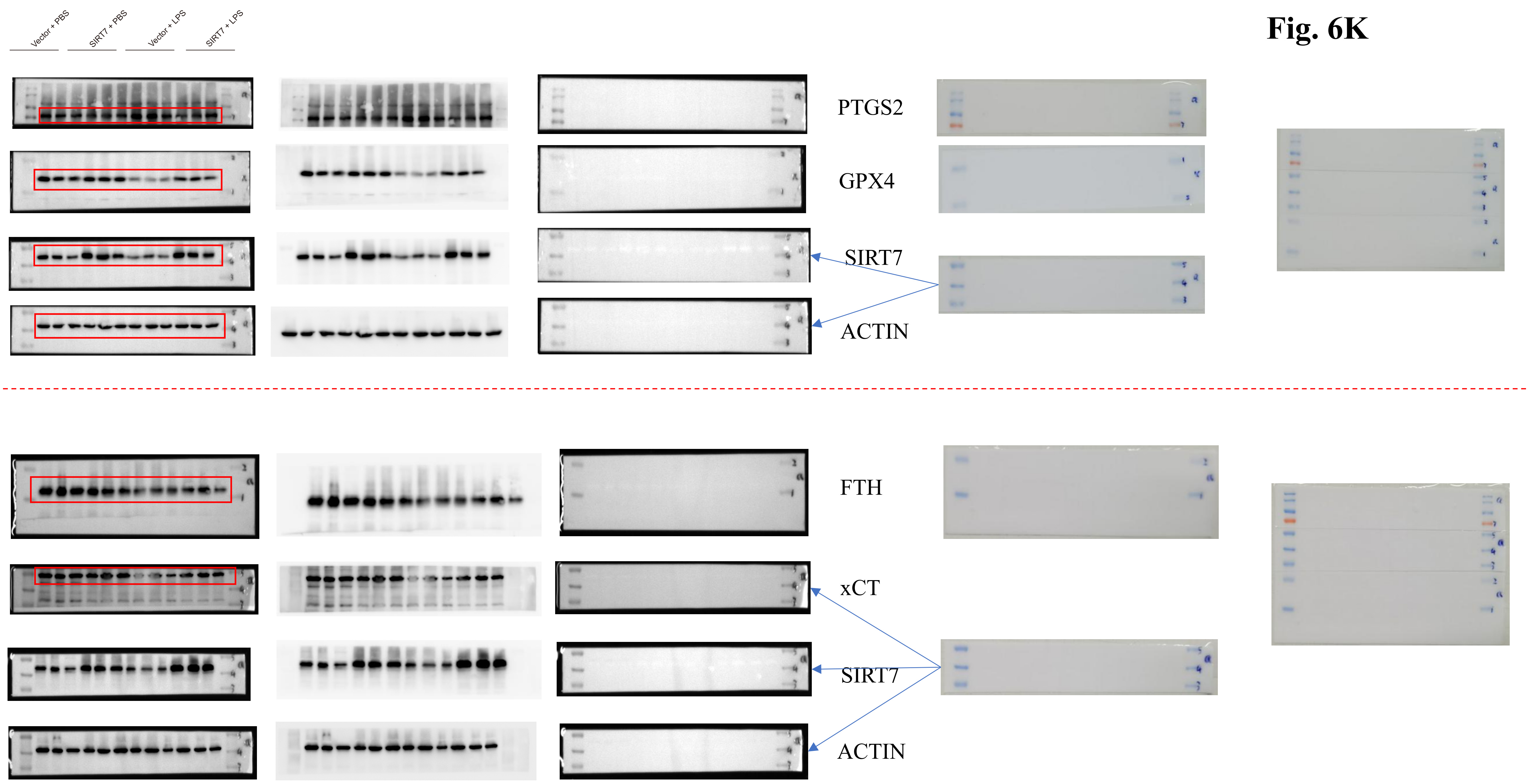
Fig. 6K
Vector + PBS
SIRT7 + PBS
Vector + LPS
SIRT7 + LPS
PTGS2
GPX4
SIRT7
ACTIN
FTH
xCT
SIRT7
ACTIN

Fig. 7N-O

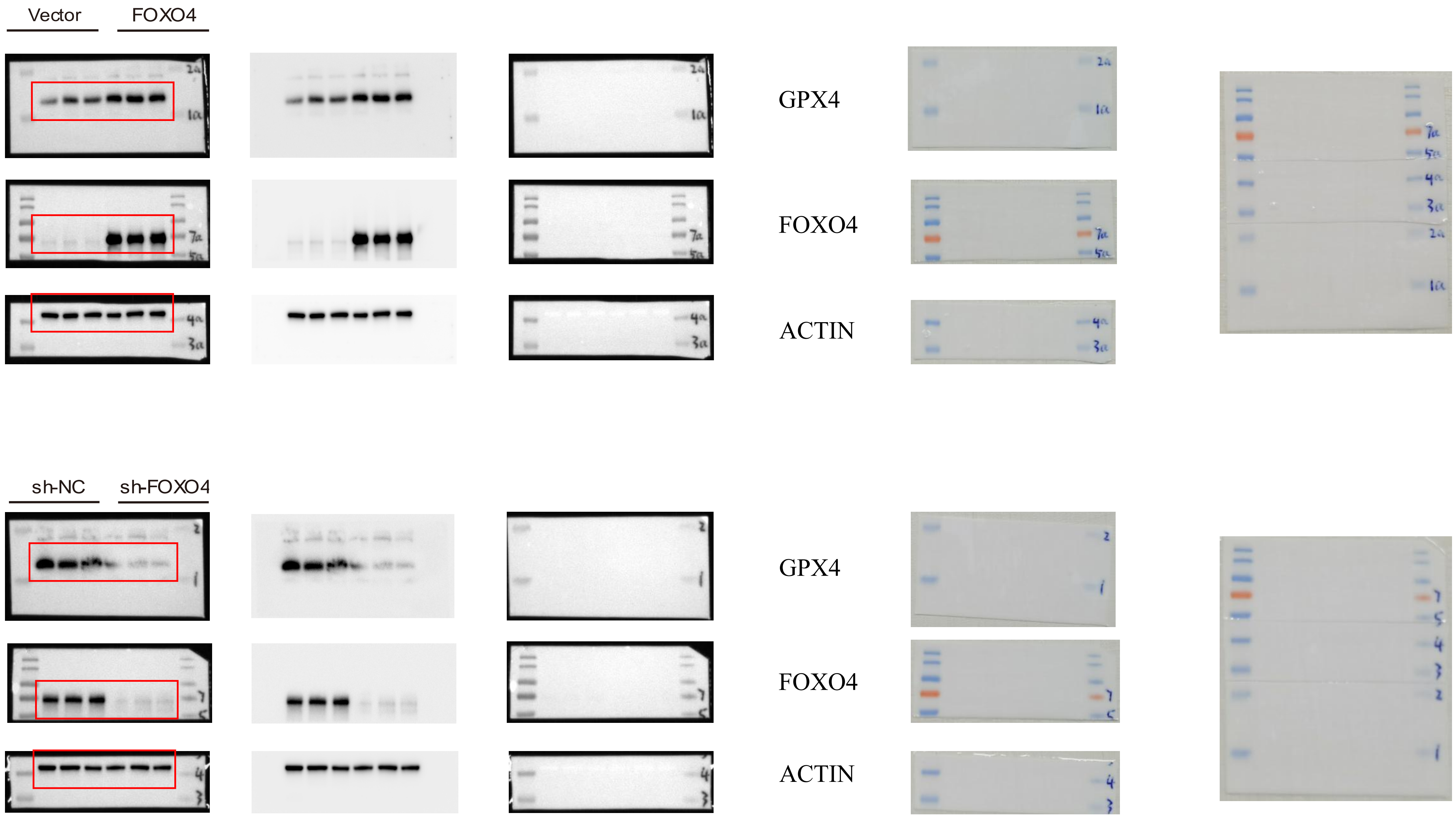
Vector
FOXO4
GPX4
FOXO4
ACTIN
sh-NC
sh-FOXO4
GPX4
FOXO4
ACTIN

Fig. 7P

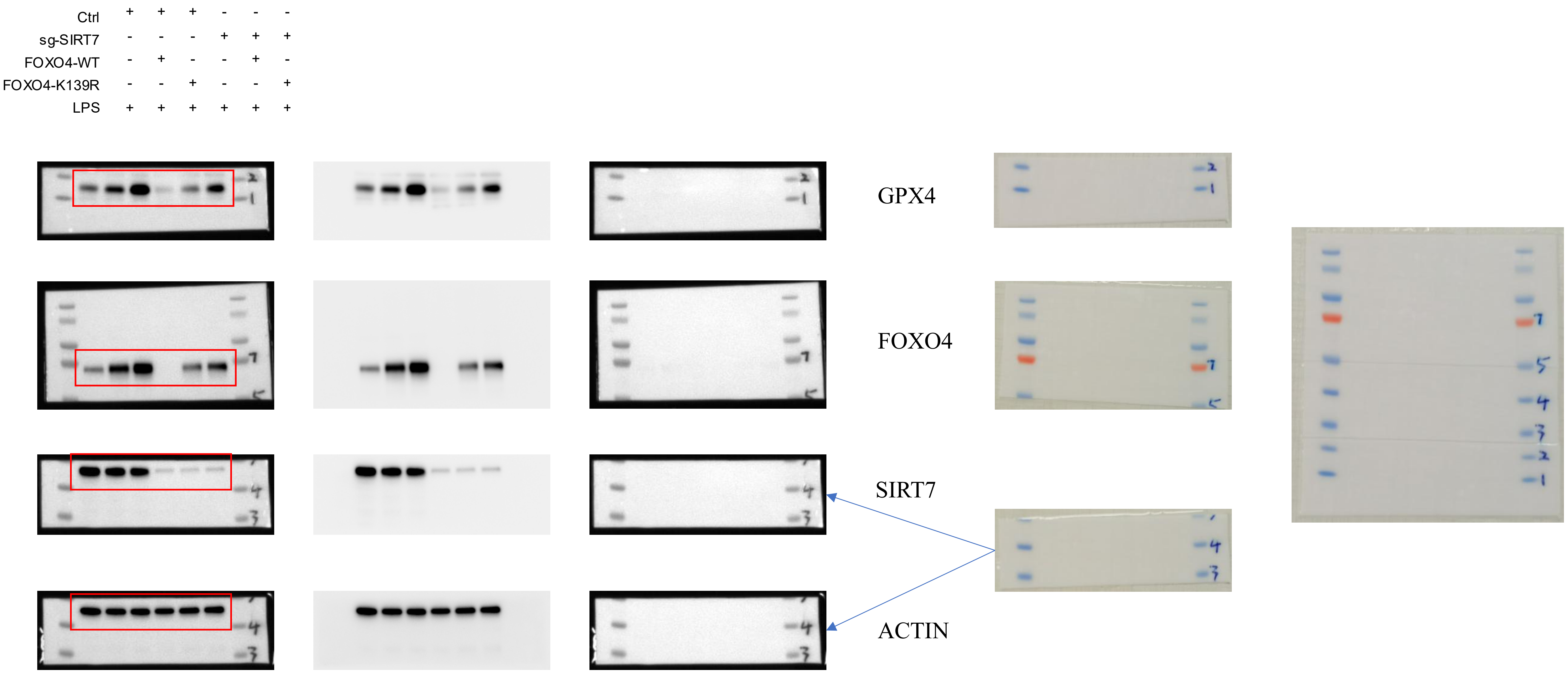

Ctrl + + + - - -
sg-SIRT7 - - - + + +
FOXO4-WT - + - - + -
FOXO4-K139R - - + - - +
LPS + + + + + +
GPX4
FOXO4
SIRT7
ACTIN

Fig. 8J

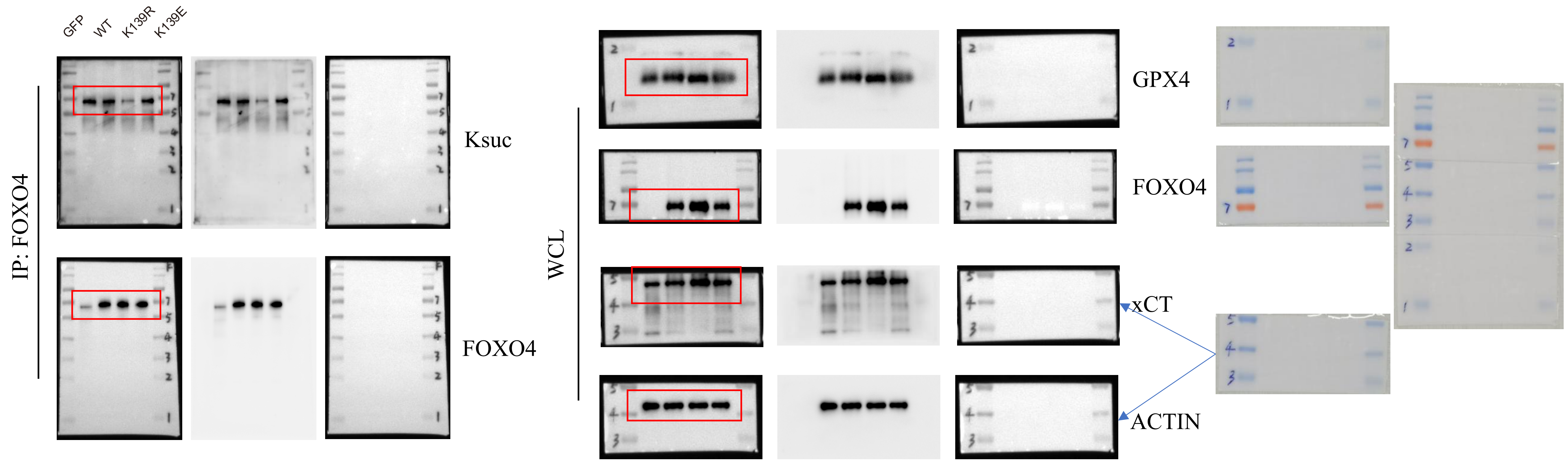

GFP
WT
K139R
K139E
IP: FOXO4
Ksuc
FOXO4
WCL
GPX4
FOXO4
xCT
ACTIN

Fig. 9L

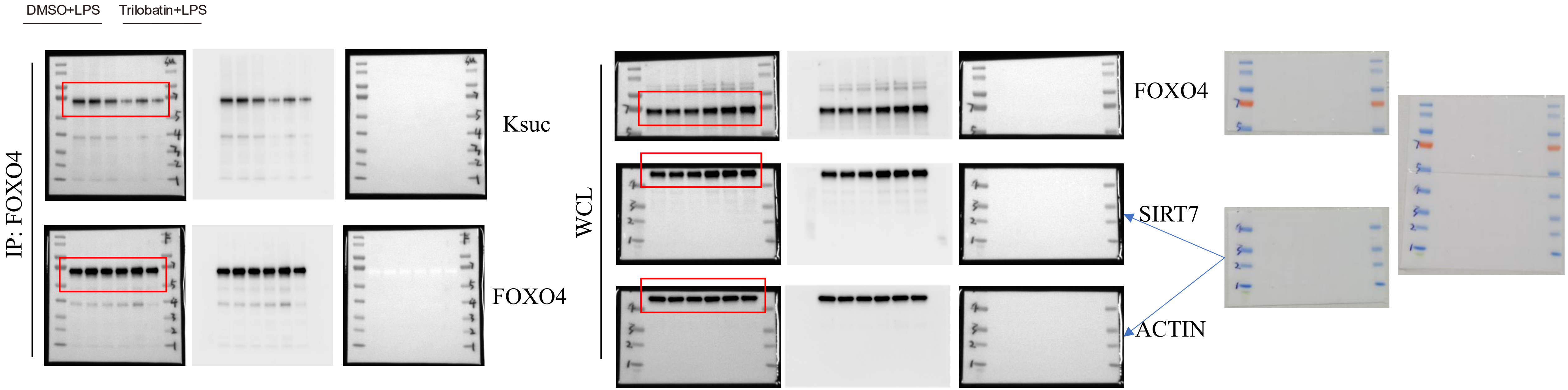

DMSO+LPS
Trilobatin+LPS
IP: FOXO4
Ksuc
FOXO4
WCL
FOXO4
SIRT7
ACTIN

Fig. S2A

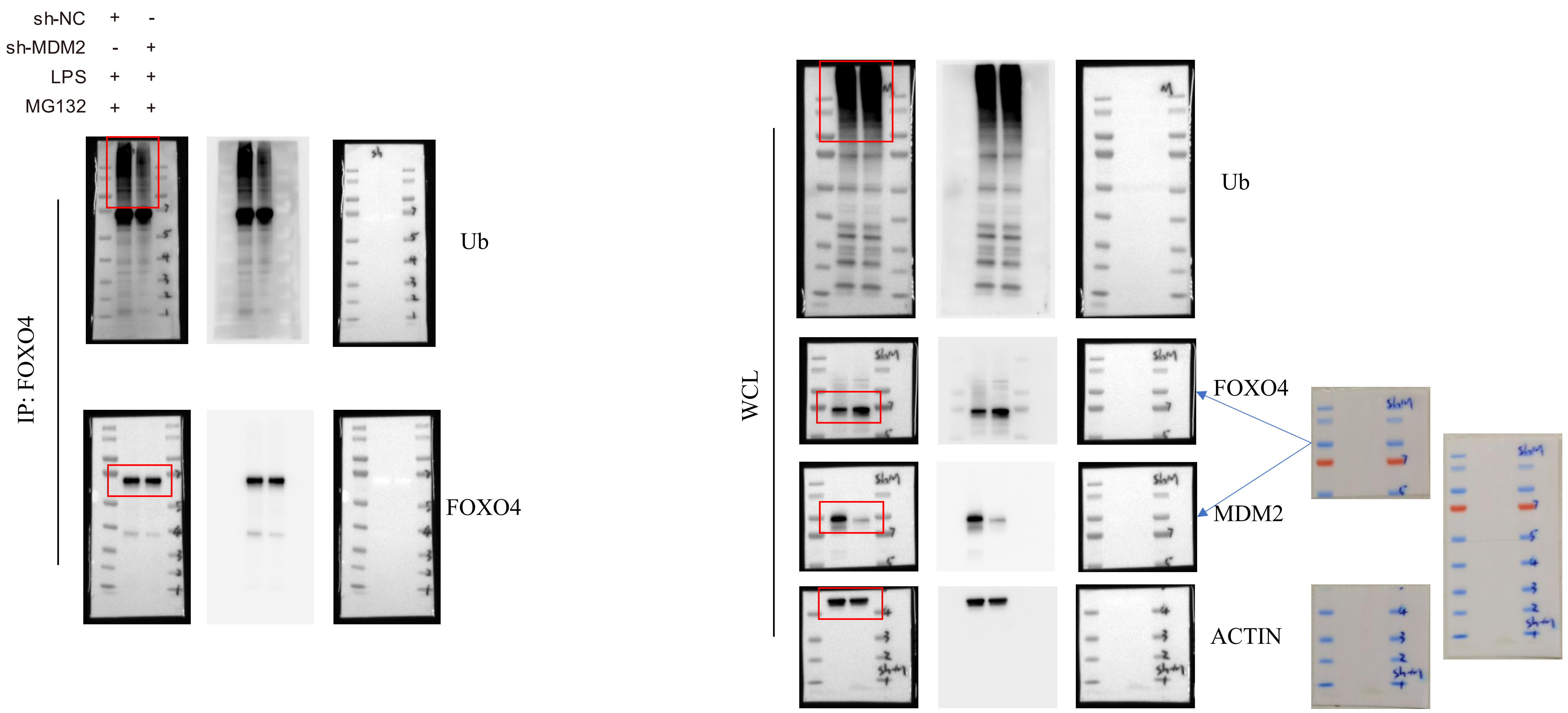
sh-NC + -
sh-MDM2 - +
LPS + +
MG132 + +
IP: FOXO4
Ub
FOXO4
WCL
Ub
FOXO4
MDM2
ACTIN

Fig. S2B

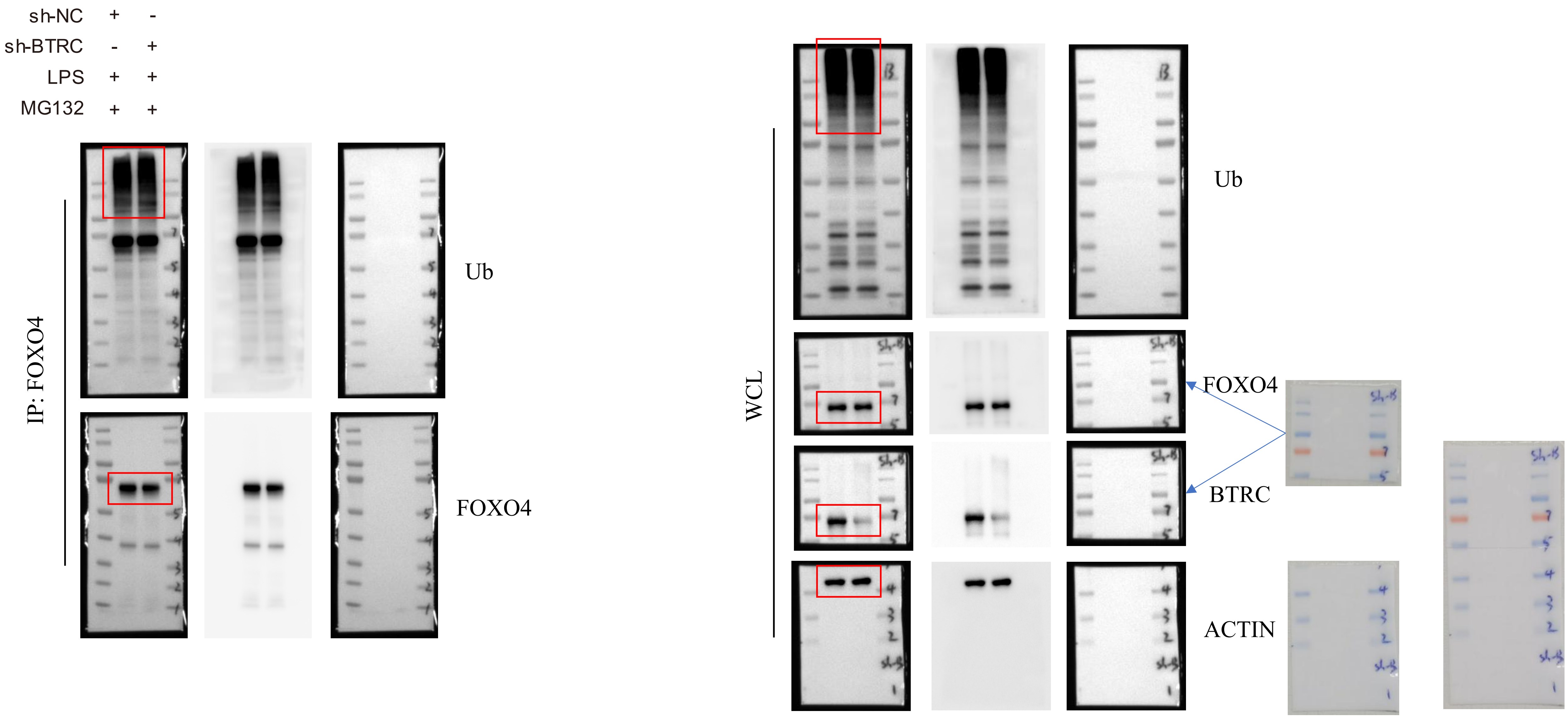

sh-NC + -
sh-BTRC - +
LPS + +
MG132 + +
IP: FOXO4
Ub
FOXO4
WCL
Ub
FOXO4
BTRC
ACTIN

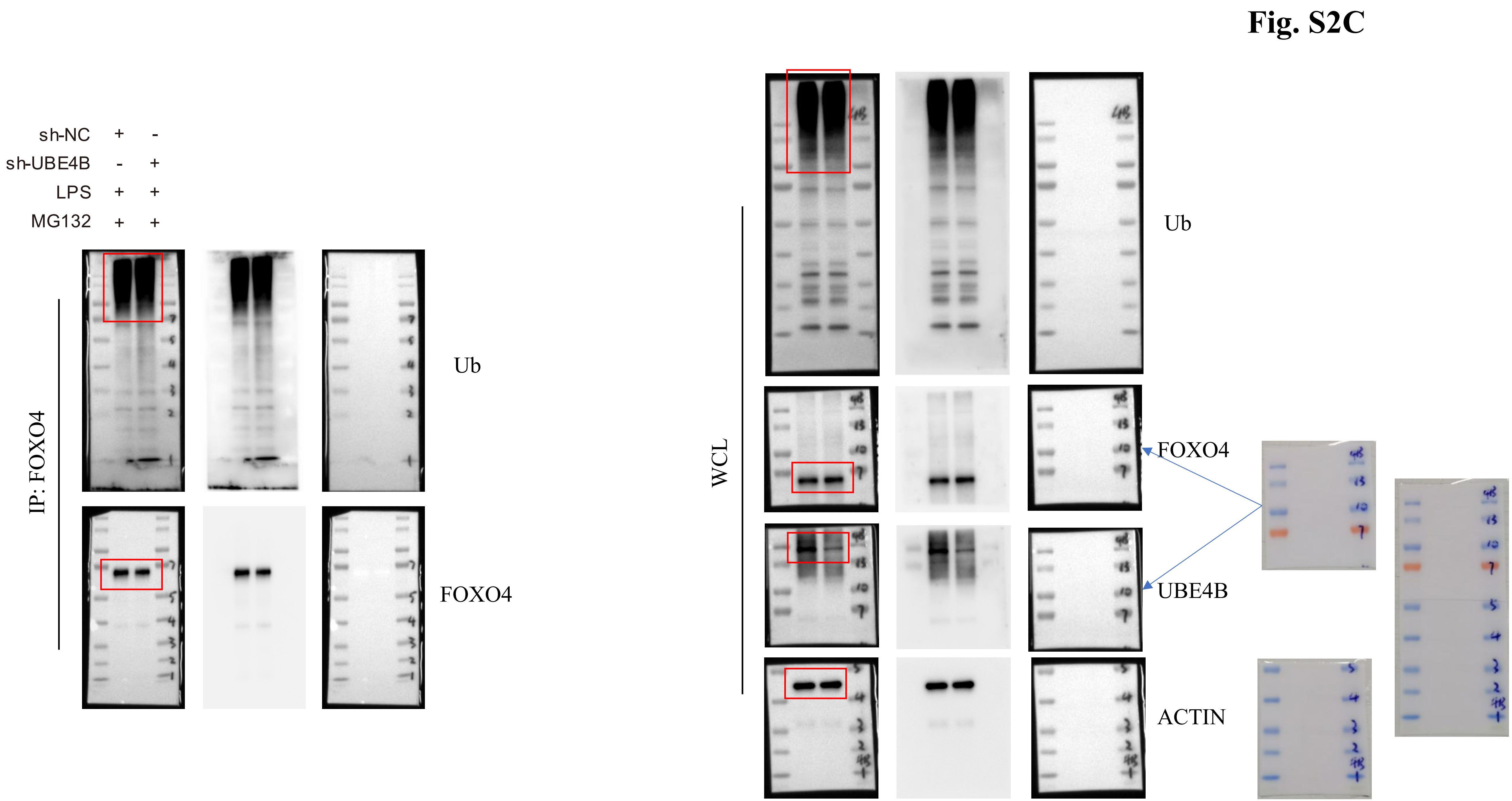
Fig. S2C
sh-NC + -
sh-UBE4B - +
LPS + +
MG132 + +
IP: FOXO4
Ub
FOXO4
WCL
Ub
FOXO4
UBE4B
ACTIN

Fig. S2D

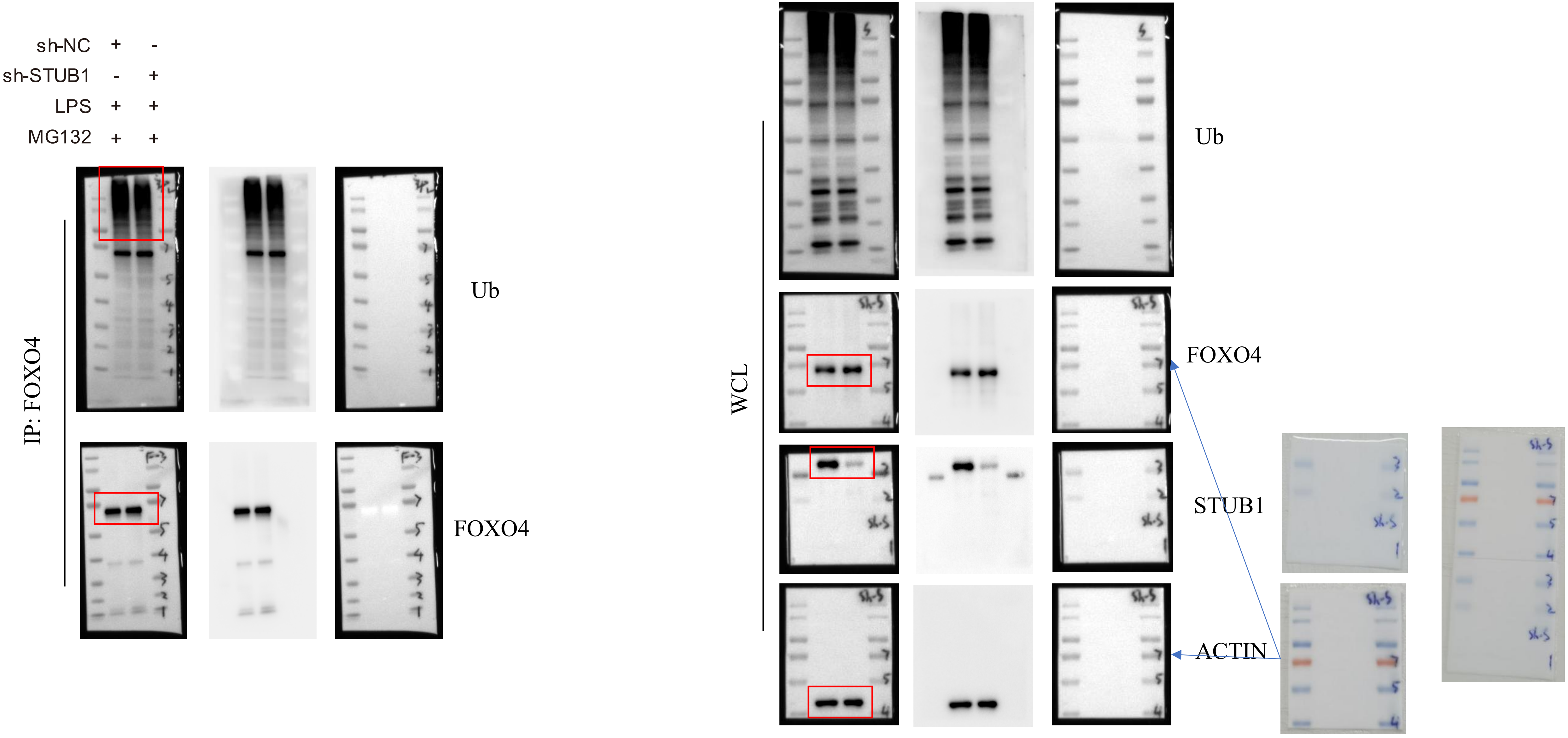
sh-NC + -
sh-STUB1 - +
LPS + +
MG132 + +
IP: FOXO4
Ub
FOXO4
WCL
Ub
FOXO4
STUB1
ACTIN

Fig. S2E

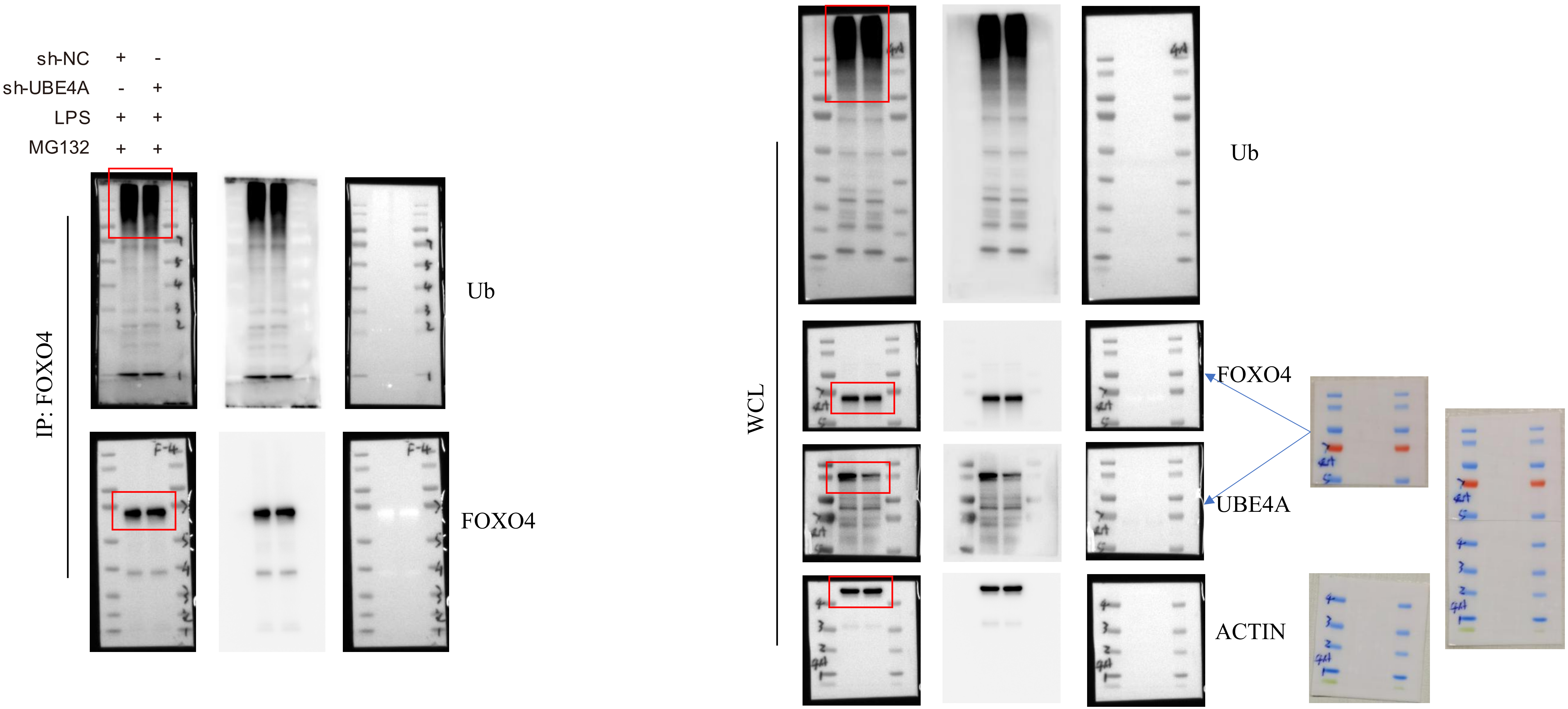
sh-NC + -
sh-UBE4A - +
LPS + +
MG132 + +
IP: FOXO4
Ub
FOXO4
WCL
Ub
FOXO4
UBE4A
ACTIN

Fig. S2F

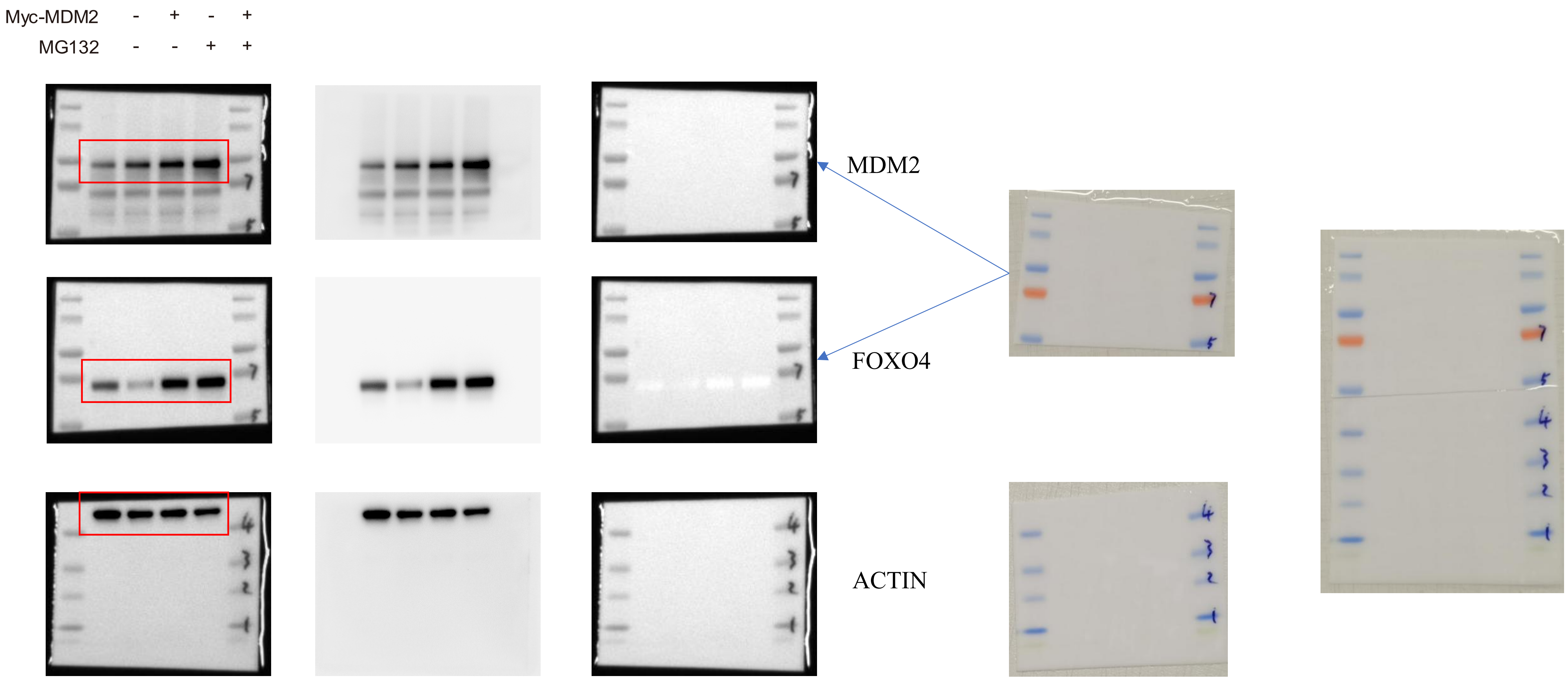

Fig. S2G

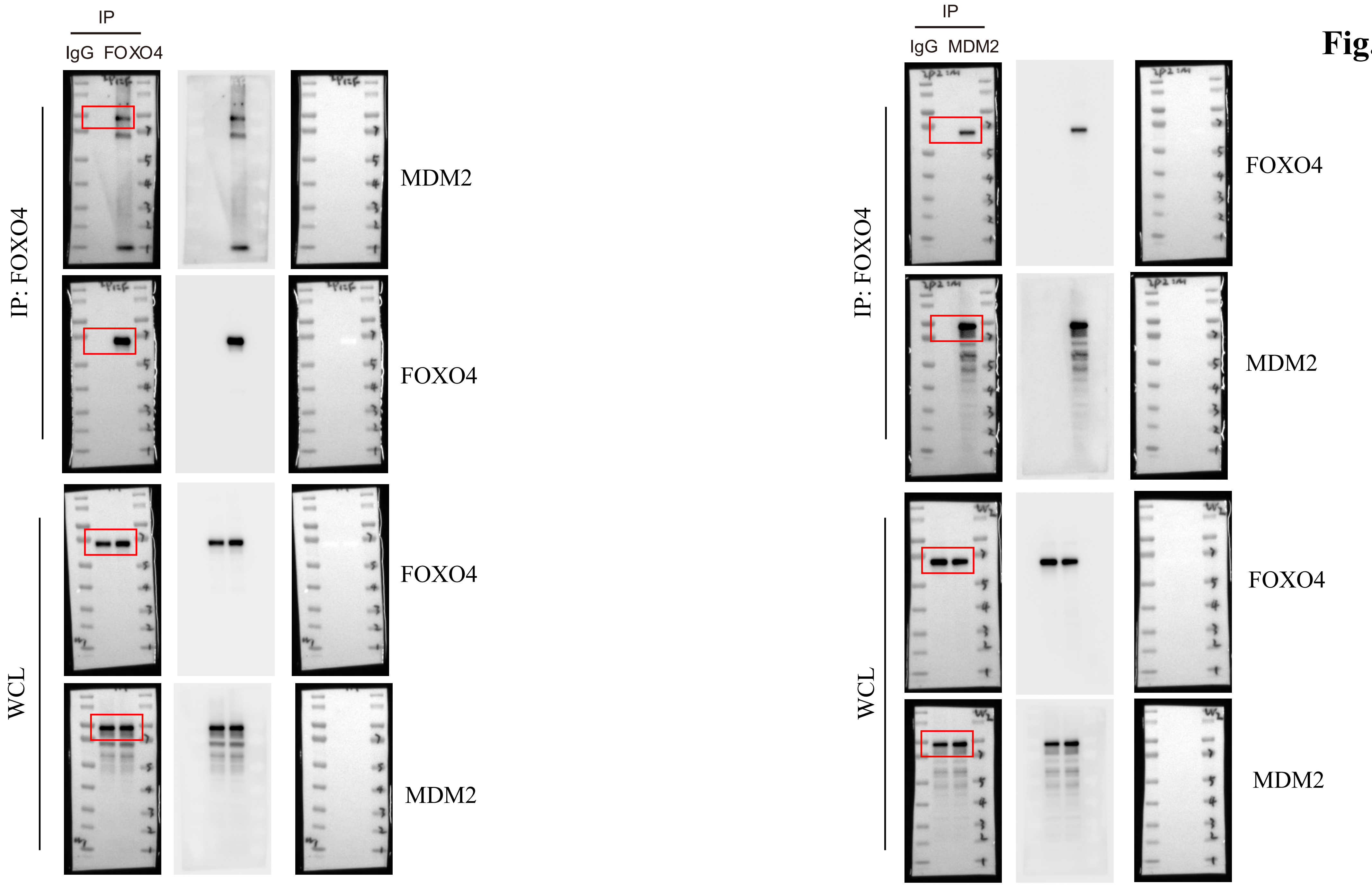
IP
IgG FOXO4
IP: FOXO4
MDM2
FOXO4
WCL
FOXO4
MDM2
IP
IgG MDM2
IP: FOXO4
FOXO4
MDM2
WCL
FOXO4
MDM2

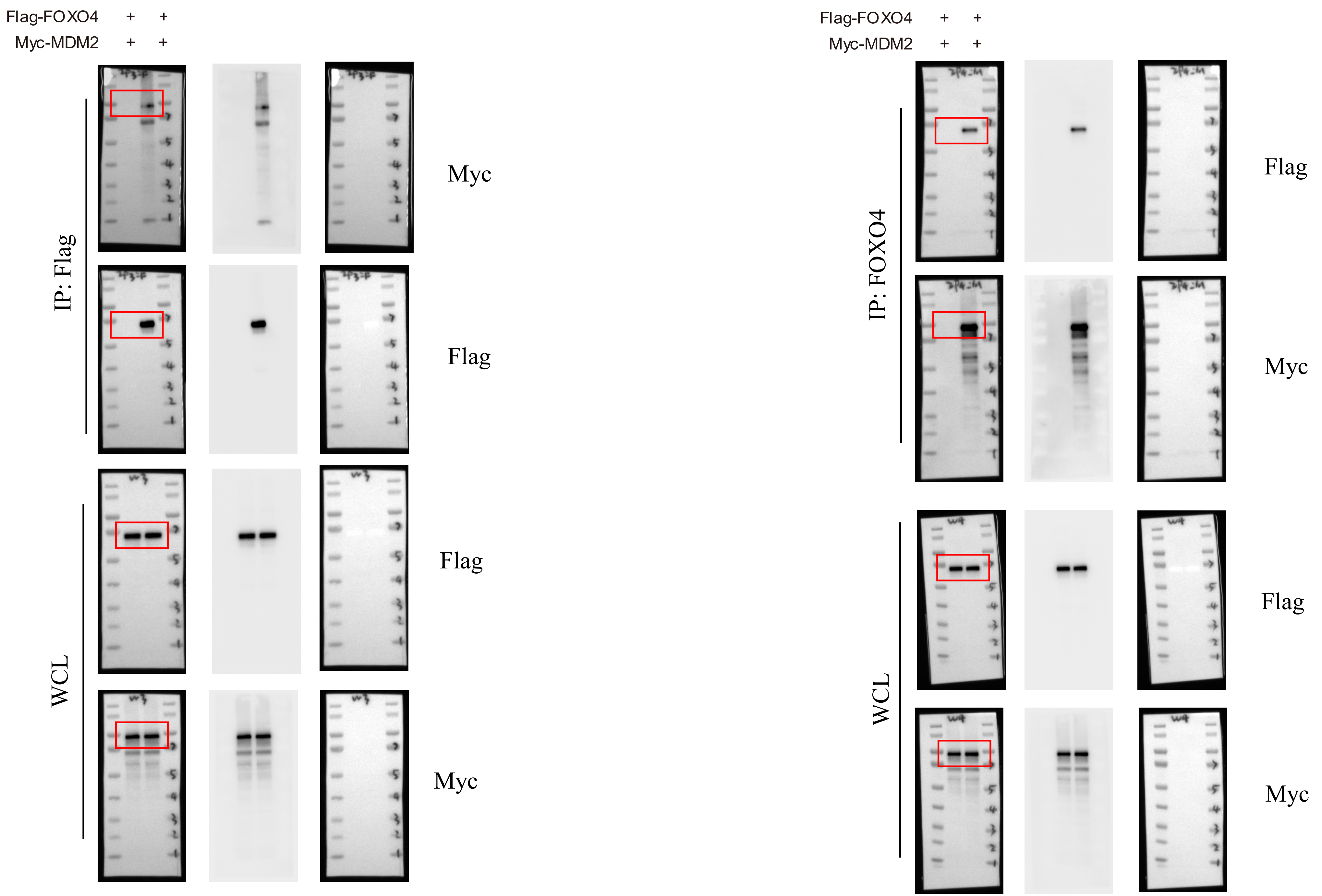
Flag-FOXO4 + +
Myc-MDM2 + +
IP: Flag
Myc
Flag
WCL
Flag
Myc
Flag-FOXO4 + +
Myc-MDM2 + +
IP: FOXO4
Flag
Myc
WCL
Flag
Myc

Fig. S2H

Fig. S2I

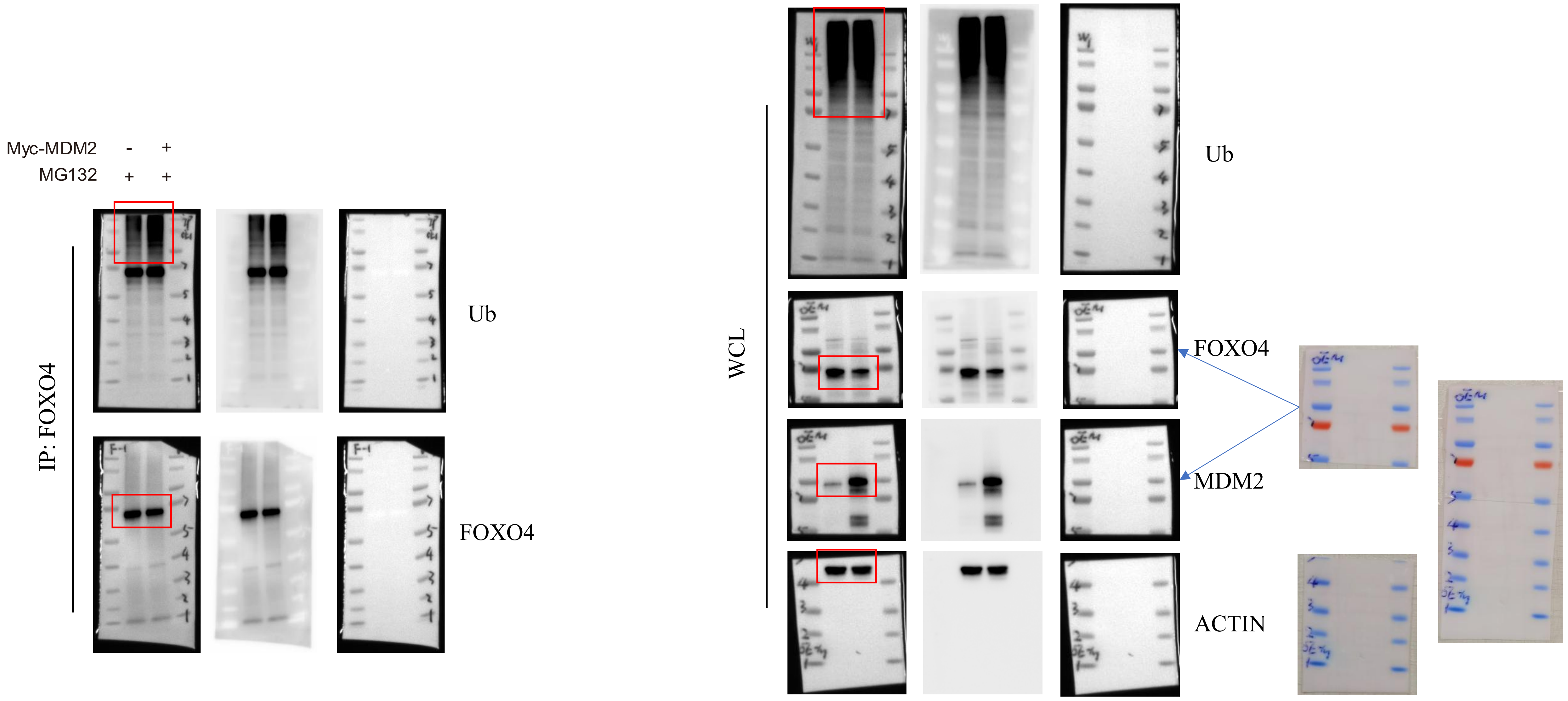

Fig. S2J

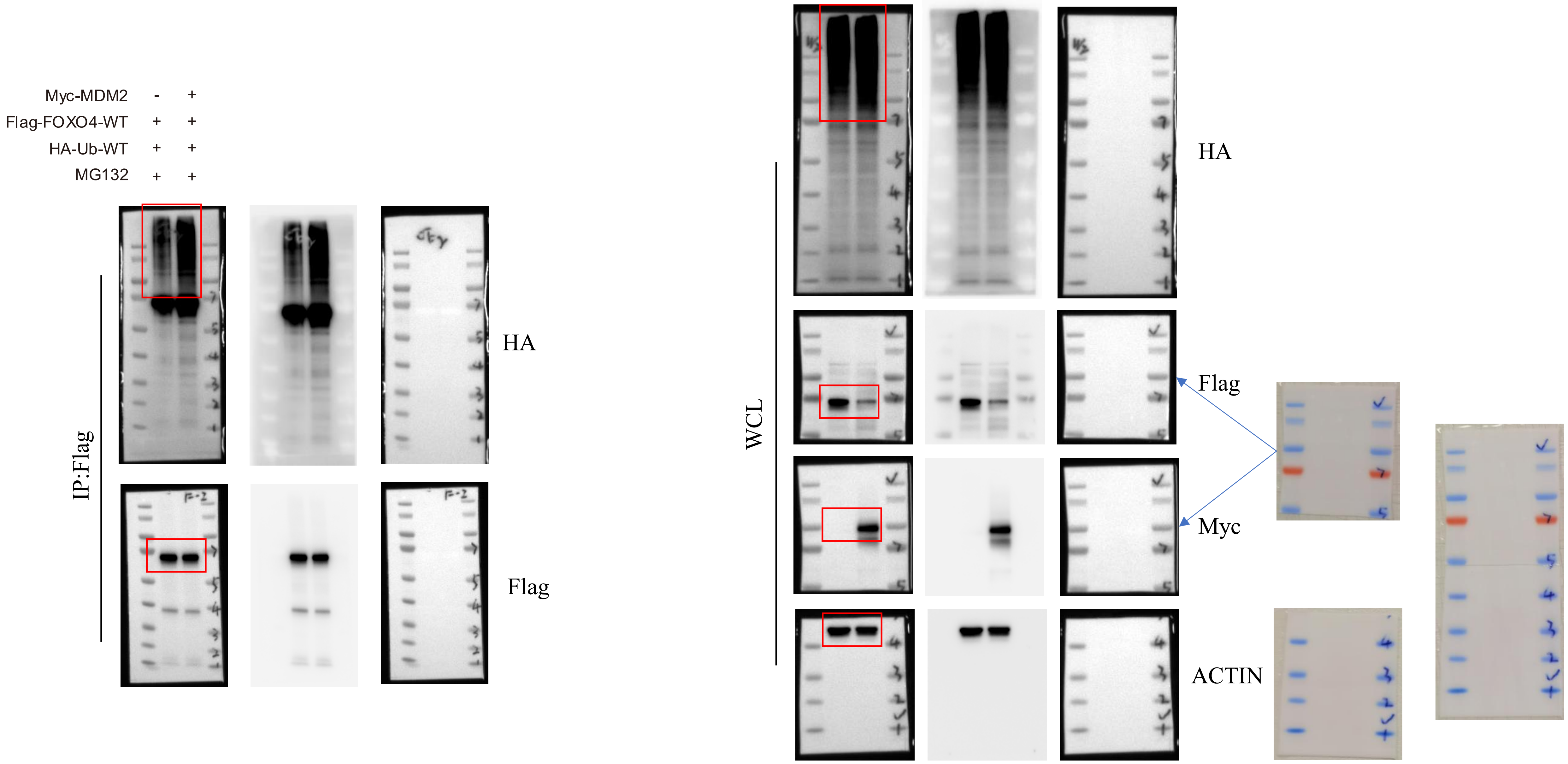
Myc-MDM2 - +
Flag-FOXO4-WT + +
HA-Ub-WT + +
MG132 + +
IP:Flag
HA
Flag
WCL
HA
Flag
Myc
ACTIN

Fig. S2K

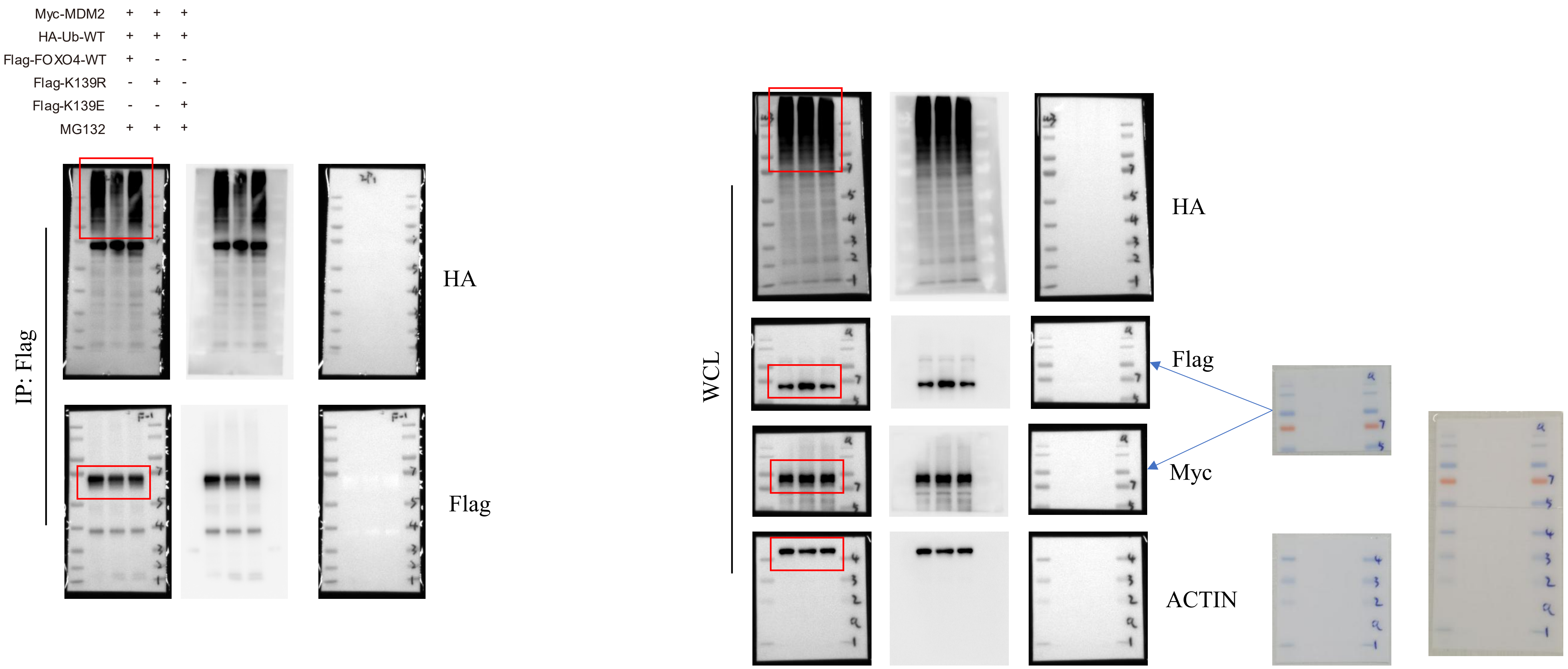

Myc-MDM2 + + +
HA-Ub-WT + + +
Flag-FOXO4-WT + - -
Flag-K139R - + -
Flag-K139E - - +
MG132 + + +
IP: Flag
HA
Flag
WCL
HA
Flag
Myc
ACTIN

Fig. S2L

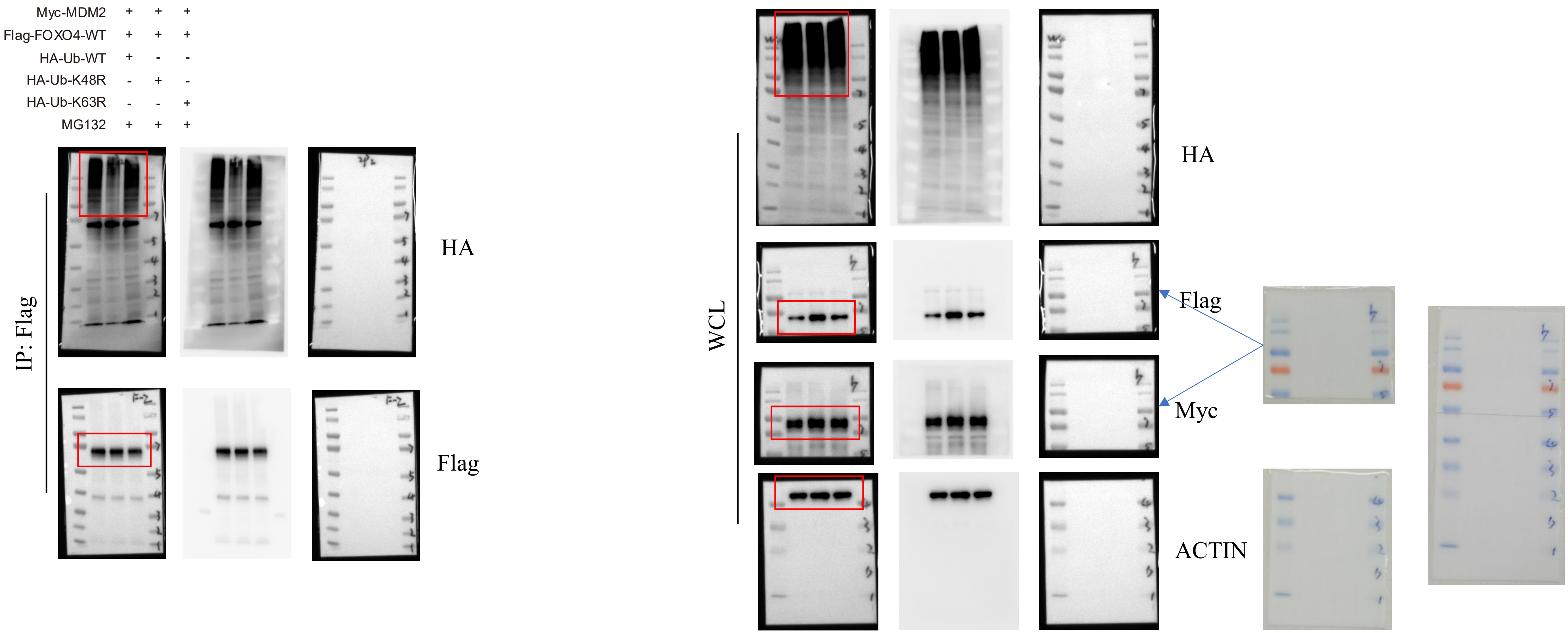
Myc-MDM2 + + +
Flag-FOXO4-WT + + +
HA-Ub-WT + - -
HA-Ub-K48R - + -
HA-Ub-K63R - - +
MG132 + + +
IP: Flag
HA
Flag
WCL
HA
Flag
Myc
ACTIN

Fig. S2M

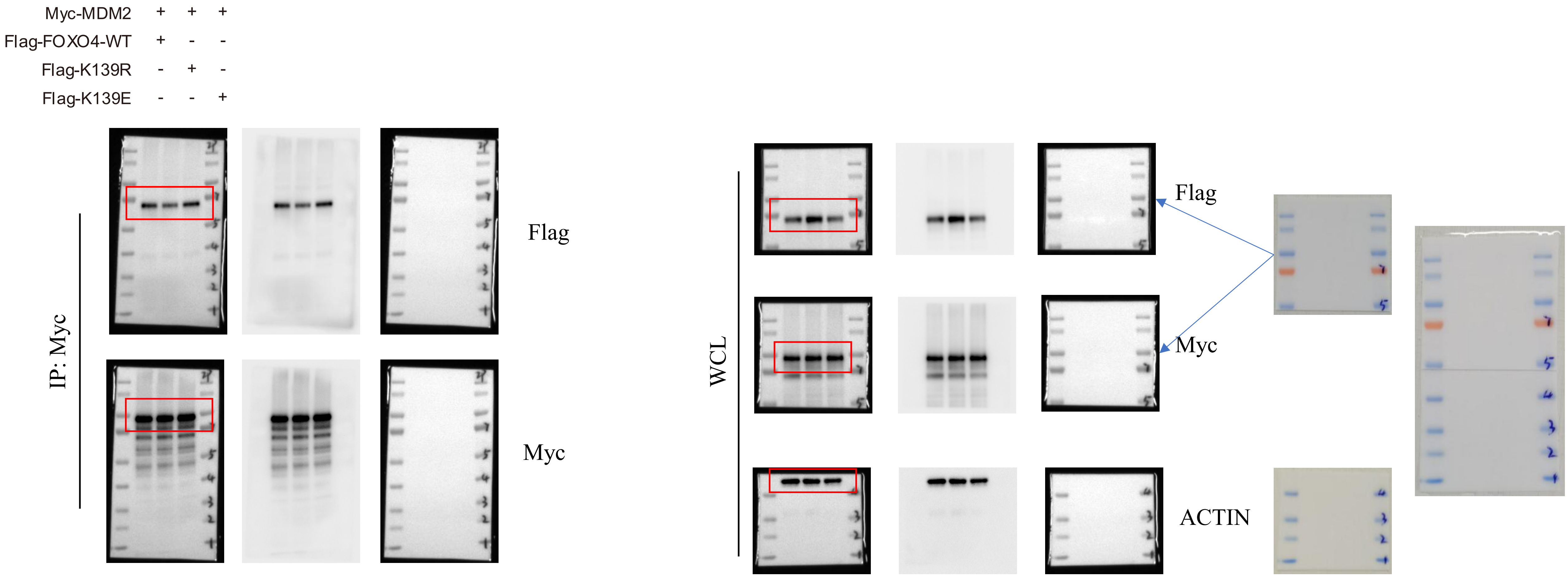
Myc-MDM2 + + +
Flag-FOXO4-WT + - -
Flag-K139R - + -
Flag-K139E - - +
IP: Myc
Flag
Myc
WCL
Flag
Myc
ACTIN

Fig. S3A

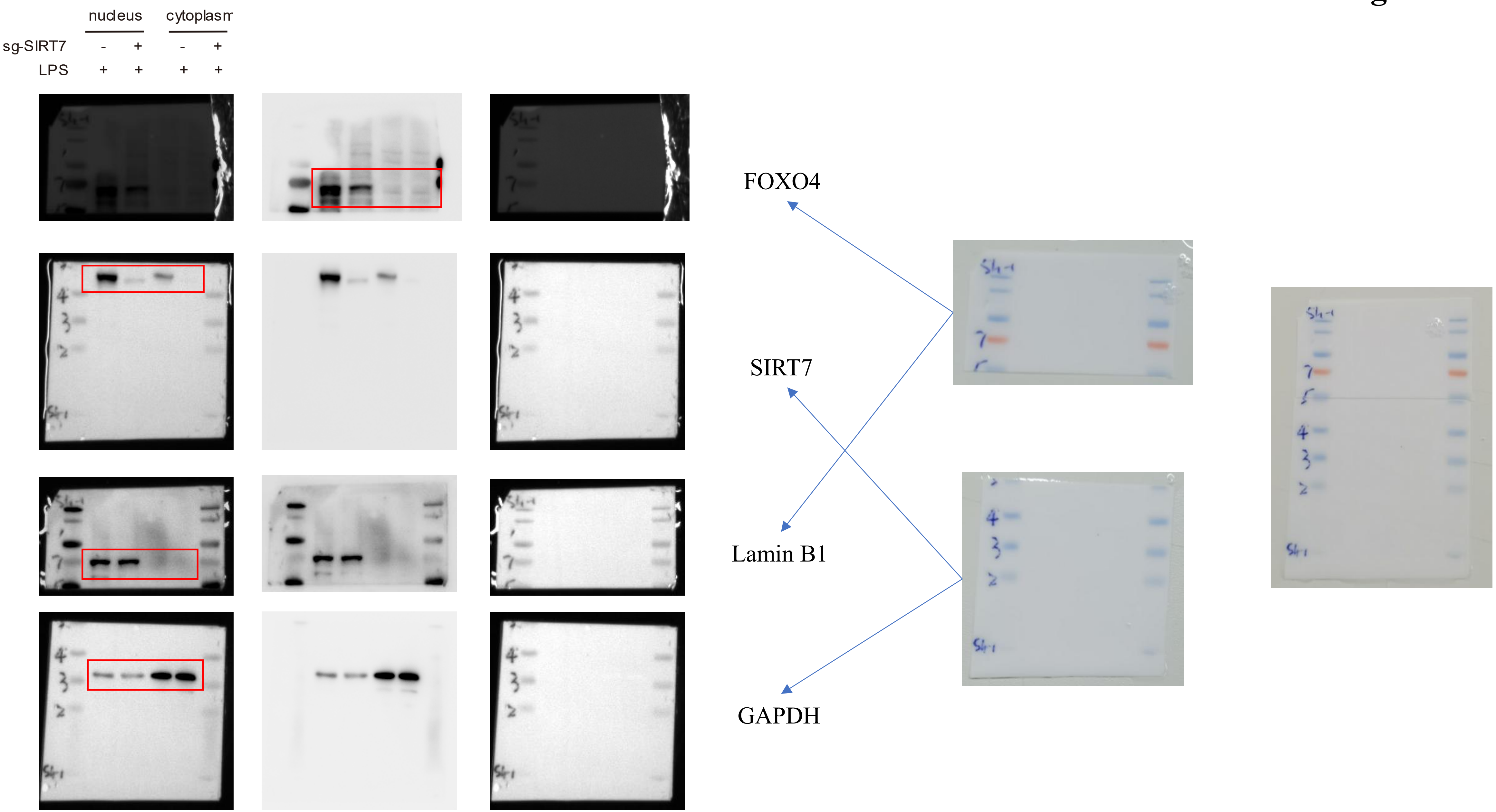

Fig. S3B

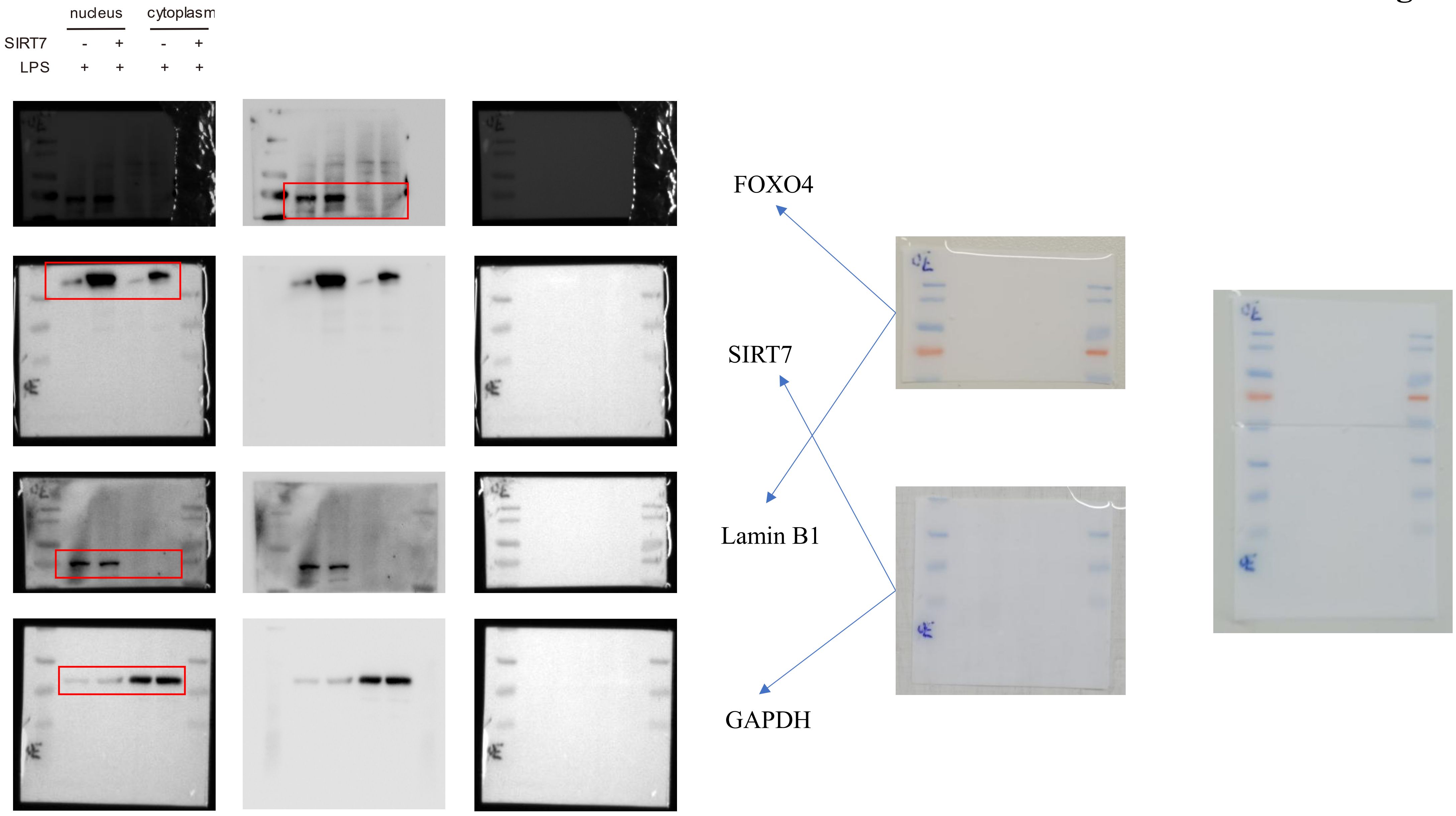

Supplement: Multimedia component 2 [file mmc2.pdf]
